# Death-associated protein kinase 1-dependent SENP1 degradation increases tau SUMOylation and leads to cognitive dysfunction in a mouse model for tauopathy

**DOI:** 10.1186/s13024-025-00911-3

**Published:** 2025-11-21

**Authors:** Xindong Shui, Xiaoqing Zheng, Jinfeng Wu, Mi Zhang, Gamin Kim, Renxuan Chen, Lianlian Peng, Zonghai Wang, Yameng Zheng, Ling Zhang, Ruomeng Li, Long Wang, Ying Zhou, Jungho Kim, Dongmei Chen, Tao Zhang, Tae Ho Lee

**Affiliations:** 1 Fujian Key Laboratory of Cognitive Function and Diseases, Institute of Basic Medicine, School of Basic Medical Sciences, Fujian Medical University, Fuzhou, Fujian China; 2 Key Laboratory of Gastrointestinal Cancer (Ministry of Education), School of Basic Medical Sciences, Fujian Medical University, Fuzhou, Fujian China; 3 Laboratory of Molecular and Cellular Biology, Department of Life Science, Sogang University, Seoul, Korea; 4 Fujian Key Laboratory of Molecular Neurology, Institute of Neuroscience, Fujian Medical University, Fuzhou, Fujian China

**Keywords:** Alzheimer’s disease, Cognition, Death-associated protein kinase 1, Sentrin-specific protease 1, Tauopathy, Tau SUMOylation

## Abstract

**Background:**

Emerging evidence implicates that tau SUMOylation disrupts tau homeostasis. Death-associated protein kinase 1 (DAPK1) has been shown to affect tau phosphorylation and accumulation. The sentrin-specific protease 1 (SENP1) is important for protein SUMOylation, and is a potential substrate of DAPK1. However, whether DAPK1 regulates tau SUMOylation and proteostasis through modulating SENP1 remains elusive.

**Methods:**

We identified the phosphorylation of SENP1 by DAPK1 using in vitro kinase assay and mass spectrometry. The influence of DAPK1 on SENP1 expression, tau SUMOylation and phosphorylation was analyzed using a mouse model for tauopathy by overexpressing human tau in the hippocampal CA3 region, as well as using human AD brain tissues. DAPK1 genetic ablation or pharmacological inhibition was applied to assess the impact of DAPK1 on tau accumulation-related pathologies including synaptic dysfunction and gliosis. The cognitive and emotional functions were evaluated using Y-maze, novel object recognition test, Morris water maze, open field test, and elevated plus maze.

**Results:**

DAPK1 directly interacts with and phosphorylates SENP1, leading to SENP1 degradation via the ubiquitin-proteasome pathway. DAPK1 promotes tau SUMOylation by suppressing SENP1 expression in neurons. DAPK1 downregulation or pharmacological inhibition restores SENP1 level and reduces tau SUMOylation, resulting in an attenuation of aberrant tau phosphorylation and accumulation, which ultimately contributes to improved cognitive ability in vivo. We show that DAPK1 expression is negatively correlated with SENP1 level in human AD hippocampal tissues.

**Conclusions:**

DAPK1-mediated SENP1 phosphorylation and degradation promote tau SUMOylation, exacerbating tau pathology and cognitive dysfunction in tauopathy. Our findings highlight the DAPK1-SENP1-tau SUMOylation axis as a critical regulator of tau homeostasis, and establish DAPK1 inhibition as a promising therapeutic strategy for AD and related tauopathies.

**Supplementary Information:**

The online version contains supplementary material available at 10.1186/s13024-025-00911-3.

## Background

Alzheimer’s disease (AD), the most prevalent form of dementia, is a type of tau-related pathology (tauopathy) [1, 2]. Currently, over 55 million people worldwide are affected by AD or related dementias, making these conditions the leading cause of disability among the elderly [3]. The presence of intracellular neurofibrillary tangles (NFTs) composed of hyperphosphorylated tau aggregates is a pathological hallmark of AD. Impaired tau proteostasis is thought to play a role in NFT formation [4]. In addition to phosphorylation, tau undergoes other post-translational modifications (PTMs), such as ubiquitination and SUMOylation [4–6]. Tau SUMOylation has been shown to promote tau phosphorylation and decrease its ubiquitination-dependent degradation [7]. However, the mechanisms underlying the dysregulation of tau SUMOylation and its contribution to AD pathologies remain poorly understood.

Death-associated protein kinase 1 (DAPK1) is a calcium/calmodulin (Ca²⁺/CaM)-dependent serine/threonine kinase that is broadly expressed in the central nervous system [8–10]. During embryonic development, DAPK1 is highly detectable throughout the brain, with high abundance in cortex, hippocampus and cerebellum. As the brain matures, DAPK1 expression in the cortex gradually declines, while the hippocampal expression remains high [9]. Dysregulation of DAPK1 expression is involved in multiple neurological disorders, including AD, Parkinson’s disease, epilepsy, and traumatic brain injury (TBI) [10–13]. We previously demonstrated that DAPK1 is significantly upregulated in the hippocampi of AD patients compared with those of age-matched controls, which was corroborated by a recent study showing that elevated plasma DAPK1 levels are inversely correlated with cognitive performance in AD cohorts [14–17]. Besides, the expression of DAPK1 is also elevated in brains of transgenic mice for tauopathy [18]. It has been shown that DAPK1 promotes tau hyperphosphorylation by inhibiting Pin1-mediated *cis-*to-*trans* isomerization of the pT231-Pro motif, or by activating the microtubule-affinity regulating kinase 2 (MARK2) [14, 19–22]. Although DAPK1 is known to regulate tau phosphorylation, its role in modulating other tau PTMs and how this contributes to tau pathologies remain unclear.

SUMOylation is a dynamic and reversible process catalyzed by SUMO-specific E1, E2, and E3 ligases and reversed by a family of sentrin-specific proteases (SENPs) [23]. SUMOylation plays a vital role in diverse cellular processes by modulating protein localization, activity, and stability through the covalent conjugation of SUMO molecules to specific lysine residues in target proteins [24, 25]. The dysregulation of SUMOylation has been implicated in diverse pathologies, including neurodegeneration, cancer, and inflammatory disorders [26]. SENP1 is a key cysteine protease responsible for SUMO precursor maturation and substrate deSUMOylation [27]. It is expressed in major brain regions including cerebral cortex, hippocampus and cerebellum [28]. Emerging evidence indicates that SENP1 exerts a neuroprotective effect by suppressing neuroinflammation, inhibiting neuronal cell death and preserving synaptic plasticity through deSUMOylating target proteins [27–33]. Moreover, *Senp1* haploinsufficiency results in autism-like behaviors in murine models by disrupting synaptic excitatory/inhibitory balance [34]. Thus, SENP1 is pivotal for maintaining the structural and functional integrity of the brain. A plethora of SENP1 substrates have been identified in the brain. However, the upstream regulators of SENP1 remain elusive to date.

In this study, we demonstrated that DAPK1 negatively regulates SENP1 level by phosphorylation-dependent degradation. The DAPK1-SENP1 axis critically modulates tau SUMOylation and accumulation in cellular and animal models of tauopathy. DAPK1 knockout (KO) or pharmacological inhibition effectively restores neuronal tau homeostasis by reducing tau SUMOylation, leading to improved neuronal function and cognitive performance in vivo.

## Materials and methods

### Chemicals and reagents

The recombinant adeno-associated virus (rAAV) containing CMV-mCherry-WPRE (vector virus) or CMV-hTau-mCherry-WPRE (hTau virus) was provided by BrainVTA (Wuhan, China). The full-length human tau sequence (2N4R isoform) was used in this study. The proteasome inhibitor MG-132 was purchased from MCE (Shanghai, China). The cycloheximide (CHX) was purchased from Cell Signaling Technology (Danvers, MA, USA). The DAPK1 inhibitor TC-DAPK 6 (C6) was provided by MCE. The protease and phosphatase inhibitor cocktail was obtained from TargetMol (Shanghai, China). Hoechst 33342 and DAPI for cell nuclear staining were acquired from Sangon Biotech (Shanghai, China). N-ethylmaleimide was obtained from Sigma-Aldrich (Darmstadt, Germany). His-Tag and GST-Tag protein purification kits were purchased from Beyotime (Shanghai, China). Protein A/G PLUS-Agarose was obtained from Santa Cruz (Dallas, TX, USA). Anti-fading medium was purchased by Southern Biotech (Birmingham, AL, USA). FreeZol reagent was provided by Vazyme (Nanjing, China). The pECMV-3×FLAG-UBE2I (Flag-Ubc9) plasmid was obtained from MiaoLing Bio (Wuhan, China), and the green fluorescent protein (GFP)-SUMO1 plasmid was provided by Addgene (Cambridge, MA, USA). TurboFect™ Transfection Reagent was purchased from Thermo Fisher Scientific (Waltham, MA, USA).

### Cell culture and treatment

The mouse neuroblastoma N2a cells and human embryonic kidney 293T cells (HEK-293T) were obtained from the Stem Cell Bank/Stem Cell Core Facility (Shanghai, China). The HEK293-hTau cells (293hTau) stably expressing the human tau isoform 2N4R were maintained as previously described [35, 36]. N2a, HEK-293T and 293hTau cells were cultured in high-glucose Dulbecco’s modified Eagle’s medium containing 10% fetal bovine serum, supplemented with 1% penicillin/streptomycin. The isolation and culture of mouse primary neurons were conducted following our previous protocol [17], and neurons on the seventh day in vitro were used for experiments.

### Glutathione S‑transferase (GST) pull-down assay

The GST pull-down analysis was performed according to a previous protocol with slight modifications [19, 37]. HEK-293T cells transfected with target constructs were lysed or diluted in buffer. The cellular supernatants were incubated with purified GST fusion proteins from *E. coli* for about 2 h at 4 °C, after which 25 µL of glutathione-agarose beads were added, and the mixture was incubated for another 2 h at 4 °C. The precipitated proteins were washed with lysis buffer and subjected to immunoblotting analysis or Coomassie blue staining.

### Immunoprecipitation and SUMOylation assay

The immunoprecipitation assay was conducted using established protocols [38]. Briefly, appropriate antibodies were incubated with cell lysates for 3–6 h, followed by protein A/G agarose beads incubation for 2–3 h at 4 °C. The immunoprecipitants were washed with NP-40 lysis buffer, resuspended in 2× loading buffer, and subjected to immunoblotting analysis. To detect protein SUMOylation, we used NP-40 lysis buffer containing 40 mM N-ethylmaleimide to block deSUMOylation.

### In vitro kinase assay and mass spectrometry analysis

The in vitro kinase assay was performed as previously described [37, 39], with minor modifications. Purified recombinant GST-DAPK1 (1-296 aa) was incubated with GST-SENP1 (1-200 aa) or GST-myosin light chain (MLC) in kinase reaction buffer at 37 °C for 30 min. The samples were then subjected to ^32^P autoradiography. For mass spectrometry analysis, samples after in vitro kinase assay were separated by SDS-PAGE and SENP1 bands were collected for the identification of putative phosphorylation sites (Biotech-pack, Beijing, China).

### In vitro deSUMOylation assay

An in vitro deSUMOylation activity assay was carried out as previously described [40]. Recombinant GST-SENP1 and the inactive GST-SENP1 C603S (CS) mutant proteins were purified from *E. coli*. SUMOylated tau was purified from 293hTau cells transfected with GFP-SUMO1 using an anti-tau antibody. After extensive washing with lysis buffer, the immunoprecipitated proteins were divided into aliquots for use as reaction substrates and incubated with either GST-SENP1 or GST-SENP1 C603S mutant in reaction buffer at 37 °C for 20 min. SDS loading buffer was added to stop the reaction, and samples were analyzed by immunoblotting.

### CRISPR/Cas9 gene editing

The pLV3-U6-SENP1(human)-sgRNA2-Cas9-EGFP-Puro plasmid was obtained from MiaoLing Bio and was transfected into HEK-293T cells. GFP-positive cells were sorted by flow cytometry (BD, Franklin Lakes, NJ, USA) after 48 h of transfection and inoculated individually into 96-well plates. After extended culture, candidate monoclonal cell lines were identified by immunoblotting analysis.

### Golgi-Cox staining

Golgi-Cox staining was performed using the FD Rapid GolgiStain™ Kit (FD NeuroTechnologies, Inc., Columbia, MD, USA) according to the manufacturer’s protocol. Coronal sections (150 μm) were prepared with a vibratome (Leica, Wetzlar, Germany). Images were acquired using a Zeiss primo star microscope with a 100× oil objective. Dendritic spines were counted using ImageJ software (version 1.51j8).

### Immunoblotting analysis

The immunoblotting analysis was conducted according to previous reports [17, 41]. In brief, total protein was extracted from mouse hippocampal or cell samples using radioimmunoprecipitation assay lysis buffer containing protease and phosphatase inhibitors. Protein concentration was determined using a BCA protein assay kit (Beyotime). Protein samples were separated by SDS/PAGE and transferred to polyvinylidene fluoride membranes (Merck, Darmstadt, Germany) using either wet or semi-dry transfer system (Bio-Rad, Hercules, CA, USA). Membranes were blocked with 5% bovine serum albumin-TBST or 5% milk-TBST for 1 h at room temperature. Primary antibodies were incubated overnight at 4 °C. After washing, horseradish peroxidase (HRP)-conjugated secondary antibodies were applied for 1 h at room temperature. Signals were visualized using a ChemiDoc Imaging System (Bio-Rad). Antibody specifications are listed in Table S1. The densitometry was performed using ImageJ. β-actin was used as internal control and data were presented by normalizing to the corresponding control group.

### Quantitative RT-PCR (qRT-PCR) assay

Total RNA was isolated from N2a, 293hTau cells and tissues using the FreeZol reagent. Reverse transcription was then carried out using a Transcriptor First Strand cDNA Synthesis Kit (Roche, Indianapolis, IN, USA). qRT-PCR was conducted using FastStart Universal SYBR Green Master Mix (Roche) in a QuantStudio Real-Time PCR system (Applied Biosystems, Waltham, MA, USA) under the following thermocycling conditions: 95 °C for 3 min followed by 40 cycles of 95 °C for 20 s, 60 °C for 35 s, and 72 °C for 30 s. Each sample was analyzed in duplicate. Gene expression was quantified using the 2^−ΔΔCt^ method and normalized to the β-actin level. The primers used are shown in Table S2.

### Ubiquitination assay

The ubiquitination assay was performed as previously described with slight modifications [17, 38]. In brief, HEK-293T cells were transiently transfected with Flag-DAPK1, HA-SENP1 or its mutants, and His-ubiquitin. Cells were collected 36 h after transfection, snap-frozen in liquid nitrogen and stored at − 80 °C overnight. The cell lysates were prepared with urea buffer at 4 °C for 30 min, followed by sonication. The cellular supernatants were incubated with Ni-NTA agarose (Thermo Fisher Scientific) for 3 h at 4 °C. The agarose beads were pelleted and subjected to immunoblotting analysis.

### Immunofluorescence

The immunofluorescence imaging was performed as previously described with slight modifications [36, 41]. Paraffin-embedded brain tissues were cut into sections for histological analysis. Following deparaffinization, rehydration, and heat-mediated antigen retrieval, sections were blocked in goat serum (Solarbio, Beijing, China) at room temperature for 1 h. Cells were fixed with 4% paraformaldehyde for 20 min at room temperature. After permeabilization, cells were blocked in goat serum at room temperature for 1 h. Primary antibodies were applied to brain sections or cell samples at 4 °C overnight. The next day, samples were rinsed in PBS and were incubated with Alexa Fluor 488- or 546-conjugated secondary antibodies for 1 h at room temperature in the dark. Nuclei were counterstained with Hoechst 33342 for 10 min. Samples were then covered with anti-fading medium. Images were acquired using a conventional fluorescence microscope (Zeiss Axio Imager 2, Oberkochen, Germany).

### Immunohistochemistry

Immunohistochemistry was conducted as described previously [38]. After deparaffinization, rehydration and antigen retrieval, endogenous peroxidase activity was blocked by incubating brain sections in 0.3% H_2_O_2_ at 37 ℃ for 25 min before being blocked with goat serum at room temperature for 1 h and incubated with the primary antibody in PBS containing 10% goat serum at 4 °C overnight. Tissue sections were then incubated with HRP-conjugated secondary antibody (Boster, Wuhan, China) for 1 h at room temperature in the dark. The slides were incubated with DAB reagents (MXB Biotechnologies, Fuzhou, China) after rinsing. The sections were visualized using a digital microscope (ECHO, San Diego, CA, USA).

### Animals, stereotaxic brain injection of rAAV virus and treatment

Seven-week-old male C57BL/6 mice were acquired from Shanghai laboratory animal center (Shanghai, China), and housed in a standard specific pathogen-free environment at the animal facility. Mice were maintained on a 12-hour light-dark cycle with unrestricted access to standard chow diet (HFK bioscience, Beijing, China) and water. Following one week of acclimation, the mice were subjected to stereotaxic surgery for viral infusion, as previously detailed [36, 42]. Briefly, the mice were anesthetized via isoflurane inhalation and secured in a stereotaxic apparatus. After sterilization with iodophor, a surgical incision was made along the anterior-posterior axis to expose the cranium. A hand-held drill was used to create a hole at the following coordinates: AP -2.2 mm, ML -2.7 mm, and DV -2.3 mm relative to the bregma. The hTau (2N4R isoform) or vector virus (5.7 × 10^12^ viral genomes/mL) was infused into the CA3 region using a microsyringe pump (Kd Scientific, Holliston, MA, USA) at a rate of 125 nL/min, culminating in a total volume of 700 nL in each injection. The injection needle was retained in place for 8 min post-infusion before removal. The incision was subsequently sutured, and the mouse was placed on a heating pad for recovery. Body weight measurements were conducted once every two weeks. The generation of DAPK1-KO mice on the C57BL/6 background was described previously [43]. Age-matched male wild-type (WT) and DAPK1-KO mice were used in the study. All animal experiments were reviewed and approved by the Experimental Animal Ethics Committee of Fujian Medical University (IACUC FJMU 2022-Y-0547).

### Behavioral tests

Prior to the behavioral tests, mice were handled by the operator for 3–5 min per day for at least three consecutive days. Mice were transferred to the behavioral testing room approximately 1 h before the tests.

### Morris water maze (MWM)

MWM was employed to analyze spatial learning and memory according to previous studies [36, 39, 44]. In the water maze (diameter 120 cm, depth 50 cm) equipped with a camera (RWD, Shenzhen, China), mice were trained in a visible platform test (day one) and a hidden platform test (days two to five) consecutively, with five trials per day (intertrial interval of approximately 20 min). In the training sessions, if a mouse reached the platform within 60 s, it was permitted to stay on the platform for 5 s. Otherwise, the mouse was guided to the platform and allowed to remain there for 20 s before being returned to its original cage. On day six, the platform was removed for the probe test, and the mice were allowed to swim freely in the water for 60 s. The mice were gently placed in the water from the starting position, facing the wall of the water maze. The data were analyzed using Smart software (version 3.0.06) from Panlab Harvard Apparatus (Barcelona, Spain).

### Y-maze test

The Y-maze test assessed the short-term spatial memory in mice [13]. The test was performed in an apparatus consisting of three identical arms (38 cm × 8 cm × 16.5 cm) at 120° angles from each other. During the training phase, each mouse was placed at the end of one arm; the second arm blocked as the novel arm (NA), and the third arm was left open as the familiar arm (FA). Mouse was allowed to explore for 5 min. The apparatus was cleaned with 75% ethanol after each trial. In the test phase, all three arms were kept open, and mice were allowed to explore freely for 3 min. Movement in the Y-maze was recorded with a camera, and the number of entries into and the time spent in NA were analyzed using Smart software.

### Open field test (OFT)

The OFT was performed based on a previous protocol with minor modifications [45]. Each mouse was allowed to freely explore the open area (40 cm × 40 cm arena) for 5 min. After each test, the arena was cleaned with 75% ethanol. The distance traveled and time spent by each mouse in the central region were analyzed using Smart software.

### Novel object recognition (NOR) test

The NOR test was performed using the same apparatus as the OFT. During the habituation session, each mouse was placed in the arena and allowed to explore for 10 min. During the training session, two identical objects (named A and B) were put into the arena, and each mouse was placed at the same starting point and allowed to freely explore two objects for 5 min. After 24 h, the object A was replaced by a new object C, and each mouse was reintroduced into the arena, given 5 min to explore both objects. Exploratory behavior was recorded using a camera. The time each mouse spent for each object was calculated by Smart software and the recognition index was calculated [46].

### Elevated plus maze (EPM) test

The EPM test was performed according to a previous method [13]. The EPM consists of two open arms (30 cm × 5 cm) and two closed arms (30 cm × 5 cm) extending outward from a center platform (the decision area) to form a plus shape. The apparatus was elevated 50 cm above the ground. Each mouse was placed in the center area and allowed to explore freely for 5 min. The maze was wiped with 75% ethanol after each test. The movement of each mouse was recorded and analyzed using Smart software. The percentage of time each mouse spent in the closed arms and the open arms was calculated.

### Nesting test

The nesting test was conducted as reported, with slight modifications [47]. Briefly, mice were individually housed in new cages for 24 h. Paper strips (5 cm long × 1 cm wide) were placed in each cage at 6:00 PM. Nest construction was evaluated and scored in a blinded manner following a 5-point Deacon scale [48].

### Human brain tissues

Hippocampal brain tissues from both human AD patients and control subjects were subjected to immunoblotting and immunohistochemical staining analyses. Most of the brain tissue samples used in this study were obtained within 30 h postmortem. Detailed information regarding the human samples can be found in Tables S3 and S4. The protocol was reviewed and approved by the Ethics Committee of Fujian Medical University (2022-95).

### Statistical analysis

Statistical analysis was carried out using GraphPad Prism software (version 8.3.0). The values are expressed as the means ± standard deviation (SD) obtained from triplicate experiments. The data were first checked by the Shapiro-Wilk normality test. A two-tailed unpaired *t*-test was used to detect the difference between two groups, while multiple comparisons were performed using the one-way analysis of variance (ANOVA) followed by Tukey’s *post-hoc* test for samples over two groups. The specifications regarding group size and the statistical methods employed are provided in the figure legends. A *p* value < 0.05 was considered statistically significant.

## Results

### DAPK1 directly binds to SENP1 in vitro and in vivo

**Fig. 1:**
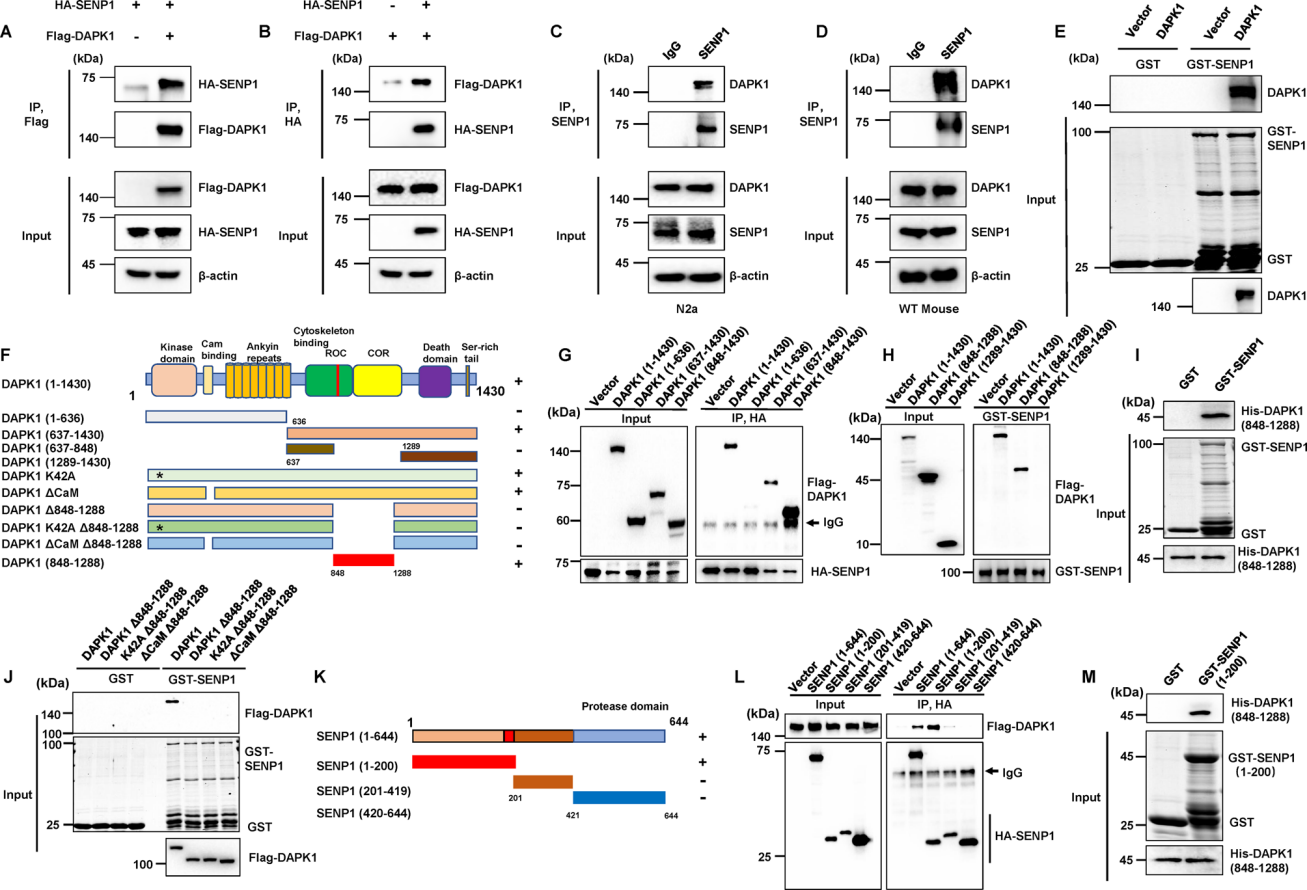
DAPK1 interacts with SENP1 in vitro and in vivo. (**A**-**B**) Co-immunoprecipitation analyses of the binding between DAPK1 and SENP1 in HEK-293T cells transfected with Flag-DAPK1 and HA-SENP1 using indicated antibodies. (**C**-**D**) Co-Immunoprecipitation analyses of the binding between endogenous DAPK1 and SENP1 in N2a cells and mouse brain using indicated antibodies. (**E**) Cell lysates from HEK-293T cells expressing Myc-vector or Myc-DAPK1 were subjected to GST pull-down assay with GST or GST-SENP1. The pull-down samples were detected using an anti-Myc antibody. (**F**) Schematic of the structural domains of full-length DAPK1 and truncated mutants used in the present study. Asterisk (*) indicates the Lys42 to Ala (K42A) mutation site in DAPK1. Plus (+) indicates positive for binding to SENP1, while minus (-) indicates negative for binding to SENP1. **(G)** Co-immunoprecipitation analyses of the binding between SENP1 and full-length DAPK1 or various truncated mutants using indicated antibodies. **(H)** Cell lysates from HEK-293T cells expressing full-length DAPK1 or 848–1288 aa or 1289–1430 aa domains were subjected to pull-down assay with GST-SENP1. The pull-down samples were detected using an anti-Flag antibody. **(I)** Purified His-DAPK1 (848–1288 aa) was incubated with GST or GST-SENP1, followed by immunoblotting analysis using an anti-His antibody. **(J)** Flag-DAPK1 or its mutants (K42A and ΔCaM) with the deletion of the 848–1288 aa domain was expressed in HEK-293T cells. Cell lysates were then incubated with GST or GST-SENP1 for pull-down assay, followed by immunoblotting analysis using indicated antibodies. **(K)** Schematic of the structural domains of full-length SENP1 and truncated mutants used in the present study. Plus (+) indicates positive for binding to DAPK1, while minus (-) indicates negative for binding to DAPK1. **(L)** Flag-DAPK1 was co-expressed with HA-SENP1 or the truncated mutants in HEK-293T cells. Cell lysates were immunoprecipitated with an anti-HA antibody, and DAPK1 was probed using an anti-Flag antibody. **(M)** Purified His-DAPK1 (848–1288 aa) was incubated with GST or GST-SENP1 (1-200 aa), followed by immunoblotting analysis using an anti-His antibody. Representative images from triplicate repeats are shown

We previously identified candidate substrates for DAPK1 using an unbiased peptide library screening method and found that SENP1 may be a substrate of DAPK1 [37]. DAPK1 is functionally involved in regulating tau homeostasis by modulating tau phosphorylation [10, 14, 49]. Interestingly, tau SUMOylation is tightly associated with tau phosphorylation and degradation, and SENPs have been reported to participate in regulating protein SUMOylation in AD [50]. Thus, DAPK1 and SENP1 might be interconnected in the pathogenesis of AD. To test this hypothesis, we first examined whether SENP1 binds to DAPK1 in cells. The co-immunoprecipitation results showed that SENP1 interacted with DAPK1 (Fig. 1A, B). Moreover, endogenous binding between DAPK1 and SENP1 was validated in N2a cells and mouse brain tissues (Figs. 1C, D and S1A, B). GST pull-down experiments further corroborated the direct interaction between DAPK1 and SENP1 (Fig. 1E). Both the kinase-impaired mutant DAPK1-K42A and the constitutively active mutant DAPK1 ΔCaM showed equal binding to SENP1 (Fig. S1C, D), indicating that the kinase activity of DAPK1 is not required for SENP1 interaction. Immunofluorescence staining further confirmed that SENP1 colocalized with DAPK1 or its mutants in the cytoplasm in a kinase activity-independent manner (Fig. S1E-H). These results indicate that DAPK1 is associated with SENP1.

To identify the structural domains involved in the interaction between SENP1 and DAPK1, a series of truncated DAPK1 constructs were generated and used for co-immunoprecipitation and GST pull-down assays (Fig. 1F). DAPK1 interacted with SENP1 via a domain encompassing the 848–1288 aa (Figs. 1G-I and S1I-J), which mainly contains the C-terminus of the ROC (COR) domain of DAPK1 (Fig. 1F). The deletion of 848–1288 aa of DAPK1 (Δ848–1288) abolished the binding between DAPK1 and SENP1, according to GST pull-down and co-immunoprecipitation assays (Figs. 1J and S1K). To identify which part of SENP1 is involved in the interaction with DAPK1, we conducted a GST pull-down assay using SENP1 truncation variants (Fig. 1K), and found that the SENP1 N-terminal region (1-200 aa) was capable of interacting with DAPK1 (Figs. 1 L and S1L). Furthermore, we confirmed direct binding between the 848–1288 aa domain of DAPK1 and the N-terminal region (1-200 aa) of SENP1 using purified recombinant proteins (Fig. 1M). Thus, these two domains are indispensable for the interaction between DAPK1 and SENP1.

### DAPK1 promotes SENP1 protein degradation via the ubiquitin-proteasome pathway

**Fig. 2:**
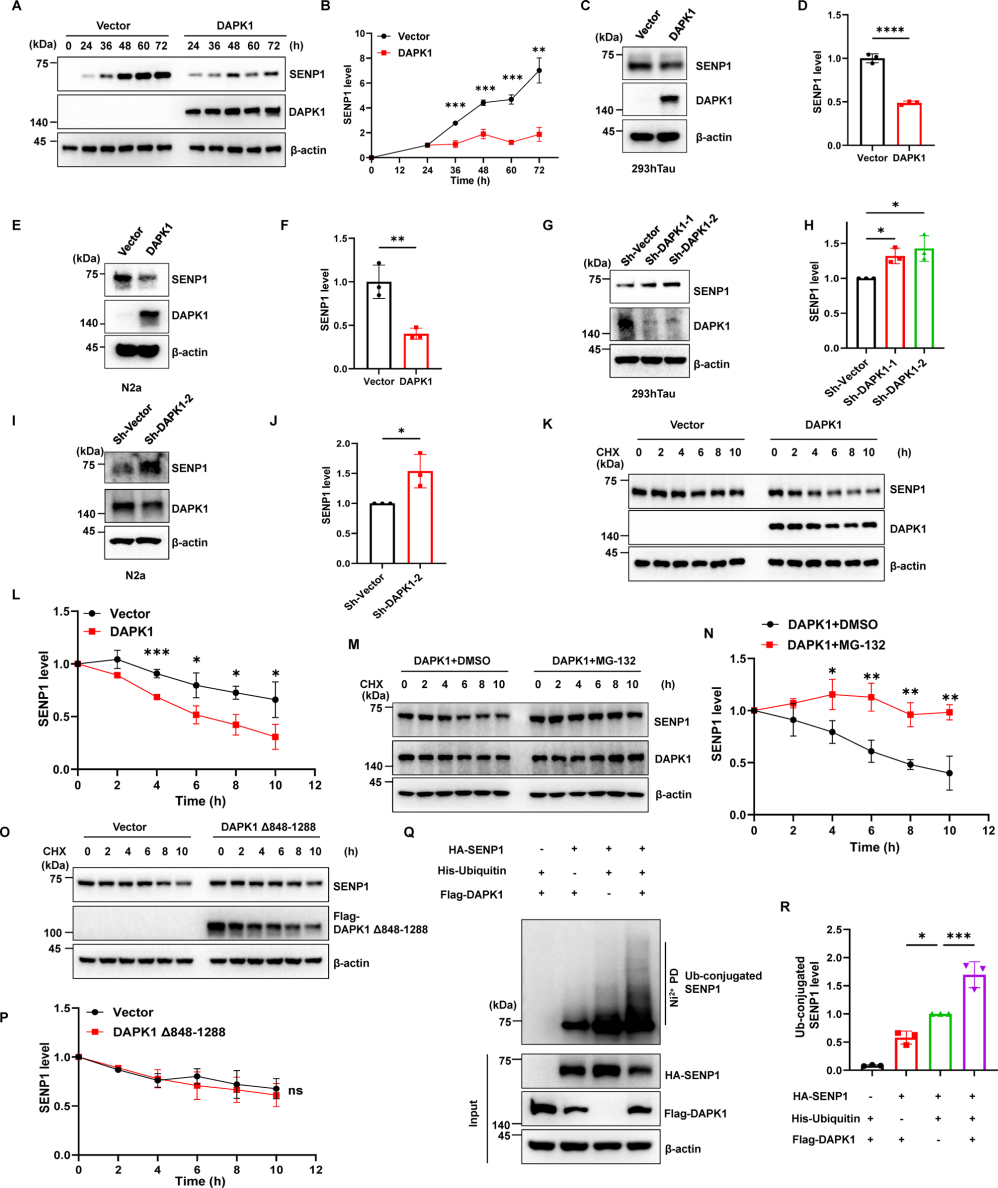
DAPK1 regulates the protein stability of SENP1 **(A-B)** HA-SENP1 was co-expressed with or without Flag-DAPK1 in HEK-293T cells for indicated time points. Cell lysates were collected and subjected to immunoblotting analysis using indicated antibodies. **(C-F)** 293hTau (C-D) or N2a (E-F) cells were transfected with vector or Flag-DAPK1 constructs, and samples were subjected to immunoblotting analysis using indicated antibodies. **(G-J)** 293hTau (G-H) or N2a (I-J) cells were transfected with control or DAPK1 shRNAs, and samples were subjected to immunoblotting analysis using indicated antibodies. **(K-L)** HA-SENP1 was co-transfected with or without Flag-DAPK1 in HEK-293T cells, and then treated with 50 µg/mL CHX for indicated time. Samples were subjected to immunoblotting analysis using indicated antibodies. **(M-N)** HA-SENP1 was co-transfected with Flag-DAPK1 in HEK-293T cells, and treated with 12.5 µM MG-132 for 30 min. Then 50 µg/mL CHX was added for indicated time. Samples were subjected to immunoblotting analysis using indicated antibodies. **(O-P)** HA-SENP1 was co-transfected with or without Flag-DAPK1 lacking the 848–1288 aa domain (Δ848–1288) in HEK-293T cells, and then treated with 50 µg/mL CHX for indicated time. Samples were subjected to immunoblotting analysis using indicated antibodies. **(Q-R)** HA-SENP1 was expressed in HEK-293T cells with or without Flag-DAPK1 and His-ubiquitin for 48 h, and ubiquitin-conjugated proteins were precipitated with Ni-beads and subjected to immunoblotting analysis using indicated antibodies. Representative images from triplicate repeats are shown. *p < 0.05, **p < 0.01, ***p < 0.001, ****p < 0.0001, ns, not significant. Two-tailed unpaired *t*-test was used in B, D, F, J, L, N and P, and one-way ANOVA followed by Tukey’s *post-hoc* test was used in H and R

The dysregulation of SENP1 is closely involved in major neurological diseases, including ischemic stroke and autism spectrum disorder [28, 34]. Since DAPK1 is also dysregulated in various brain disorders such as stroke and AD [15, 51], and because DAPK1 and SENP1 directly bind, we wondered whether DAPK1 can affect SENP1 function. In HEK-293T cells, DAPK1 overexpression significantly reduced exogenous SENP1 levels throughout the observation time period (Fig. 2A, B). DAPK1 overexpression suppressed endogenous SENP1 protein expression in 293hTau (Fig. 2C, D) and N2a cells (Fig. 2E, F). In contrast, DAPK1 knockdown using short hairpin RNAs (shRNAs) increased the level of SENP1 in 293hTau and N2a cells (Fig. 2G-J). Critically, neither DAPK1 overexpression nor its knockdown altered *SENP1* mRNA levels (Fig. S2), suggesting that DAPK1 regulates SENP1 expression post-translationally. We thus treated cells with CHX to inhibit new protein synthesis and evaluate SENP1 protein stability. SENP1 protein degraded faster in DAPK1-overexpressing cells than in control cells, indicating that DAPK1 decreases SENP1 protein stability (Fig. 2K, L). However, proteasome inhibition by MG-132 abrogated the effect of DAPK1 on SENP1 degradation (Fig. 2M, N). DAPK1 mutant lacking the 848–1288 aa domain (Δ848–1288) or the 848–1288 aa domain alone could not regulate SENP1 protein stability (Fig. 2O, P and Fig. S3), indicating that the binding itself does not affect SENP1 degradation. To further study whether DAPK1 promotes the ubiquitination of SENP1 in cells, we performed a ubiquitination assay by transfecting DAPK1, SENP1 or His-ubiquitin into cells. The ubiquitination of SENP1 was low in cells lacking DAPK1, while DAPK1 overexpression promoted the polyubiquitination of SENP1 (Fig. 2Q, R). These results suggest that DAPK1 reduces SENP1 expression by promoting ubiquitin-dependent protein degradation.

### DAPK1-dependent SENP1 phosphorylation at Ser126 promotes SENP1 degradation

**Fig. 3:**
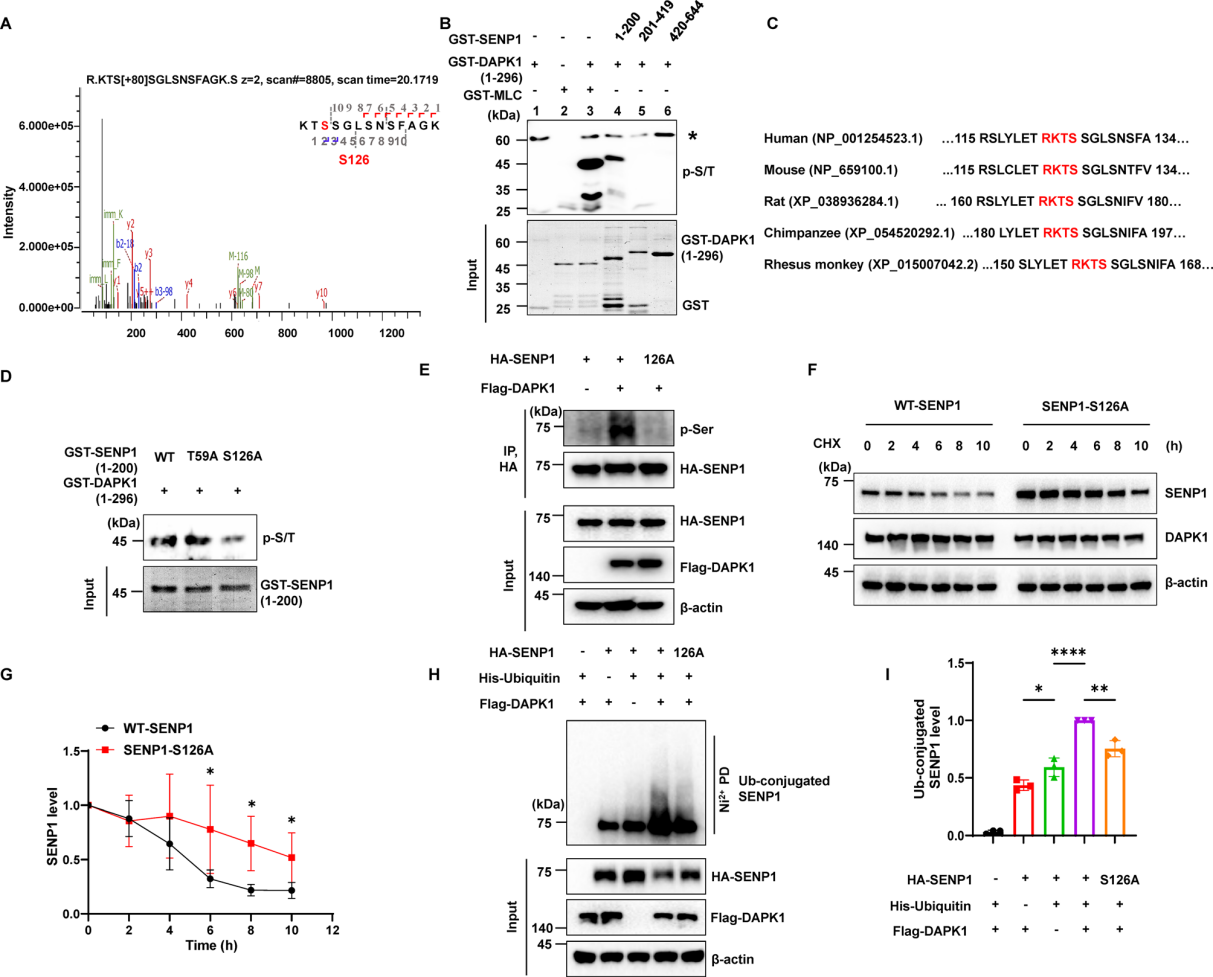
DAPK1-mediated phosphorylation on Ser126 promotes SENP1 degradation **(A)** The mass spectrum of SENP1 phosphorylation at Ser126. GST-SENP1 was subjected to an in vitro phosphorylation assay, and samples were collected for mass spectrometry analysis. **(B)** An in vitro phosphorylation assay was performed by mixing GST-DAPK1 (1-296 aa) with GST-MLC or GST-SENP1 fragments at 37 °C for 30 min. The phosphorylation signal was detected using an anti-pSer/Thr antibody (p-S/T). Asterisk (*) indicates the autophosphorylation of GST-DAPK1 (1-296 aa). **(C)** Sequence alignment of SENP1 from multiple species showing the presence of a conserved RKTS motif (highlighted in red) containing the putative DAPK1 phosphorylation site in different species. **(D)** An in vitro phosphorylation assay was performed by mixing GST-DAPK1 (1-296 aa) with GST-SENP1 (1-200 aa), or the Thr59 to Ala (T59A) or Ser126 to Ala (S126A) mutant at 37 °C for 30 min. The phosphorylation signal was detected using an anti-pSer/Thr antibody (p-S/T). **(E)** HA-SENP1 or the S216A mutant construct was expressed in HEK-293T cells with Flag-DAPK1, and an anti-HA antibody was used for immunoprecipitation of SENP1 proteins. Then the phosphorylation of SENP1 was detected using an anti-phospho-Serine (pSer) antibody. **(F-G)** HA-SENP1 or the S126A mutant construct was expressed in HEK-293T cells with Flag-DAPK1, and 50 µg/mL CHX was added for indicated time. Samples were subjected to immunoblotting analysis using indicated antibodies. **(H-I)** HA-SENP1 or the S126A mutant construct was expressed in HEK-293T cells with Flag-DAPK1 and His-ubiquitin for 48 h. The ubiquitin-conjugated proteins were precipitated with Ni-beads and subjected to immunoblotting analysis using indicated antibodies. Representative images from triplicate repeats are shown. *p < 0.05, **p < 0.01, ****p < 0.0001. Two-tailed unpaired *t*-test was use in G, and one-way ANOVA followed by Tukey’s *post-hoc* test was used in I

Since DAPK1 has been shown to regulate the function of its substrates via phosphorylation, we wondered whether DAPK1 phosphorylates SENP1 and affects its level and function. We first performed in vitro kinase assays followed by mass spectrometry analyses. The GST-DAPK1 (1-296 aa) contains the kinase domain essential for catalyzing substrate phosphorylation, and is capable of phosphorylating well-known DAPK1 substrates including the N-myc downstream-regulated gene 2 and Pin1 in vitro [19, 37]. The analysis identified Ser126 and Thr59 as potential SENP1 phosphorylation sites by DAPK1 (Figs. 3A and S4). Using an anti-phospho-Ser/Thr (p-S/T) antibody, we observed that both SENP1 (1-200 aa) and the positive control MLC were phosphorylated by DAPK1 (Fig. 3B), indicating that the N-terminal region of SENP1 might be phosphorylated by DAPK1. The Ser126 residue of SENP1 is conserved across different species and matches with the consensus R-X-X-S/T phosphorylation motif of DAPK1 [37], whereas the Thr59 residue is not (Fig. 3C). Point mutations of SENP1 (1-200 aa) at Thr59 or Ser126 were subjected to in vitro kinase assay, and the SENP1-S126A mutant but not the T59A mutant showed a remarkable decrease in the phosphorylation (Fig. 3D), suggesting that DAPK1 likely phosphorylates SENP1 at Ser126. In HEK-293T cells, WT SENP1 but not the SENP1-S126A mutant was phosphorylated by DAPK1 (Fig. 3E), which is consistent with the data from ^32^P autoradiography assay (Fig. S5). The presence of residual phosphorylation signals in the in vitro kinase assays by using anti-p-S/T antibody or ^32^P autoradiography suggests that there might be additional sites that could be phosphorylated by DAPK1. Nevertheless, DAPK1-induced S126 phosphorylation has a major role in regulating the expression of SENP1 in cells. To verify this, we analyzed the stability of WT SENP1 or SENP1-S126A mutant in the presence of DAPK1. The degradation of the SENP1-S126A mutant was slower than that of WT SENP1 (Fig. 3F, G). Moreover, the SENP1-S126A mutant also showed reduced ubiquitination compared with WT SENP1 (Fig. 3H, I). The above data prove that DAPK1-induced SENP1 phosphorylation at Ser126 is associated with SENP1 protein stability and degradation.

### DAPK1 induces tau SUMOylation by regulating SENP1 expression

**Fig. 4:**
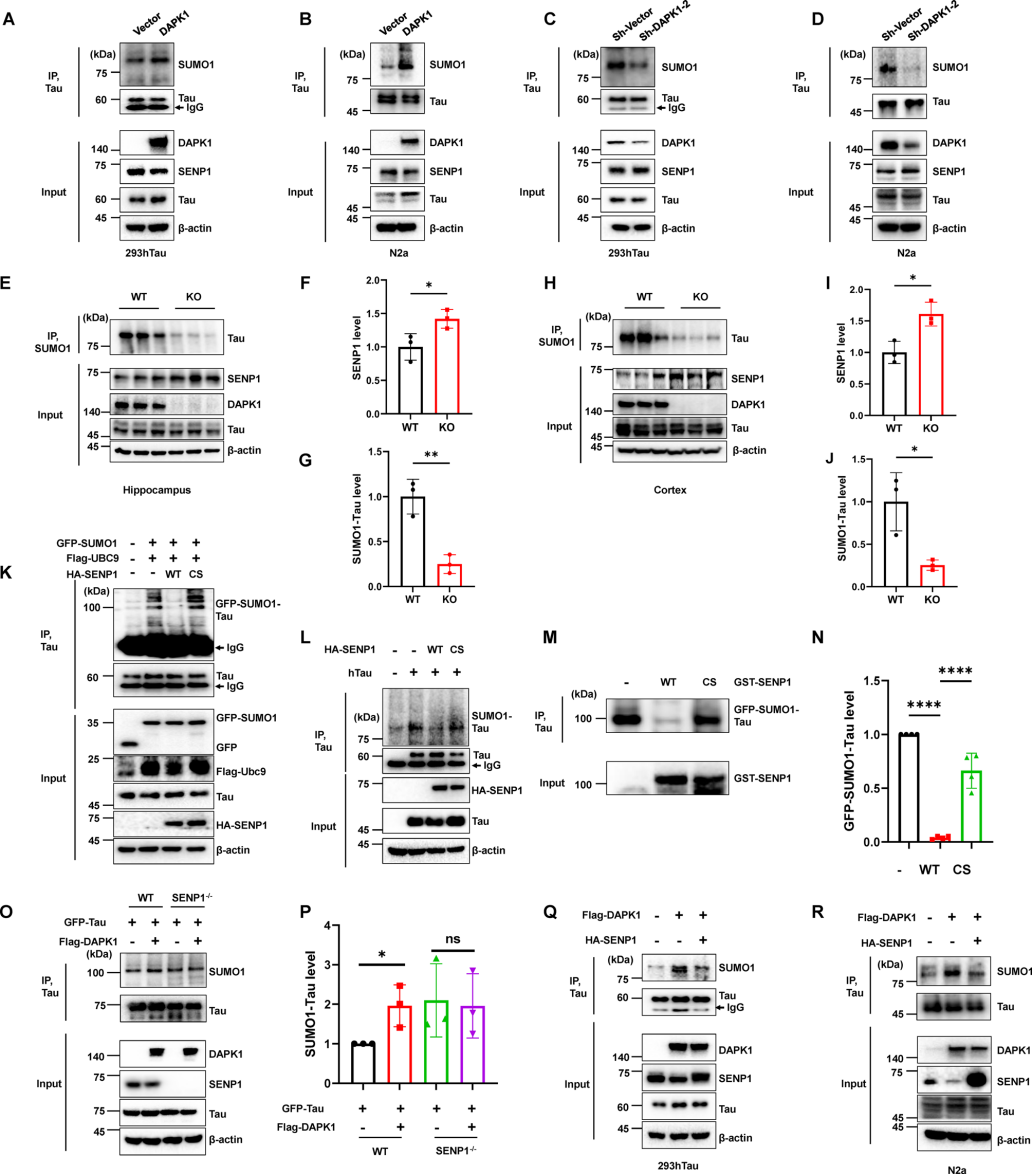
DAPK1 induces tau SUMOylation by regulating SENP1 **(A-B)** 293hTau (**A**) and N2a (**B**) cells were transfected with vector or Flag-DAPK, and cell lysates were subjected to immunoprecipitation with an anti-tau antibody. Immunoblotting analysis was performed using indicated antibodies to evaluate tau SUMOylation. **(C-D)** 293hTau (**C**) and N2a (**D**) cells were transfected with sh-vector or sh-DAPK1, and cell lysates were subjected to immunoprecipitation with an anti-tau antibody. Immunoblotting analysis was performed using indicated antibodies to evaluate tau SUMOylation. **(E-J)** The hippocampal (**E**-**G**) and cortical (**H**-**J**) tissues from 6-month-old WT or DAPK1-KO mice were homogenized and subjected to immunoprecipitation using an anti-SUMO1 antibody, followed by immunoblotting analysis using indicated antibodies to detect SENP1 level and tau SUMOylation. *n* = 3 mice/group. **(K)** WT or the inactive (Cys603 to Ser, CS) SENP1 was expressed with GFP-SUMO1 and Flag-Ubc9 in 293hTau cells. Cell lysates were immunoprecipitated with an anti-tau antibody. Tau SUMOylation was detected using an anti-GFP antibody. The input samples were detected using indicated antibodies. **(L)** Human tau was expressed in HEK-293T cells with WT or the CS SENP1. Cell lysates were immunoprecipitated with an anti-tau antibody. Tau SUMOylation was detected using an anti-SUMO1 antibody. The input samples were detected using indicated antibodies. **(M-N)** SUMOylated tau was purified from 293hTau cells expressing GFP-SUMO1 and equally divided into three parts. Purified proteins were incubated with recombinant GST-SENP1 or the CS mutant in reaction buffer at 37 °C for 20 min. Samples were immunoprecipitated using an anti-tau antibody, and tau SUMOylation was probed using an anti-SUMO1 antibody. The quantification of GFP-SUMO1-conjugated tau was shown in N. **(O-P)** GFP-tau was co-transfected with or without Flag-DAPK1 in WT or SENP1 knockout (SENP1^−/−^) cells. Cell lysates were subjected to immunoprecipitation with an anti-tau antibody, followed by immunoblotting analysis using anti-tau or SUMO1 antibodies. The input samples were detected using indicated antibodies. The quantification of SUMOylated tau was shown in P. **(Q-R)** HA-SENP1 was reintroduced into 293hTau (Q) and N2a (R) cells expressing Flag-DAPK1. Cell lysates were subjected to immunoprecipitation with an anti-tau antibody, followed by immunoblotting analysis using anti-tau or SUMO1 antibody. The input samples were detected using indicated antibodies. Representative images from triplicate repeats are shown. *p < 0.05, **p < 0.01, ****p < 0.0001, ns, not significant. Two-tailed unpaired *t*-test was used F, G, I, J and P. One-way ANOVA followed by Tukey’s *post-hoc* test was used in N

Tau SUMOylation plays an essential role in regulating tau proteostasis through affecting its ubiquitination and phosphorylation [7]. A recent research reported that SENP1 reduces the level of SUMO1-conjugated tau [50]. We previously found that DAPK1 modulates both tau phosphorylation and its protein stability through a kinase activity-dependent manner [14]. Since DAPK1 is capable of regulating SENP1 expression by phosphorylating Ser126, we reasoned that DAPK1 can regulate tau SUMOylation through SENP1. We first found that overexpressing DAPK1 in 293hTau and N2a cells promoted tau SUMOylation, as indicated by an increase in the intensity of SUMO1 signals larger than 75 kDa (Fig. 4A, B). However, DAPK1 knockdown in these cell lines led to a reduction in SUMOylated tau levels (Fig. 4C, D). Endogenous SENP1 expression was simultaneously altered following DAPK1 overexpression or knockdown, which is consistent with our previous results (Fig. 2). We also examined tau SUMOylation and SENP1 expression in brains of DAPK1-KO mice, and found that the SUMOylation of tau was significantly reduced while the SENP1 protein level was elevated in the hippocampus (Figs. 4E-G and S6A) and cortex (Figs. 4H-J and S6B) of DAPK1-KO mice. However, the mRNA level of SENP1 was similar between WT and DAPK1-KO mice (Fig. S6C, D). Next, we performed cell SUMOylation assays to confirm whether SENP1 deSUMOylates tau. 293hTau cells were transfected with GFP-SUMO1 and Flag-Ubc9, an E2 enzyme that enhances SUMOylation. Cells without SENP1 transfection exhibited obvious tau SUMOylation at ~ 100 kDa, which was diminished after the overexpression of WT SENP1 (Fig. 4K). However, the inactive SENP1 mutant C603S failed to do so (Fig. 4K). SENP1 also had a similar effect on exogenous tau SUMOylation (Fig. 4L). Moreover, the in vitro deSUMOylation assay showed that WT SENP1 but not SENP1 C603S efficiently removed SUMO1 from SUMOylated tau proteins derived from 293hTau cells (Fig. 4M, N). These results support tau as a direct target of SENP1.

We further investigated effects of SENP1-mediated tau deSUMOylation on tau phosphorylation and ubiquitination in vitro. GFP-SUMO1 and Flag-Ubc9 overexpression remarkably increased levels of SUMOylated tau and pT231-Tau (∼100 kDa) in cells (Fig. S7A-C). However, WT SENP1 but not SENP1 C603S significantly reduced SUMOylated tau and pT231-Tau levels (Fig. S7A-C). These data showed that SENP1 can inhibit tau SUMOylation and tau phosphorylation. In addition, since tau SUMOylation has been shown to inhibit ubiquitination-dependent tau degradation, we sought to explore whether SENP1 can facilitate the ubiquitination of tau by promoting its deSUMOylation. We observed that WT SENP1 but not SENP1 C603S promoted the ubiquitination of tau (Fig. S7D, E), which was correlated with the changes in tau SUMOylation induced by SENP1. Moreover, SENP1 was highly colocalized with tau in the cytoplasm (Fig. S7F, G). We generated a SENP1 knockout (SENP1^−/−^) cell line using the CRISPR/Cas9 gene editing (Fig. S8). SENP1 ablation abolished the effect of DAPK1 on tau SUMOylation (Fig. 4O, P), whereas restoring SENP1 levels in DAPK1-overexpressing cells counteracted the DAPK1-induced accumulation of SUMOylated tau (Fig. 4Q, R). Overall, these findings indicate that DAPK1 promotes tau SUMOylation by degrading SENP1, highlighting the critical role of DAPK1-SENP1 axis in tau SUMOylation and proteostasis.

### DAPK1 KO attenuates tau SUMOylation and aberrant phosphorylation in vitro and in vivo via regulating SENP1 expression

**Fig. 5:**
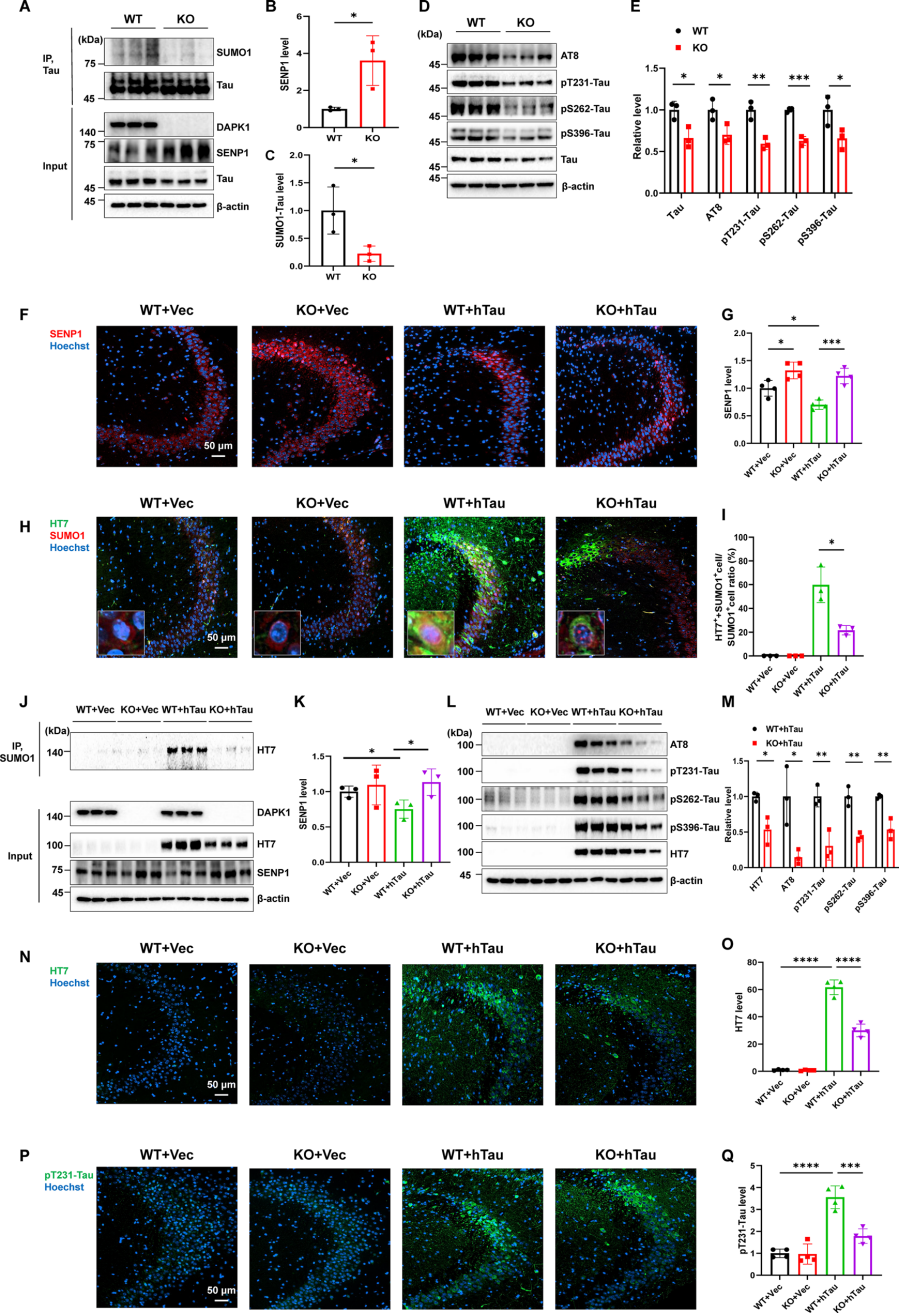
DAPK1 KO attenuates tau SUMOylation and aberrant phosphorylation in vitro and in vivo by regulating SENP1 expression. **(A-C)** Cell lysates from WT or KO primary neurons were subjected to immunoprecipitation using an anti-tau antibody, followed by immunoblotting analysis using anti-tau or SUMO1 antibody. The input samples were detected using indicated antibodies. *n* = 3 samples/group. **(D-E)** Levels of total tau and phosphorylated tau at Ser202/Thr205 (AT8), Thr231 (pT231), Ser262 (pS262), Ser396 (pS396) were detected by immunoblotting analysis using indicated antibodies in WT and KO primary neurons. The tau and pTau levels were quantified by determining the ratios to β-actin, then normalized to those of the WT group. *n* = 3 samples/group. **(F-G)** Representative immunostaining of SENP1 in the hippocampal CA3 area in WT or DAPK1-KO mice infused with vector or hTau virus. Samples were collected 10 weeks post virus injection. Hoechst 33342 was used to stain cell nucleus. Scale bar = 50 μm. *n* = 4 mice/group. **(H-I)** Immunostaining of human tau and SUMO1 by HT7 and anti-SUMO1 antibodies, respectively, in the hippocampal CA3 area in WT or DAPK1-KO mice infused with vector or hTau virus. Insert shows representative staining pattern for SUMO1 (red) and HT7 (green) signals. The ratio of HT7 and SUMO1 positive (HT7^+^+SUMO1^+^) cells to total SUMO1 positive (SUMO1^+^) cells were determined. Scale bar = 50 μm. *n* = 3 mice/group. **(J-K)** Mouse hippocampal lysates were subjected to immunoprecipitation using an anti-SUMO1 antibody, followed by immunoblotting analysis using the HT7 antibody to measure tau SUMOylation. The expression of DAPK1 and SENP1 was detected using indicated antibodies. The quantification of SENP1 was presented in K. *n* = 3 mice/group. **(L-M)** Total tau and tau phosphorylation at AT8, pT231, pS262, and pS396 in the hippocampus were detected by immunoblotting analysis using indicated antibodies in WT and DAPK1-KO mice injected with vector or hTau virus. The tau and pTau levels were quantified by determining the ratios to β-actin, then normalized to those of the WT + hTau group. *n* = 3 mice/group. **(N-Q)** Immunostaining of total human tau and tau phosphorylation at Thr231 using HT7 and anti-pT231 antibodies, respectively, in the hippocampal CA3 area in WT or DAPK1-KO mice infused with vector or hTau virus. Scale bar = 50 μm. *n* = 4 mice/group. Representative images from triplicate repeats are shown. *p < 0.05, **p < 0.01, ***p < 0.001, ****p < 0.0001. Two-tailed unpaired *t*-test was used in B, C, E, I, K and M. One-way ANOVA followed by Tukey’s *post-hoc* test was used in G, O and Q

To elucidate the pathological relevance of DAPK1 dysregulation-induced tau SUMOylation, we first analyzed tau SUMOylation and phosphorylation levels in primary neurons from DAPK1-KO mice. Consistent with the effect of DAPK1 knockdown in cell lines, DAPK1 KO significantly increased SENP1 protein level (Fig. 5A, B) while reducing SUMOylated tau contents (Fig. 5A, C). Since tau SUMOylation increases tau phosphorylation and accumulation [7], we examined total tau as well as the phosphorylation of tau at AD-related sites including AT8 (Ser202/Thr205), Thr231 (pT231-Tau), Ser262 (pS262-Tau), and Ser396 (pS396-Tau), and found that both total tau and phosphorylated tau at all sites were lower in DAPK1-KO neurons than in WT neurons (Fig. 5D, E). These findings suggest that DAPK1 ablation can inhibit tau SUMOylation, thus leading to reduced tau phosphorylation and accumulation in neurons.

To further investigate whether DAPK1 affects tau phosphorylation and accumulation by modulating tau SUMOylation in vivo, we developed a mouse model of tauopathy by stereotactic injection of rAAV expressing hTau-mCherry into the CA3 region of the hippocampus to mimic the pathological accumulation of tau in the brain. The model is known to develop tau hyperphosphorylation, synaptic dysfunction, oxidative damage and gliosis, which are important pathologies reflective of early stage tauopathy [36, 52]. We verified hTau-mCherry expression in CA3 by detecting mCherry signals using immunofluorescence and measuring hTau-mCherry protein levels using an antibody (HT7) targeting human tau (Fig. S9A-C). hTau overexpression by virus infusion did not affect DAPK1 protein expression in the hippocampus, we then measured the activity of endogenous DAPK1 by checking the phosphorylation of MLC at Ser19 (pMLC). The results showed that the pMLC level was elevated in the CA3 area in WT + hTau group compared with that in WT + Vec mice (Figs. 5J and S9D, E). However, DAPK1 KO normalized MLC phosphorylation triggered by hTau overexpression (Fig. S9D, E). Intriguingly, the pMLC levels in WT and DAPK1-KO mice injected with vector virus were not statistically different (Fig. S9D, E), which might be explained by the relatively low catalytic activity of DAPK1 toward MLC at basal state [53], and the compensation effects induced by other MLC kinases [54]. We further detected an obvious decrease in SENP1 level in WT + hTau group compared with WT + Vec group, and this change was reversed in DAPK1-KO + hTau group (Fig. 5F, G, J and K). SENP1 is predominantly expressed in neurons in the hippocampus, as it was strongly colocalized with the neuronal cell marker NeuN, but not with the glial cell markers GFAP and IBA1 (Fig. S9F, G). To investigate the SUMOylation of hTau in each group, we performed coimmunostaining analysis using HT7 and SUMO1-specific antibodies. The immunostaining analysis revealed that both SUMO1 and tau signals are diffusely distributed in neuronal cytoplasm in the CA3 area of mice infused with hTau virus (Fig. 5H). We observed a high number of cells containing both HT7-positive signals and SUMO1 signals in the WT + hTau group, but the ratio of HT7 and SUMO1 dual positive cells to SUMO1 positive cells was reduced in KO + hTau group (Fig. 5H, I). The decline in the colocalization of HT7 and SUMO1 signals indicates that tau SUMOylation in the hippocampus might be reduced after DAPK1 ablation. Moreover, immunoprecipitation with an anti-SUMO1 antibody confirmed robust attenuation of tau SUMOylation in the hippocampus of DAPK1-KO + hTau mice compared with WT + hTau mice (Fig. 5J). To characterize whether DAPK1 KO can alleviate tau pathology in the hippocampus, we examined levels of total hTau and phosphorylated hTau at multiple AD-related sites. Immunoblotting analysis showed that levels of total hTau as well as tau phosphorylation at Ser202/Thr205 (AT8), T231, S262, and S396 residues were decreased in the hippocampus of DAPK1-KO + hTau mice compared with those in WT + hTau mice (Fig. 5L, M). Immunofluorescence revealed that the HT7 signal intensity and pT231-Tau accumulation were significantly lower in the DAPK1-KO + hTau mice than in WT + hTau mice (Fig. 5N-Q). In summary, we found that hTau overexpression increased DAPK1 activity and subsequently decreased SENP1 levels, leading to dysregulated tau SUMOylation and phosphorylation in neurons. However, genetic ablation of DAPK1 effectively prevented aberrant SUMOylation-induced accumulation of total and phosphorylated tau in the hippocampus.

### DAPK1 KO attenuates abnormal tau accumulation-induced synaptic dysfunction and gliosis in hTau mice

**Fig. 6:**
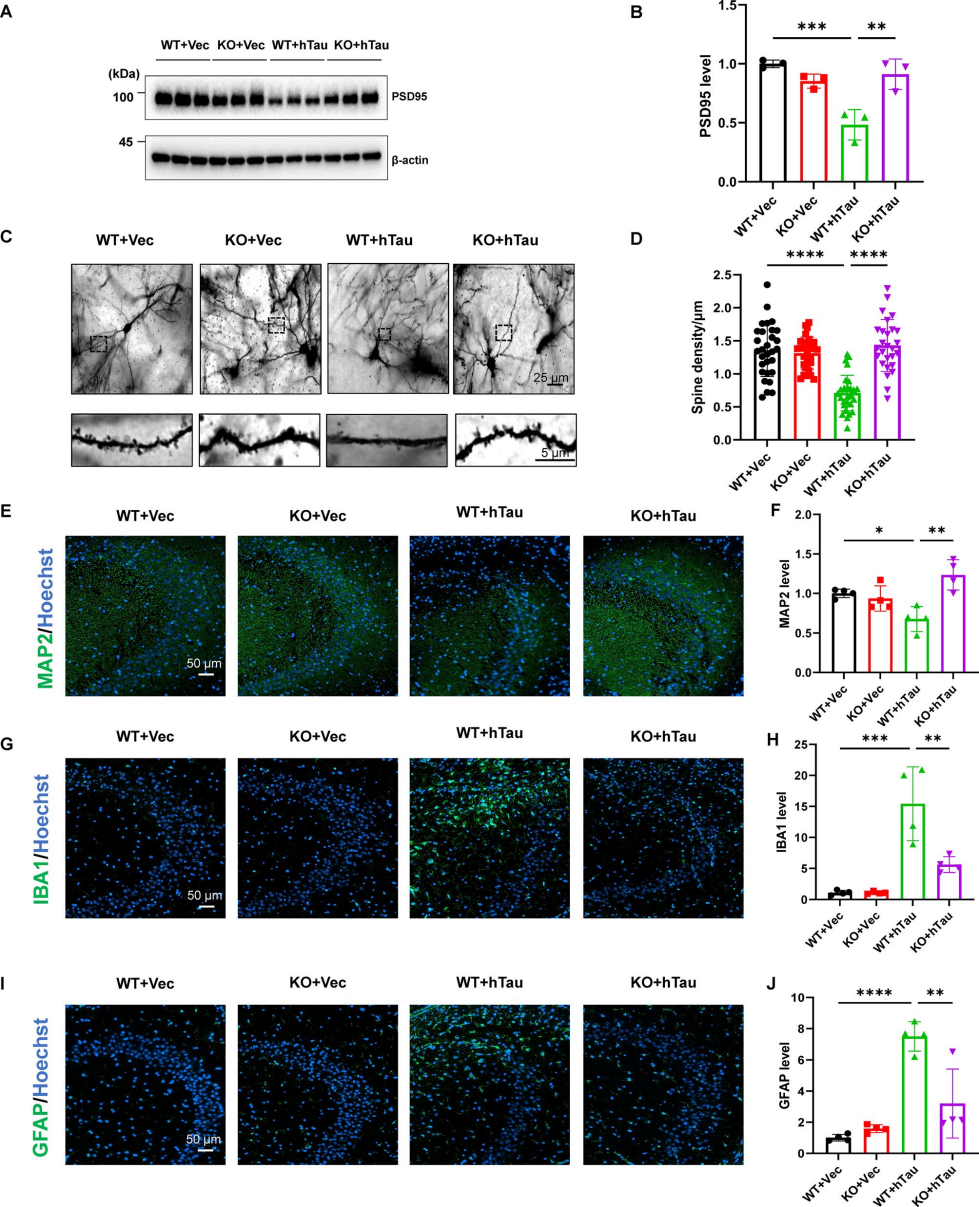
DAPK1 KO attenuates abnormal tau accumulation-induced synaptic dysfunction and neuroinflammation in the hippocampus. **(A-B)** Mouse hippocampal lysates were subjected to immunoblotting analysis for PSD95. *n* = 3 mice/group. **(C-D)** Golgi-Cox staining was conducted to analyze the dendritic spine density in hippocampal CA3 area in WT and DAPK1-KO mice injected with vector or hTau virus. 27 to 30 randomly selected dendrites in CA3 area were selected from each group to quantify the dendritic spine density, and representative images for neurons (scale bar = 25 μm) and selected dendrites (scale bar = 5 μm) are shown. *n* = 3 mice/group. **(E-F)** Representative immunostaining of MAP2 in hippocampal CA3 area in WT and DAPK1-KO mice injected with vector or hTau virus. Scale bar = 50 μm. *n* = 4 mice/group. **(G-J)** Representative immunostaining of IBA1 and GFAP in hippocampal CA3 area in WT and DAPK1-KO mice injected with vector or hTau virus. Scale bar = 50 μm. *n* = 4 mice/group. Representative images from triplicate repeats are shown. *p < 0.05, **p < 0.01, ***p < 0.001, ****p < 0.0001. One-way ANOVA followed by Tukey’s *post-hoc* test was used for analysis

Mounting evidence suggests that abnormal tau accumulation disrupts synaptic integrity by impairing postsynaptic density organization and dendritic spine dynamics [36, 55]. We measured levels of key synaptic markers and the dendritic spine density in WT and DAPK1-KO mice after virus injection. Immunoblotting analysis revealed that hTau overexpression downregulated the expression of postsynaptic density protein-95 (PSD95) in the hippocampus of WT mice, whereas this downregulation was fully reversed by DAPK1 ablation (Fig. 6A, B). Since PSD95 is highly enriched in dendritic spines that are essential for synaptic transmission, we investigated the dendritic spine density within the hippocampus. Compared with the WT + Vec group, the WT + hTau group presented a significantly reduced dendritic spine density (Fig. 6C, D), indicating synaptic impairment following hTau overexpression. Notably, the loss of dendritic spines was ameliorated in DAPK1-KO + hTau mice (Fig. 6C, D). Similarly, the expression of microtubule-associated protein 2 (MAP2) in the hippocampus was restored to normal levels in DAPK1-KO mice but not in WT mice after hTau overexpression (Fig. 6E, F). Collectively, these findings demonstrate that DAPK1 ablation alleviates synaptic dysfunction associated with tau accumulation by normalizing PSD95 expression and preserving dendritic spine integrity.

In addition to synaptic damage, the aberrant accumulation of tau is associated with glial activation and neuroinflammatory responses [56]. We examined the expression of ionized calcium-binding adaptor molecule 1 (IBA1) and glial fibrillary acidic protein (GFAP), which are markers of reactive microglia and astrocytes, respectively. Our findings indicated that WT + hTau mice had higher expression of both IBA1 (Fig. 6G, H) and GFAP (Fig. 6I, J) signals in the hippocampal CA3 region than the control mice, which is consistent with our previous report [36]. However, DAPK1 KO was able to suppress the activation of microglia and astrocytes induced by tau accumulation, as indicated by the normalization of IBA1 and GFAP expression in CA3 regions of the DAPK1-KO + hTau group (Fig. 6G-J). Thus, DAPK1 KO is sufficient to ameliorate abnormal glial cell activation induced by tau accumulation in the hippocampus.

### DAPK1 KO mitigates cognitive impairments induced by hippocampal tau overexpression

**Fig. 7:**
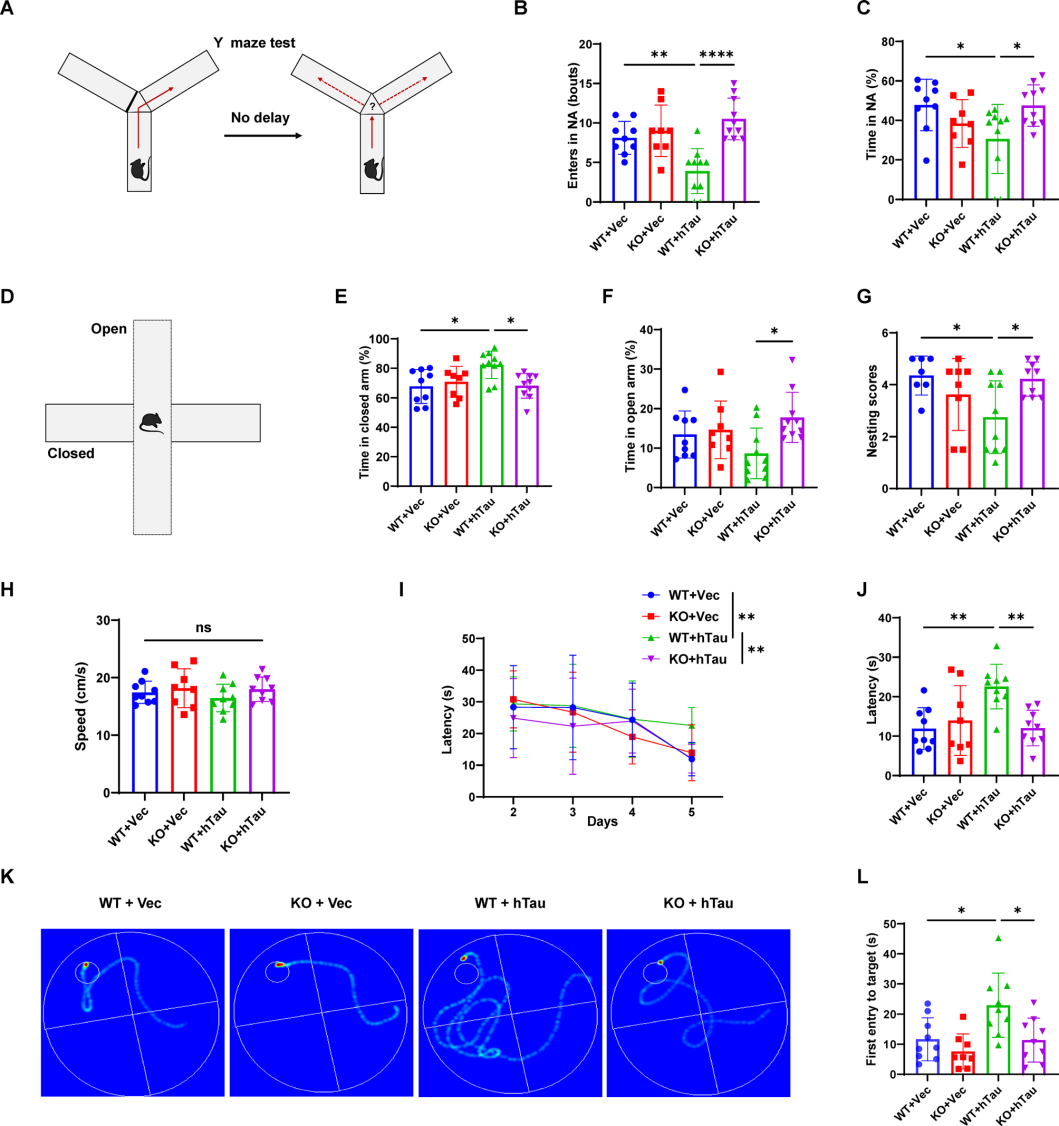
DAPK1 KO mitigates emotional and cognitive impairments in a tauopathy mouse model. **(A-C)** Y-maze test was performed to measure the short-term memory ability. The number of NA entry and the percentage of time spent in NA for each group in the Y-maze were compared. *n* = 8–10 mice/group. **(D-F)** Elevated plus maze test was conducted to evaluate the anxiety-like phenotype. The percentage of time spent in closed arms (solid line) and open arms (dotted line) were determined for each group. *n* = 8–10 mice/group. **(G)** A nest building test was used to compare the cognitive ability in each group. The nesting score was evaluated. *n* = 7–10 mice/group. **(H-L)** Mice were subjected to the MWM test to assess spatial learning and memory abilities. The mean swimming speed (H) at day one, the escape latency in the training phase (I), the escape latency (J) and the representative swim trajectories on day five (K) are shown. The time of first entry to the platform position on day six (L) in the probe trial is presented. *n* = 8–9 mice/group. Data are expressed as mean ± SD, *p < 0.05, **p < 0.01, ****p < 0.0001. One-way ANOVA followed by Tukey’s *post-hoc* test was used for analysis

The recovery of synaptic markers and normalization of neuronal tau levels in DAPK1-KO mice injected with hTau virus indicate a neuroprotective effect of DAPK1 depletion in vivo. We performed a series of behavioral experiments to evaluate the emotional and cognitive functions (Fig. S9A). In Y-maze test, the number of entries into and the time spent in the NA were lower in the WT + hTau group than in the WT + Vec group (Fig. 7A-C), suggesting short-term spatial memory deficits in the WT + hTau group. However, compared with the WT + hTau group, the DAPK1-KO + hTau group presented an increased number of entries into and time spent in NA, indicating that DAPK1 KO can restore short-term spatial memory (Fig. 7A-C). Besides, hTau overexpression-induced anxiety-like behavior was measured by the EPM test (Fig. 7D-F). Mice in the WT + hTau group spent more time in closed arm than those in WT + Vec group, indicating the induction of an anxiety-like phenotype (Fig. 7E). However, DAPK1 ablation alleviated anxiety-like behavior as evidenced by an increase in the time spent in open arm for the DAPK1-KO + hTau mice (Fig. 7F). Nest-building behavior, a validated indicator of cognitive integrity in mouse [57], was impaired in hTau-overexpressing mice (Fig. 7G). However, this deficit was reversed in DAPK1-KO + hTau group. We also conducted the MWM test to further evaluate spatial learning and memory. All experimental groups exhibited similar swimming speeds (Fig. 7H). During the training phase, all mice showed a progressive decrease in escape latency over time (Fig. 7I). The escape latency of the WT + hTau group was significantly longer than that of the WT + Vec group on day five, indicating that hTau overexpression resulted in cognitive impairments (Fig. 7I-K). Conversely, the escape latency of the DAPK1-KO + hTau group was comparable to that of the WT + Vec group and was much shorter than that of the WT + hTau group on day five (Fig. 7I-K). Additionally, the WT + hTau group exhibited a significantly longer latency to reach the platform location than the WT + Vec group did in the probe trial (Fig. 7L). In contrast, the DAPK1-KO + hTau mice presented a reduced latency to locate target area (Fig. 7L), suggesting recovery of spatial learning and memory ability. These findings collectively demonstrate that genetic DAPK1 ablation can mitigate hTau overexpression-induced cognitive and emotional impairments.

### Pharmacological Inhibition of DAPK1 attenuates tau SUMOylation and aberrant phosphorylation in vitro and in vivo

**Fig. 8:**
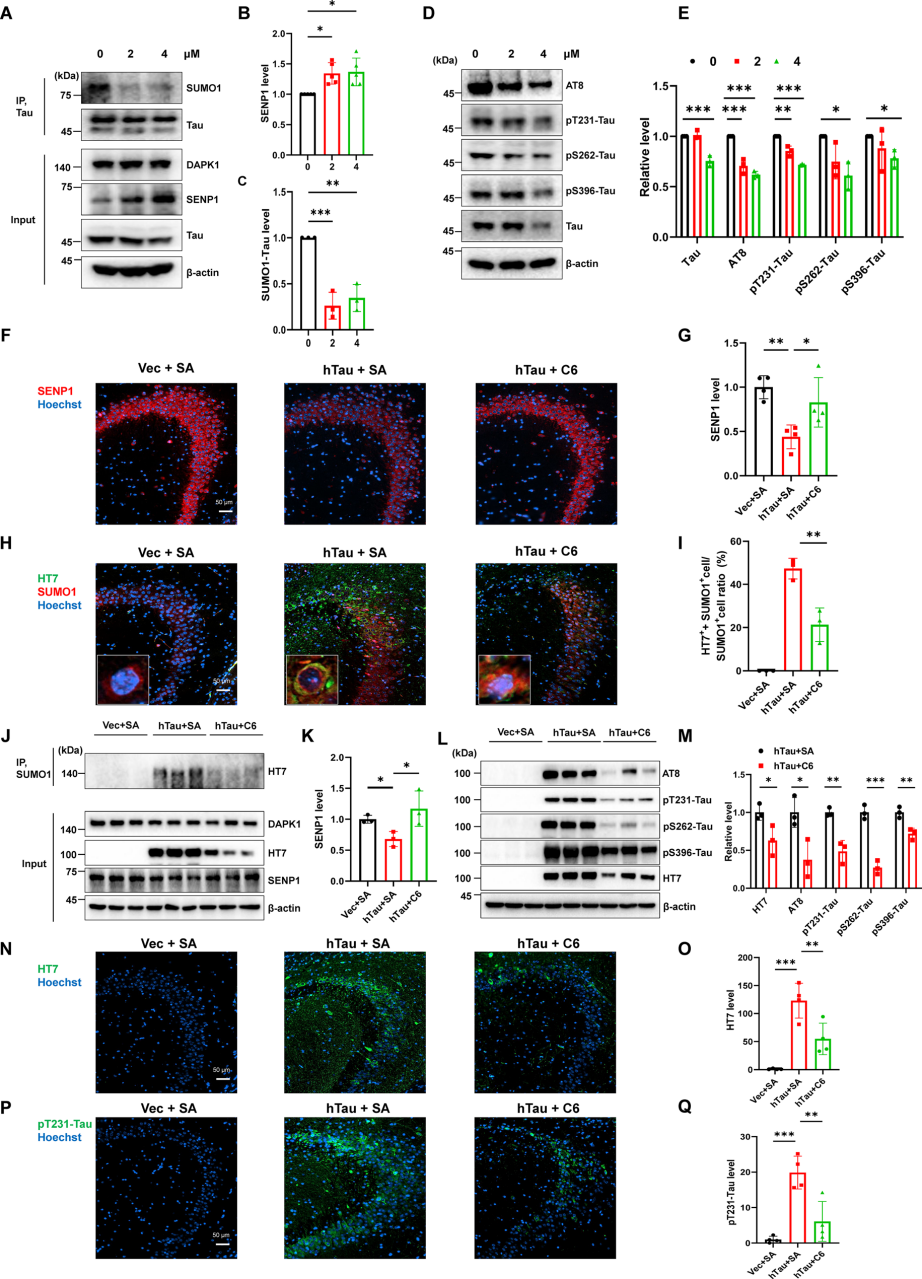
Inhibition of DAPK1 by C6 rescues tau pathologies in vitro and in vivo by modulating SENP1 expression. **(A-C)** Primary neurons from WT mice were treated with different concentrations of C6 for 24 h. Cell lysates were subjected to immunoprecipitation using an anti-tau antibody, followed by immunoblotting analysis using anti-SUMO1 antibody. Input samples were analyzed using indicated antibodies. **(D-E)** Total tau and tau phosphorylation at AT8, pT231, pS262, and pS396 in the WT primary neuron treated with or without C6 were determined by immunoblotting analysis using indicated antibodies. The tau and pTau levels were quantified by determining the ratios to β-actin, then normalized to those of the 0 µM group. **(F-G)** Representative immunostaining of SENP1 in the hippocampal CA3 area in mouse models treated with or without C6 for 10 weeks. Hoechst 33342 was used to stain cell nucleus. Scale bar = 50 μm. *n* = 4 mice/group. **(H-I)** Immunostaining of human tau and SUMO1 by HT7 and anti-SUMO1 antibodies, respectively, in the hippocampal CA3 area in mouse models treated with or without C6. Insert shows representative staining pattern for SUMO1 (red) and HT7 (green) signals. The ratio of HT7 and SUMO1 positive (HT7^+^+SUMO1^+^) cells to total SUMO1 positive (SUMO1^+^) cells were determined. Scale bar = 50 μm. *n* = 3 mice/group. **(J-K)** Mouse hippocampal lysates were subjected to immunoprecipitation using an anti-SUMO1 antibody, followed by immunoblotting analysis using the HT7 antibody to measure tau SUMOylation. The expression of DAPK1 and SENP1 was detected using indicated antibodies. The quantification of SENP1 was presented in K. *n* = 3 mice/group. **(L-M)** Total tau and tau phosphorylation at AT8, pT231, pS262, and pS396 in the hippocampus were detected by immunoblotting analysis using indicated antibodies in mouse models treated with or without C6. The tau and pTau levels were quantified by determining the ratios to β-actin, then normalized to those of the hTau + SA group. *n* = 3 mice/group. **(N-Q)** Immunostaining of total human tau and tau phosphorylation at Thr231 using HT7 and anti-pT231 antibodies, respectively, in in mouse models treated with or without C6. Scale bar = 50 μm. *n* = 4 mice/group. Representative images from triplicate repeats are shown. *p < 0.05, **p < 0.01, ***p < 0.001. Two-tailed unpaired *t*-test was used in I, K and M. One-way ANOVA followed by Tukey’s *post-hoc* test was used in B, C, E, G, O and Q

To evaluate the therapeutic potential of targeting DAPK1 to regulate tau SUMOylation in AD-like pathologies, we investigated the effects of pharmacological inhibition of DAPK1 on tau pathology, synaptic plasticity, and cognitive function. We utilized the compound C6 (4-(pyridin-3-methylene) oxazole-5(4 H)-one), a selective and potent DAPK1 inhibitor (IC_50_ = 69 nM) which has been characterized to suppress its catalytic activity in vitro and in vivo. We and others previously demonstrated that C6-induced DAPK1 inhibition effectively ameliorate ischemic brain injury and epileptic seizure in mouse models [58–61]. Primary neurons were treated with 2 or 4 µM C6 for 24 h in vitro. C6 at 2 µM prevented tau SUMOylation and elevated SENP1 levels without altering total tau (Fig. 8A-C). A higher dose of C6 at 4 µM further reduced total tau level in primary neurons (Fig. 8A-C). In line with the decrease in SUMOylated tau, DAPK1 inhibition also significantly suppressed tau phosphorylation at multiple AD-related sites, including AT8, pS262, pS396, and pT231 (Fig. 8D, E). We then analyzed the effect of DAPK1 inhibitor on hTau mouse models by intraperitoneal injection of either C6 (10 mg/kg, three times weekly) or saline (SA) for 9 weeks (Fig. S10A). All mice showed an identical increase in body weight during the treatment period (Fig. S10B). We confirmed the inhibitory efficacy of C6 by measuring pMLC levels in the hippocampus of mice treated with C6 (Fig. S10C, D). Consistent with the effect of DAPK1 depletion, C6 treatment not only restored SENP1 expression (Fig. 8F, G, J and K), but also effectively diminished tau SUMOylation in the hippocampus (Fig. 8H-J). In addition to the downregulation of tau SUMOylation by C6, the accumulation and aberrant phosphorylation of hTau in the CA3 region were robustly reduced in C6-treated group compared with the saline group (Fig. 8L-Q). These findings collectively demonstrate that DAPK1 inhibition reduces tau SUMOylation, subsequently diminishing both total and phosphorylated tau accumulation in the hippocampus.

### DAPK1 inhibitor attenuates abnormal tau accumulation-induced synaptic dysfunction and gliosis in the hippocampus

**Fig. 9:**
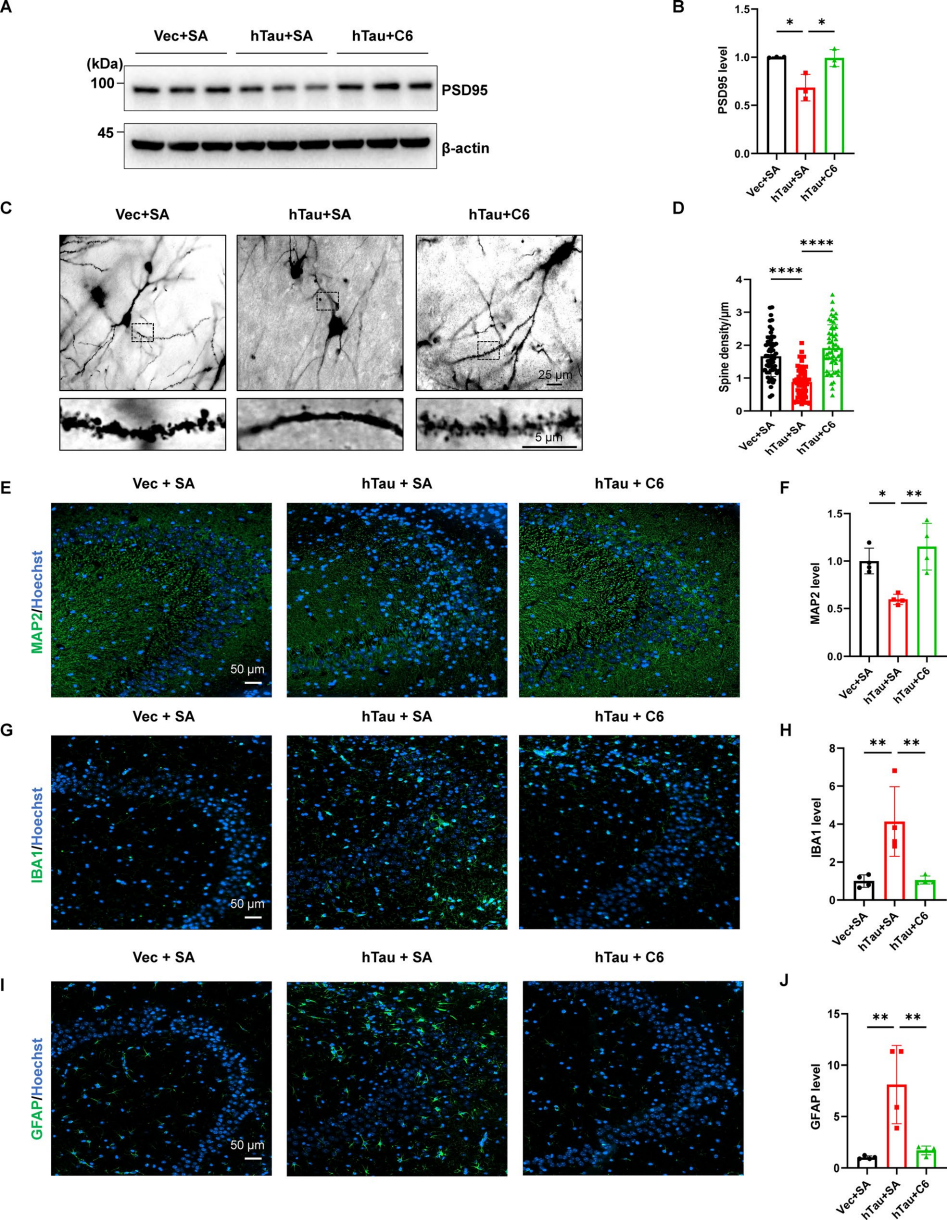
DAPK1 inhibition attenuates abnormal tau accumulation-induced synaptic dysfunction and neuroinflammation in the hippocampus. **(A-B)** The hippocampal lysates from mice treated with or without C6 were subjected to immunoblotting analysis for PSD95. *n* = 3 mice/group. **(C-D)** Representative images of Golgi-Cox staining in hippocampal CA3 area in mice treated with or without C6. 54 to 61 randomly selected dendrites in CA3 area from each group were selected to quantify the dendritic spine density. Representative images for neurons (scale bar = 25 μm) and selected dendrites (scale bar = 5 μm) are presented. *n* = 3 mice/group. **(E-F)** Representative immunostaining of MAP2 in hippocampal CA3 area in mice treated with or without C6. Scale bar = 50 μm. *n* = 4 mice/group. **(G-J)** Representative immunostaining of IBA1 and GFAP in hippocampal CA3 area in mice treated with or without C6. Scale bar = 50 μm. *n* = 4 mice/group. Representative images from triplicate repeats are shown. *p < 0.05, **p < 0.01, ****p < 0.0001. One-way ANOVA followed by Tukey’s *post-hoc* test was used for analysis

We then investigated whether DAPK1 inhibition could ameliorate hTau-induced synaptic dysfunction and gliosis. Similar to the results obtained using DAPK1-KO mice, treatment with the DAPK1 inhibitor C6 reversed the downregulation of hippocampal PSD95 caused by hTau accumulation (Fig. 9A, B). C6 administration also significantly reversed the decline in dendritic spine density in the CA3 region of hTau mice (Fig. 9C, D). Moreover, hTau overexpression-induced decrease in MAP2 level was restored after C6 administration (Fig. 9E, F). Consistent with our previous data, inhibition of DAPK1 attenuated hTau accumulation-induced activation of astrocytes and microglia in the CA3 region (Fig. 9G-J). Thus, inactivation of DAPK1 is sufficient to protect the brain against hTau accumulation-triggered synaptic impairment and gliosis.

### Inhibition of DAPK1 ameliorates cognitive impairment in a mouse model of tauopathy

**Fig. 10:**
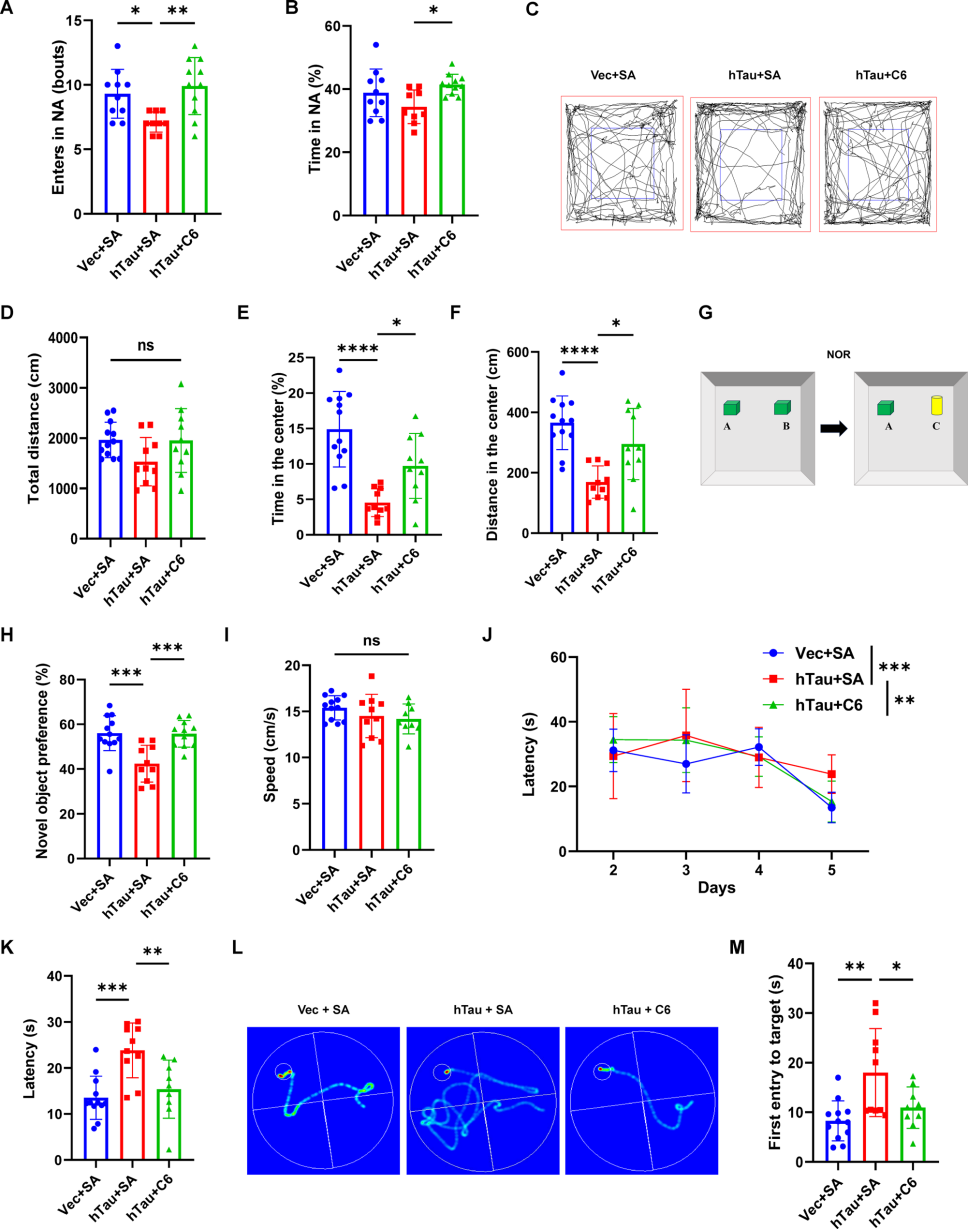
DAPK1 inhibition ameliorates emotional and cognitive impairments in a mouse model of tauopathy. (**A-B)** The Y-maze test was applied to evaluate the short-term memory ability in mice treated with or without C6. The number of NA entry and the percentage of time spent in NA for each group are presented. *n* = 9–11 mice/group. **(C-F)** The open field test (OFT) was employed to analyze the anxiety-like behavior in mice treated with or without C6. Representative movement trajectories (**C**) for each group are shown. The blue square indicates the center area in OFT. The total ambulatory distance (**D**), the percentage of time spent (**E**) and distance traveled (**F**) in the center area are presented. *n* = 10–12 mice/group. **(G-H)** A novel object recognition (NOR) test (**G**) was performed to evaluate the preference toward novel object (**H**) in mice treated with or without C6. *n* = 10–12 mice/group. **(I-M)** Mice were subjected to the MWM test to assess spatial learning and memory abilities. The mean swimming speed (**I**) at day one, the escape latency in the training phase (**J**), the escape latency (**K**) and the representative swim trajectories on day five (**L**) are shown. The time of first entry to the platform position on day six (**M**) in the probe trial is presented. *n* = 9–12 mice/group. Data are expressed as mean ± SD, *p < 0.05, **p < 0.01, ***p < 0.001, ****p < 0.001, ns, not significant. One-way ANOVA was followed by Tukey’s *post-hoc* test was used for analysis

To investigate whether DAPK1 inhibition can rescue hTau-induced memory and emotional deficits, we conducted a series of behavioral analyses (Fig. S10A). Compared with the hTau + SA group, the hTau + C6 group presented significant improvements in short-term spatial memory, as evidenced by increase in the number of entries into and time spent in the NA in Y-maze test (Fig. 10A, B). Next, we measured the locomotor and anxiety-like behavior of mice using the OFT. The distance traveled and the time spent in the central region were significantly less in hTau + SA mice than in Vec + SA mice (Fig. 10C-F), suggesting that hTau overexpression induced an anxiety-like phenotype. However, the distance traveled in the central zone was increased in hTau + C6 group (Fig. 10C-F), suggesting that DAPK1 inhibition attenuated hTau-induced anxiety-like behavior. Moreover, C6 treatment improved cognitive performance in the NOR test, as reflected by an increased preference for the novel object in hTau + C6 group (Fig. 10G, H). In the MWM test, all groups displayed comparable swimming speeds in the training phase (Fig. 10I). C6-treated hTau mice exhibited significantly shorter escape latencies to locate the platform than did mice in the vehicle group (Fig. 10J-L). In the probe trial, C6-treated hTau mice also spent less time to reach the platform location (Fig. 10M). Collectively, these data demonstrate that pharmacological inhibition of DAPK1 effectively mitigates hTau accumulation-induced cognitive and emotional impairments. These results are consistent with what we observed in DAPK1-KO mice and provide solid evidence for the future evaluation of DAPK1 as a therapeutic target for AD.

### SENP1 levels are significantly reduced in postmortem brain tissues from AD patients and are negatively correlated with DAPK1 expression

**Fig. 11:**
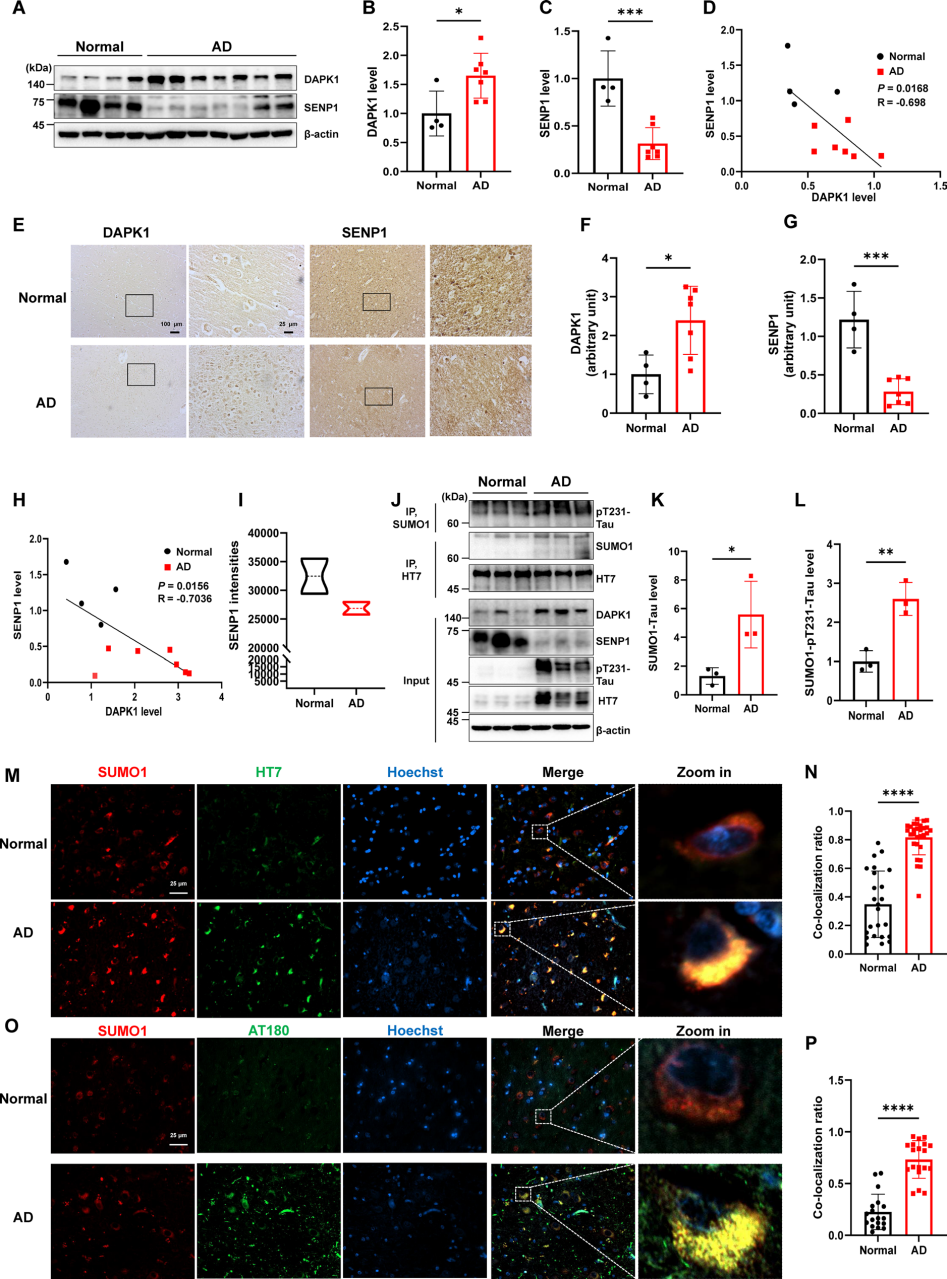
SENP1 level is inversely correlated with the expression of DAPK1 in brains of human AD patients. (**A-D)** Quantification of the protein expression of DAPK1 and SENP1 in brain lysates from age-matched normal subjects (*n* = 4) and AD patients (*n* = 7) by immunoblotting analysis using indicated antibodies. A correlation between the expression levels of DAPK1 and SENP1 was established by linear regression analysis. The Pearson correlation coefficient *R*=-0.698. **(E-H)** Immunohistochemistry analysis of brain samples from age-matched normal subjects (*n* = 4) and AD patients (*n* = 7) showing the expression levels of DAPK1 and SENP1. Black square indicates an amplified field in each image. Scale bar = 100 μm and 25 μm, respectively. A correlation between the expression levels of DAPK1 and SENP1 was established by linear regression analysis. The Pearson correlation coefficient *R*=-0.7036. **(I)** SENP1 abundance in prefrontal cortex of normal control and AD patients from Bai et al., Neuron. 2020. **(J-L)** Brain lysates from age-matched normal subjects and AD patients were subjected to immunoprecipitation using HT7 antibody or anti-SUMO1 antibody, followed by immunoblotting analysis using anti-SUMO1 or anti-pT231-Tau antibodies, to determine levels of SUMOylated tau (K) or pT231-Tau (L), respectively. Input samples were analyzed using indicated antibodies. *n* = 3 samples/group. **(M-P)** Colocalization of SUMO1 with tau or pT231-Tau in the hippocampal tissues of age-matched normal subjects and AD patients using anti-SUMO1 and HT7 (M-N) or AT180 (O-P) antibodies, respectively. The Pearson’s correlation coefficient for SUMO1-positive and HT7 or AT180-positive signals for each cell was calculated to compare the colocalization ratios. 18–29 cells were randomly chosen from normal subjects (*n* = 4) and AD patients (*n* = 7), respectively. Representative images from triplicate repeats are shown. *p < 0.05, **p < 0.01, ***p < 0.001, ****p < 0.0001. Two-tailed unpaired *t*-test was used for analysis

To investigate the relationship between SENP1 and DAPK1 expression levels in human AD patients, we analyzed hippocampal samples from AD patients and age-matched controls. Immunoblotting analysis revealed elevated DAPK1 protein levels in AD brains compared with normal controls (Fig. 11A, B), which is consistent with our prior reports [35, 62], whereas SENP1 levels were reduced in brains of AD patients (Fig. 11A, C). There was an inverse correlation between DAPK1 and SENP1 levels, as indicated by Pearson correlation analysis (*R*= -0.698) (Fig. 11D). Moreover, the expression levels of DAPK1 and SENP1 in human brain samples were also confirmed using immunohistochemical analysis. The results revealed that AD brain patients had increased DAPK1 immunoreactivity and diminished SENP1 signals compared with control individuals (Fig. 11E-G). Similarly, Pearson correlation analysis confirmed a strong inverse correlation (*R*= -0.7036) between SENP1 and DAPK1 expression in human brains (Fig. 11H). These results align with a large-scale proteomic dataset, which reported reduced SENP1 levels in the cortex of AD patients compared with those of age-matched controls (Fig. 11I) [63]. To confirm the involvement of the DAPK1-SENP1-tau SUMOylation axis in regulating tau accumulation and phosphorylation in AD pathogenesis, we analyzed tau SUMOylation and phosphorylation in human brain samples. We observed that the SUMOylation of tau is significantly elevated in brain tissues from AD patients (Fig. 11J, K). Meanwhile, the upregulation of SUMOylation is also evident in pT231-Tau, according to the immunoprecipitation assay (Fig. 11J, L). These results coincide with dysregulated expression of DAPK1 and SENP1 in AD brains (Fig. 11A and J). Furthermore, co-immunostaining of SUMO1 with HT7 (total tau) or AT180 (pT231) antibodies revealed a robust colocalization of SUMO1 with tau species in the hippocampi of AD patients (Fig. 11M-P), which is in agreement with the immunoprecipitation data and supports the presence of aberrantly upregulated tau SUMOylation in AD. Overall, these data corroborate that DAPK1-induced alterations in SENP1 expression and tau SUMOylation are intimately associated with tau accumulation and hyperphosphorylation in the development of AD.

## Discussion

**Fig. 12:**
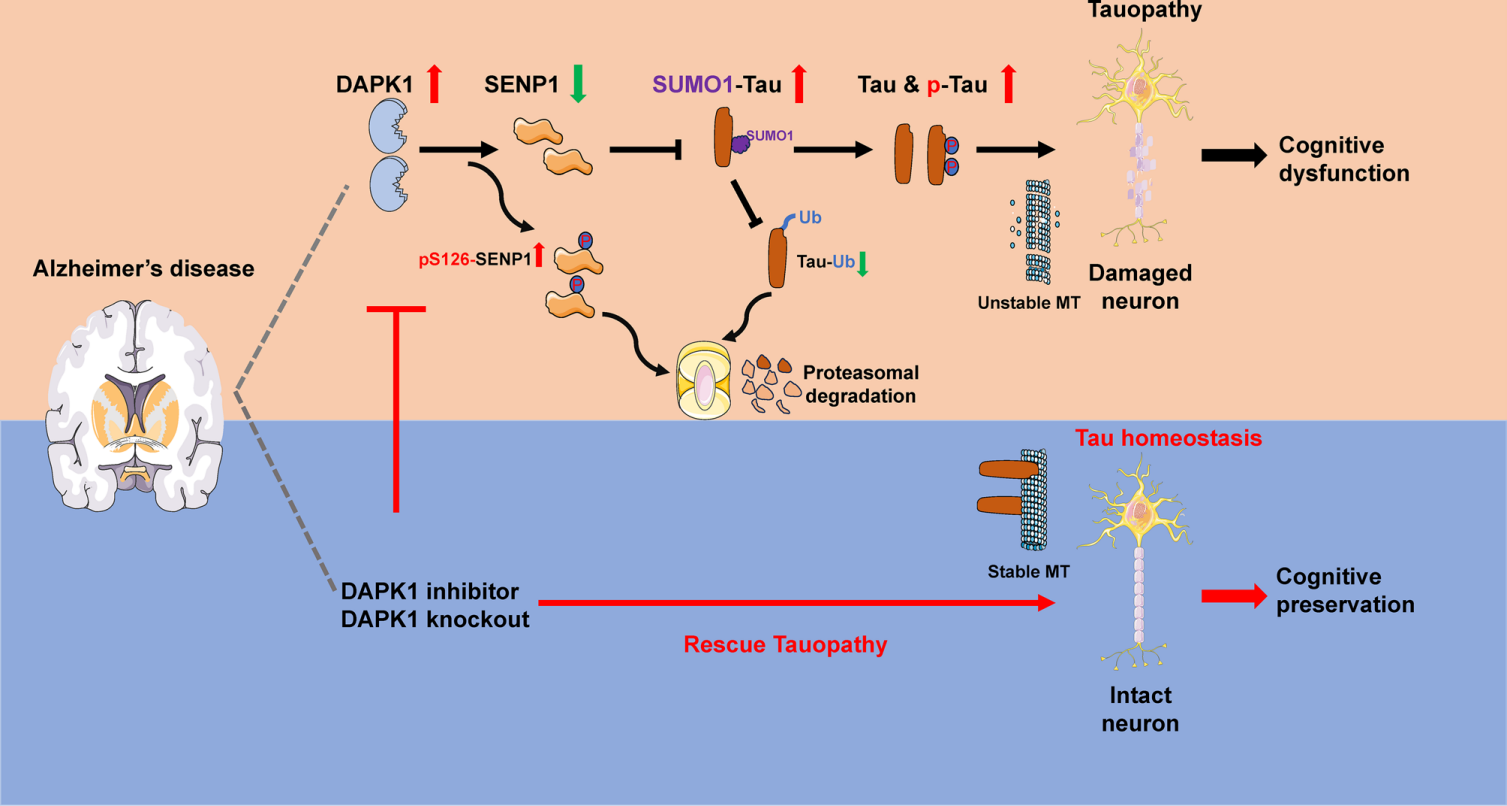
Schematic showing the role of DAPK1-SENP1-tau SUMOylation axis in the development of tauopathy and cognitive dysfunction in AD. DAPK1 phosphorylates SENP1 at Ser126, leading to the proteasome-dependent degradation of SENP1 in neurons. SENP1 deficiency upregulates tau SUMOylation, resulting in an imbalance in tau ubiquitination, which ultimately contributes to increased tau phosphorylation and accumulation. Pharmacological inhibition or genetic ablation of DAPK1 normalizes SENP1 expression and tau SUMOylation, thus restoring neuronal tau proteostasis and protecting neuronal functions

The accumulation of aberrantly modified tau species is tightly associated with cognitive deterioration during AD progression. Although cumulative evidence indicates that DAPK1 may play a critical role in tau pathology and AD, the underlying mechanisms remain incompletely understood. In the present study, DAPK1 was shown to be a critical regulator of tau homeostasis in vitro and in vivo by regulating SENP1 phosphorylation and degradation, thereby influencing tau SUMOylation and phosphorylation in neurons. DAPK1 ablation or inhibition alleviates synaptic damage and gliosis, and improves cognitive function by attenuating tau SUMOylation and accumulation (Fig. 12).

We systematically analyzed the interaction between DAPK1 and SENP1, and identified the binding domains responsible for the interaction (Figs. 1 and S1). DAPK1 affects the level of SENP1 in a kinase activity-dependent manner, and its binding to SENP1 is independent of the catalytic activity. However, the binding of DAPK1 to SENP1 alone cannot regulate SENP1 expression, suggesting that other regulatory mechanisms, including PTMs, may be needed. Notably, two potential DAPK1 phosphorylation motifs in SENP1 were identified by mass spectrometric analysis. In in vitro experiments, we demonstrated that DAPK1 may target only Ser126 of SENP1 to regulate its function (Figs. 2 and 3). The current knowledge regarding the regulation of SENP1 activity and function remains limited. Previous studies have shown that SENP1 is regulated through PTMs, including phosphorylation. For example, T-cell receptor-induced phosphorylation of SENP1 at Tyr270 has been reported to enhance SENP1 isopeptidase activity [40]. We showed that DAPK1 does not affect the transcription of SENP1 but influences the ubiquitination-dependent degradation of SENP1 by phosphorylating the Ser126 residue (Figs. 2 and 3). Deletion of the 848–1288 aa domain of DAPK1 and the SENP1 S126A mutant effectively abolish the regulation of SENP1 level by DAPK1. In human AD samples and hTau mouse models, the expression and enzymatic activity of DAPK1 were found to be upregulated, respectively, resulting in a reduction in SENP1 protein levels in the hippocampus (Figs. 11 and S9). DAPK1 inhibition or depletion successfully restored SENP1 expression in neurons (Figs. 5 and 8), further supporting an upstream role for DAPK1-mediated phosphorylation in modulating SENP1 levels. However, the exact mechanisms underlying S126 phosphorylation-induced SENP1 degradation need further characterization. The S126 phosphorylation likely activates the ubiquitination-dependent degradation cascade of SENP1, thus facilitating its proteasomal degradation in neurons.

SENP1 modulates the PTM landscape of target proteins, thereby controlling downstream protein function and/or stability. Specifically, SENP1 functions as a SUMO-specific protease that reverses SUMO conjugation, thus altering protein activity, subcellular localization, and degradation kinetics [64]. SUMOylation plays an essential role in regulating fundamental processes critical to neuronal homeostasis. It has been reported that tau and amyloid precursor protein, two key proteins involved in AD pathogenesis, undergo SUMOylation both in vitro and in vivo [23, 65]. However, the upstream mechanisms responsible for dysregulated tau SUMOylation remain unclear. Our study indicates that DAPK1-induced SENP1 downregulation is intimately involved in tau SUMOylation. Tau is a direct substrate of SENP1, as revealed by the colocalization of two proteins and the direct deSUMOylation of tau catalyzed by SENP1 but not the inactive form (Figs. 4 and S7). Tau is SUMOylated at Lys340 mainly by SUMO1. A previous study showed that the protein inhibitor of activated STAT family acts as SUMO E3 ligases to facilitate tau SUMOylation, and that SENP family serves as deSUMOylation enzymes to reduce tau SUMOylation [50]. Our findings align with previous studies and demonstrate that SENP1 reduces the level of SUMO1-conjugated tau. SENP1-mediated tau deSUMOylation is an essential process that mediates the effect of DAPK1 on tau PTMs as SENP1 KO abolished DAPK1 overexpression-induced accumulation of SUMOylated tau.

SUMOylation is important for the phosphorylation and turnover of tau in neurons. However, the downstream effect of tau SUMOylation may be determined by specific SUMO ligases. Zhang et al. reported that tripartite motif 11 conjugates SUMO2/3 to tau, which subsequently enhances neuronal tau degradation [2]. However, SUMO1 conjugation suppresses tau ubiquitination and degradation, leading to the accumulation of hyperphosphorylated tau in neurons [7]. In our study, we showed that SUMO1 modification enhances tau phosphorylation at multiple AD-related sites, a process inhibited by SENP1 overexpression and DAPK1 inhibition (Figs. 4, 5 and 8). These findings implicate that SENP1 may have a neuroprotective role in the brain. Previous studies have shown that the transcriptional suppression of SENP1 reduces the expression of glutamate receptor 1, a key component for learning and memory [66]. Notably, SENP1 exerts neuroprotective effects against ischemia-reperfusion injury by reducing SUMO1 conjugation and attenuating apoptosis [28]. The decline in cognitive function caused by intermittent hypoxia (IH) is linked to neuroinflammatory processes involving microglia. SENP1-mediated deSUMOylation of nuclear factor κB essential modulator, β-catenin, peroxisome proliferator-activated receptor γ, and the target of Myb1 in microglia antagonizes the inflammatory response and cognitive impairment induced by IH [29, 30, 67, 68]. Apart from directly contributing to tau deSUMOylation, SENP1 downregulation may also indirectly participate in tau pathology through regulating the SUMOylation of SET and p53, after which protein phosphatase 2 A is inhibited and tau phosphorylation is elevated [69, 70]. Although the upregulation of neuronal SENP1 expression might protect against tau-related pathologies, a recent study reported that β-amyloid treatment suppresses glycolysis in astrocytes by increasing SENP1 transcription, and that SENP1 knockdown confers neuroprotection in APP/PS1 mouse model [71]. Combined, we propose that the function of SENP1 in the brain may also depend on the cell type, and that strategies targeting SENP1 for neurological diseases should be carefully evaluated to minimize potential side effects.

Hippocampal DAPK1 protein levels are elevated in hTau transgenic mouse model compared with WT mice [18], whereas in our hTau virus injection model, no increase in DAPK1 expression was observed. Notably, DAPK1 enzymatic activity was increased after hTau overexpression in hippocampus (Fig. S9). The molecular mechanisms underlying DAPK1 activation by hTau overexpression remains elusive. It is possible that hippocampal hTau accumulation triggers cellular calcium dyshomeostasis, thereby leading to the hyperactivation of DAPK1 in neurons [72, 73]. Previous studies have revealed that DAPK1 regulates tau phosphorylation and stability by different pathways. For example, DAPK1 interacts with Pin1 and inhibits its activity by directly phosphorylating the Ser71 residue of Pin1, thus blocking the *cis*-to-*trans* isomerization of the phospho-Thr231-Pro motif in tau protein and ultimately resulting in the accumulation of *cis* pT231-Tau [14, 19]. DAPK1 has also been reported to directly interact with and phosphorylates tau at the Ser262 residue, thereby exacerbating neuronal tau pathology [49]. In addition, Wu et al. demonstrated that DAPK1 can bind to and activate MARK2, thus leading to tau phosphorylation at Ser262 and the subsequent microtubule disruption [22]. In this study, we identified another pathway in which DAPK1 enhances tau SUMOylation by phosphorylating SENP1 at Ser126 (Fig. 12). These findings establish SENP1 as a direct link between DAPK1 activity and the regulation of tau SUMOylation. Upregulation of Tau SUMOylation has been noted in brains of AD and progressive supranuclear palsy patients, and is tightly connected with tau hyperphosphorylation and intraneuronal accumulation [74]. Consistently, we found that colocalization of SUMO1 and tau species was also elevated in the hippocampus of AD patients (Fig. 11). Therefore, dysregulated tau SUMOylation can drive the development of aberrant tau modification and impaired proteostasis in neurological diseases. Our research suggests that DAPK1-induced SENP1 phosphorylation causes the degradation of SENP1, leading to the accumulation of SUMOylated tau and abnormal tau phosphorylation in AD (Fig. 12). This mechanism provides a plausible explanation for the observed increase in tau SUMOylation in AD brains and its contribution to cognitive dysfunction.

The overexpression of human 2N4R tau in hippocampal CA3 region leads to the accumulation of tau species as well as tau hyperphosphorylation, further resulting in synaptic degeneration and gliosis in the hippocampus. All these pathological changes contribute to cognitive dysfunction and memory deficits in behavioral tests. DAPK1 ablation or inhibition mitigates tau pathology and ameliorates synaptic dysfunction and gliosis in the mouse models (Figs. 6 and 9). While these results align with previous studies linking DAPK1 to tau, our work elucidates SENP1 regulation as the mechanistic basis for the effect of DAPK1 on tau pathology. Our data demonstrate that genetic ablation or pharmacological inhibition of DAPK1 alleviates the accumulation of SUMOylated tau, consequently resolving tau hyperphosphorylation in neurons (Figs. 5 and 8). The normalization of tau SUMOylation by DAPK1 ablation or inhibition improves the cognitive performance and ameliorates emotional abnormalities in hTau mouse model as measured by different behavioral tests (Figs. 7 and 10). Given that DAPK1 is a potential risk gene for late-onset AD, and its expression is elevated in the brains of AD patients, our data from human AD brain samples reinforce the clinical importance of DAPK1-SENP1-mediated tau SUMOylation in the pathogenesis of AD-related tau pathology. However, the human sample data on DAPK1 and tau SUMOylation is correlative, and a definitive validation of the DAPK1/SENP1/tau-SUMOylation axis in the development of tauopathy and other AD pathologies would require the use of human AD patient-derived induced pluripotent stem cells for disease modeling.

Our study has several limitations. First, the lack of specific antibody for SENP1 phosphorylation at Ser126 prevented us from determining SENP1 phosphorylation levels in brains of human AD patients and tauopathy mouse models. Second, the overexpression of human 2N4R tau in hippocampal CA3 region recapitulates the early stage pathological features of tauopathy including synaptic dysfunction and neuroinflammation, while it does not develop typical tau pathologies such as neurofibrillary tangles, and the pathological changes are mostly confined to virus-infused CA3 area. It is worthwhile to validate the pathological relevance of DAPK1/SENP1 axis using transgenic mouse models of tauopathy in the future. Since both DAPK1 and SENP1 are ubiquitously expressed in multiple brain regions, whether they are also involved in regulating tau SUMOylation in other brain regions remains further characterization using appropriate models. Besides, the potential involvement of other SENP members such as SENP2 or SENP-like proteases in DAPK1-induced dysregulation of tau SUMOylation warrants further investigation. Finally, since DAPK1 plays a crucial role in non-neuronal cells, targeted delivery of specific DAPK1 inhibitors is essential for the intervention of AD.

## Conclusion

DAPK1 interacts with SENP1 and directly phosphorylates its Ser126 residue, thereby promoting SENP1 degradation via the ubiquitin-proteasome pathway. This degradation cascade elevates SUMOylated tau levels, subsequently exacerbating tau hyperphosphorylation and aggregation. Critically, either genetic ablation of DAPK1 or pharmacological inhibition of its kinase activity significantly attenuates cognitive impairment and tau-associated neuropathologies. These results demonstrate the multifaceted role of DAPK1 and the essential contribution of DAPK1-induced SENP1 dysregulation in regulating neuronal tau pathology, and provide potential candidate targets for the development of treatment strategies for AD.

## Supplementary Information

Below is the link to the electronic supplementary material.

Supplementary Material 1: Full and uncropped western blot images.

Supplementary Material 2: Supplementary table S1: Information of antibodies used in the present study. Supplementary table S2: Sequence information of qRT-PCR. Supplementary table S3: Information for human brain samples used in immunoblotting analysis. Supplementary table S4: Information for human brain samples used in immunohistochemistry and immunofluorescence imaging analyses. Figure S1: DAPK1 interacts with SENP1 in vitro and in vivo. Figure S2: DAPK1 does not influence the transcription of SENP1. Figure S3: The 848-1288 aa domain of DAPK1 does not affect the degradation of SENP1. Figure S4: Information of identified phosphorylation sites of SENP1 by DAPK1 in the mass spectrometry. Figure S5: DAPK1 phosphorylates SENP1 at Ser126 in vitro. Figure S6: SENP1 is increased in hippocampus and cortex of DAPK1-KO mice. Figure S7: SENP1 deSUMOylates tau and affects its phosphorylation and ubiquitination. Figure S8: Construction and validation of SENP1 knockout cell line. Figure S9: Establishment of the mouse model for tauopathy and the characterization of SENP1 expression in different cell types in the brain. Figure S10: Characterization of body weight change and DAPK1 activity in mice treated with or without the DAPK1 inhibitor C6.

## Data Availability

The data supporting the conclusions of this study are presented in the main text and the Supplementary Materials. Additional data and materials can be obtained from the corresponding authors upon request.

## Abbreviations

AD: Alzheimer’s disease
ANOVA: Analysis of variance
CHX: Cycloheximide
COR: C-terminal of ROC domain
DAPK1: Death-associated protein kinase 1
EPM: Elevated plus maze
GFAP: Glial fibrillary acidic protein
GFP: Green fluorescent protein
GST: Glutathione S‑transferase
IBA1: Ionized calcium-binding adaptor molecule 1
IF: Immunofluorescence assays
IHC: Immunohistochemistry assays
IH: Intermittent hypoxia
KO: Knockout
MAP2: Microtubule-associated protein 2
MWM: Morris water maze
MARK2: Microtubule-affinity regulating kinase 2
MLC: Myosin light chain
NFTs: Neurofibrillary tangles
NOR: Novel object recognition
NA: Novel arm
OFT: Open field test
PSD95: Postsynaptic density protein-95
PTMs: Posttranslational modifications
rAAV: recombinant adeno-associated virus
SENP1: Sentrin-specific protease 1
SUMO: Small ubiquitin-like modifier
shRNA: short hairpin RNA
WT: Wild-type
