# Supplementary material for: Death-associated protein kinase 1-dependent SENP1 degradation increases tau SUMOylation and leads to cognitive dysfunction in a mouse model for tauopathy
Source: Mol Neurodegener. 2025 Nov 21;20:121. doi: 10.1186/s13024-025-00911-3 (PMC12639696; doi:10.1186/s13024-025-00911-3)
Supplement: Supplementary file 1 — Supplementary Material 1: Full and uncropped western blot images. [file 13024_2025_911_MOESM1_ESM.pdf]

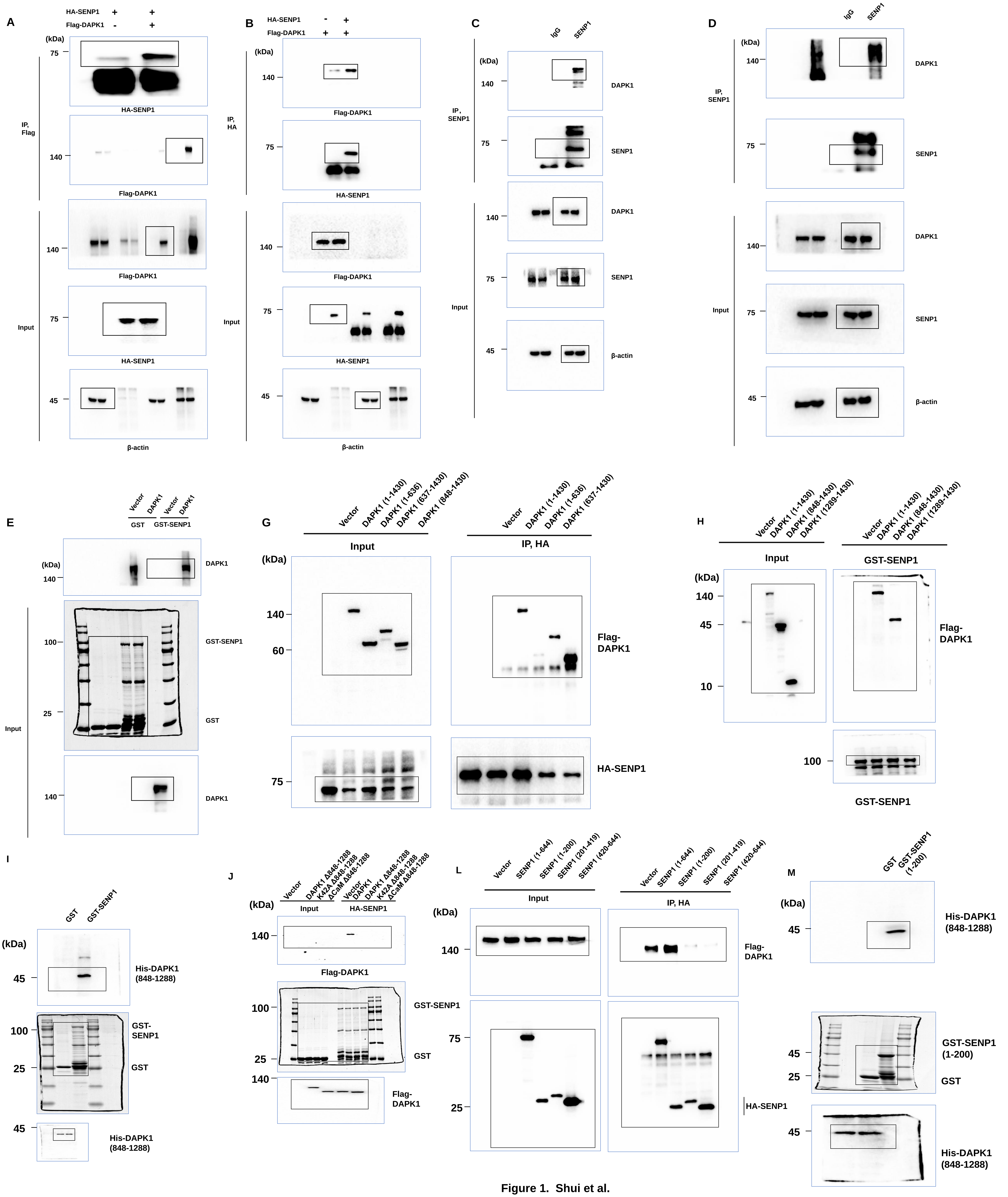

Figure 1. Shui et al.

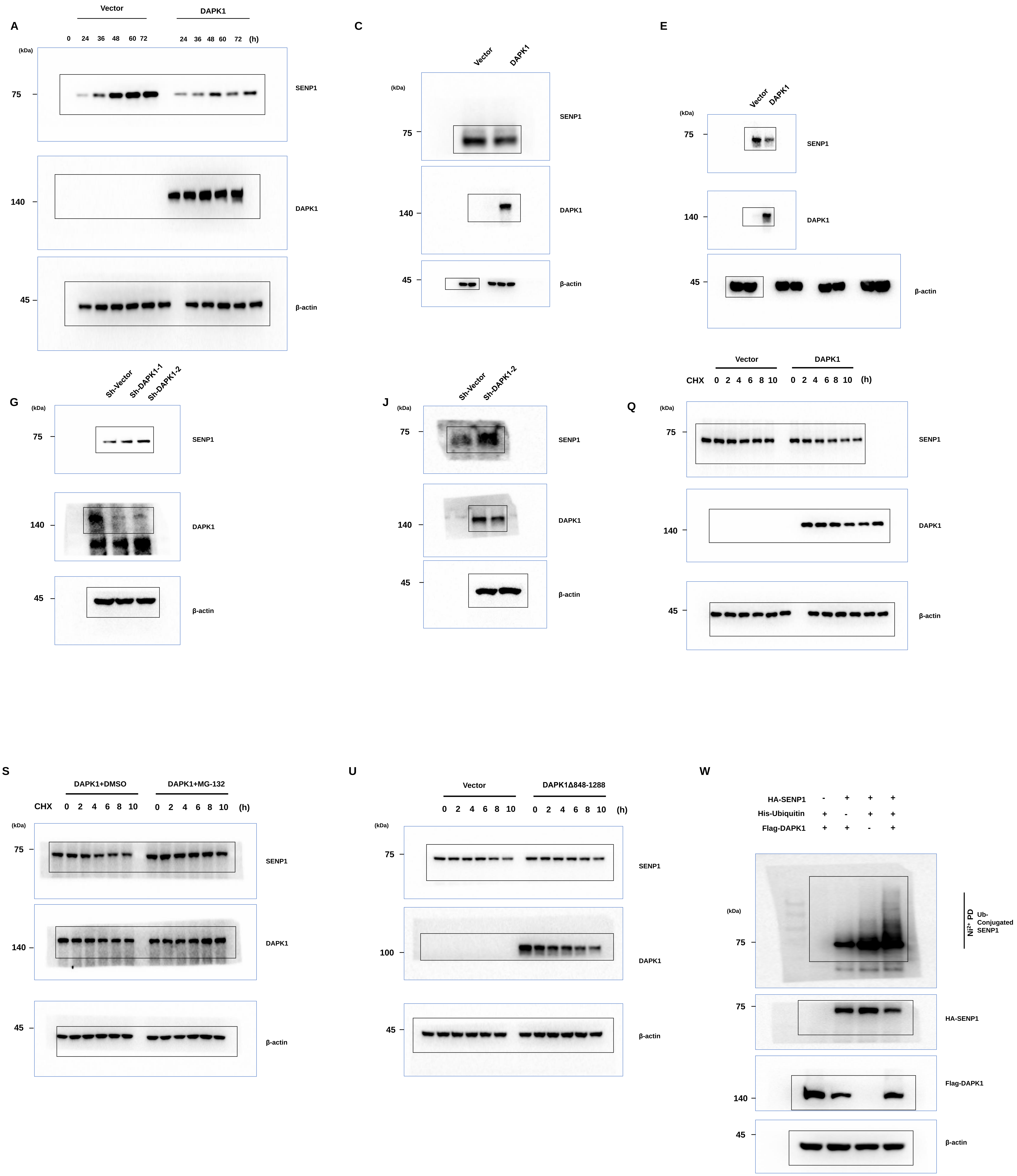

Figure 2. Shui et al.

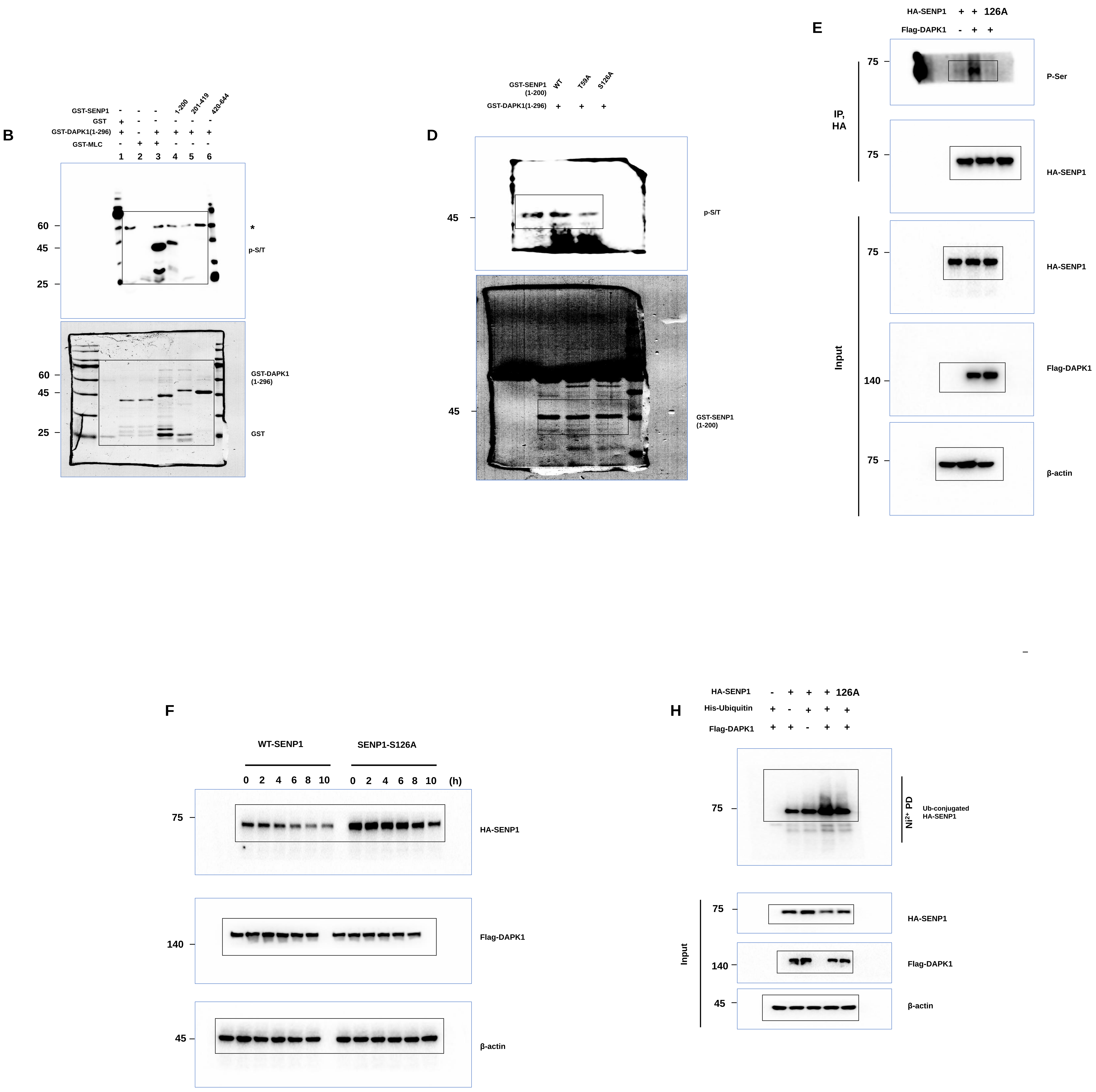

Figure 3. Shui et al.

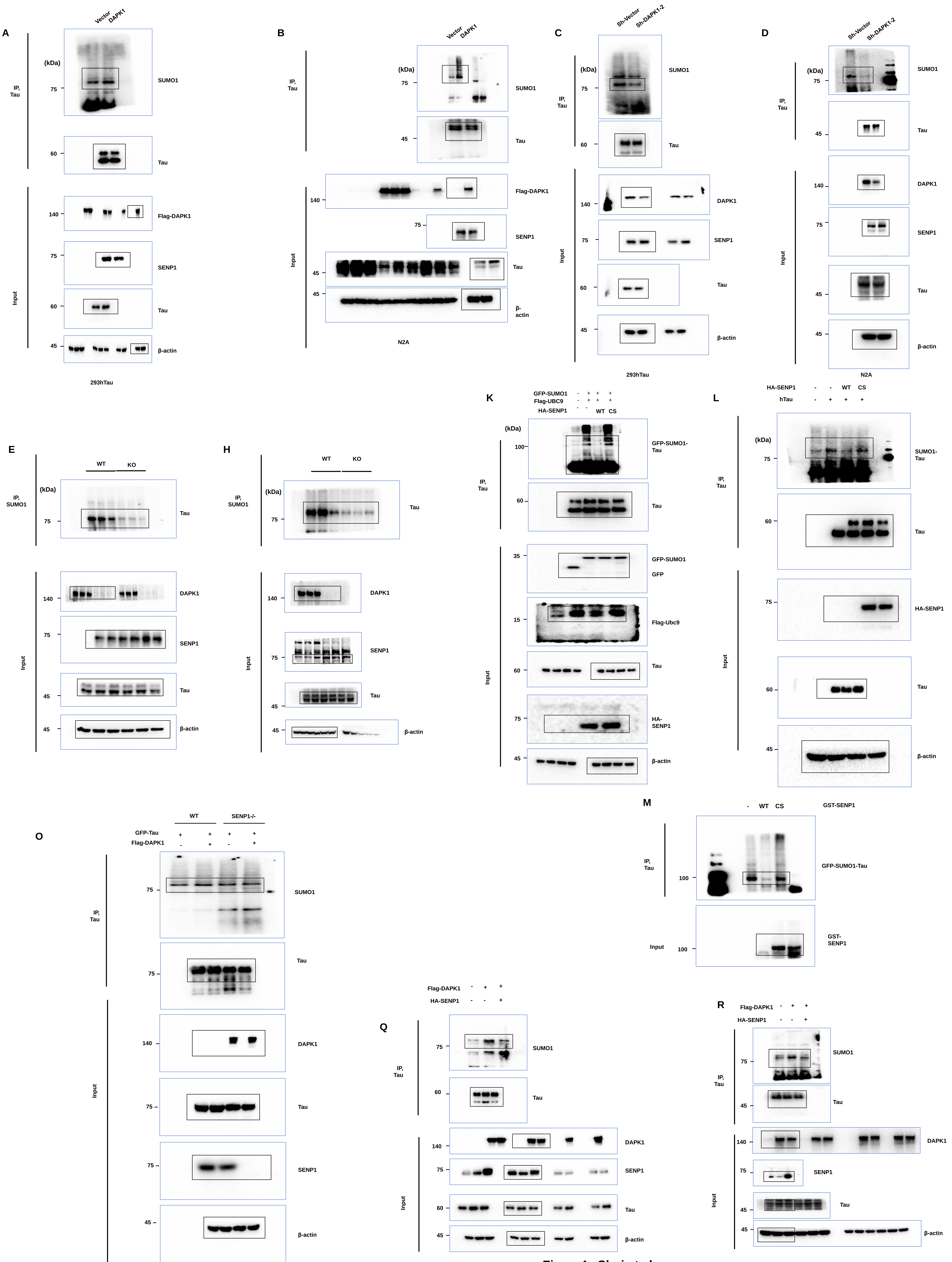

Figure4. Shui et al.

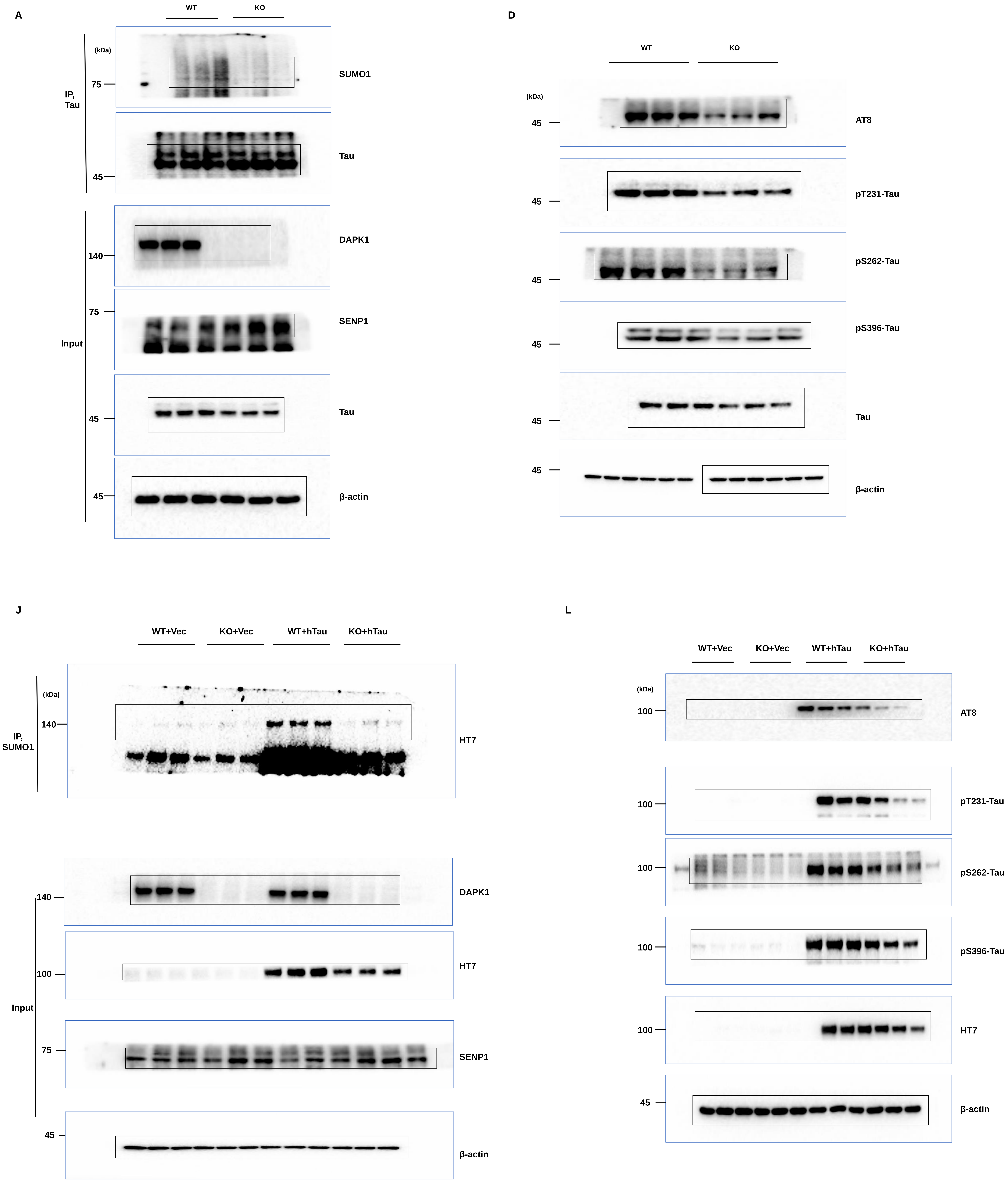

Figure 5 Shui et al.

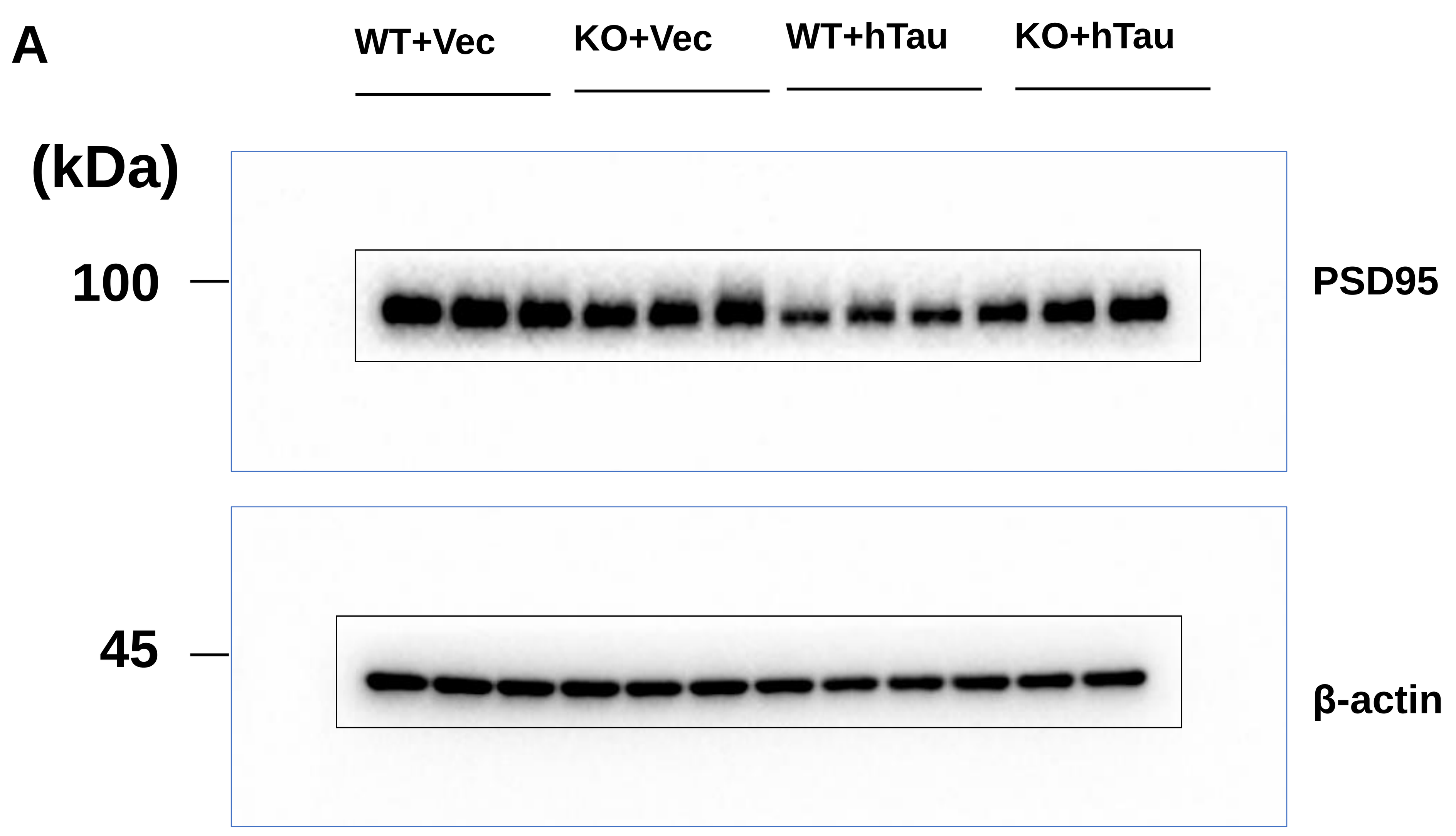

Figure 6. Shui et al.

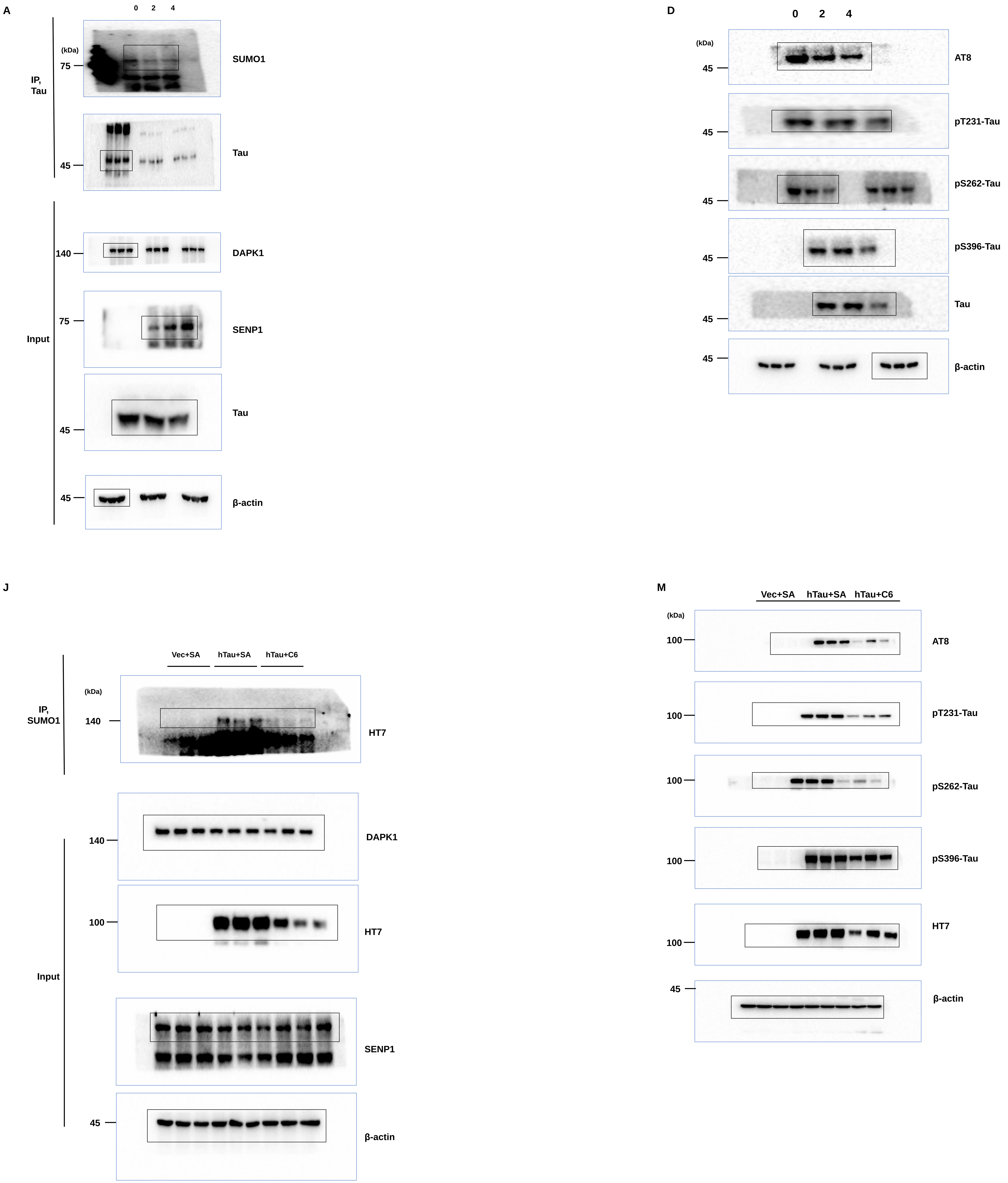

Figure 8. Shui et al.

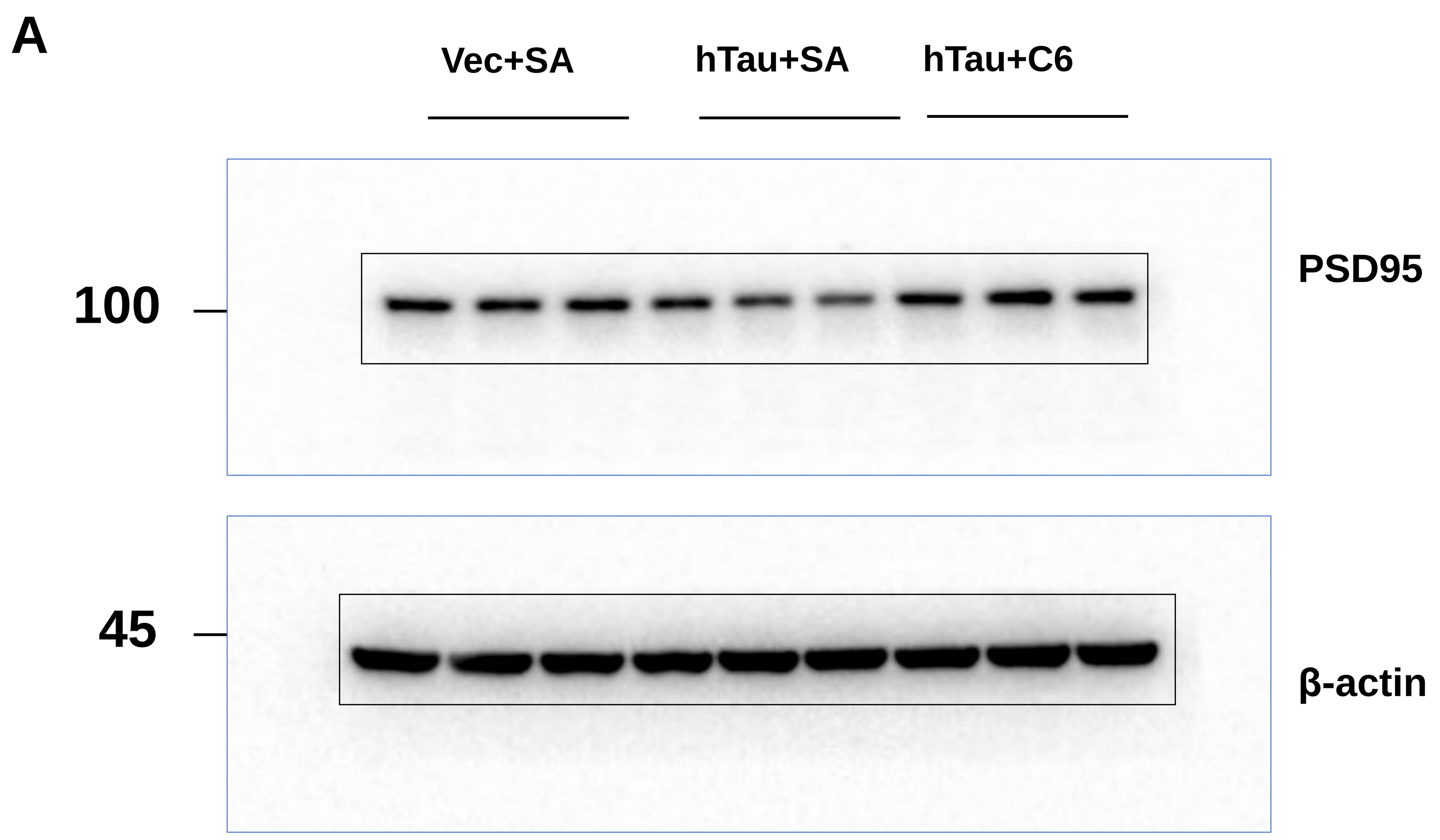

Figure 9 Shui et al.

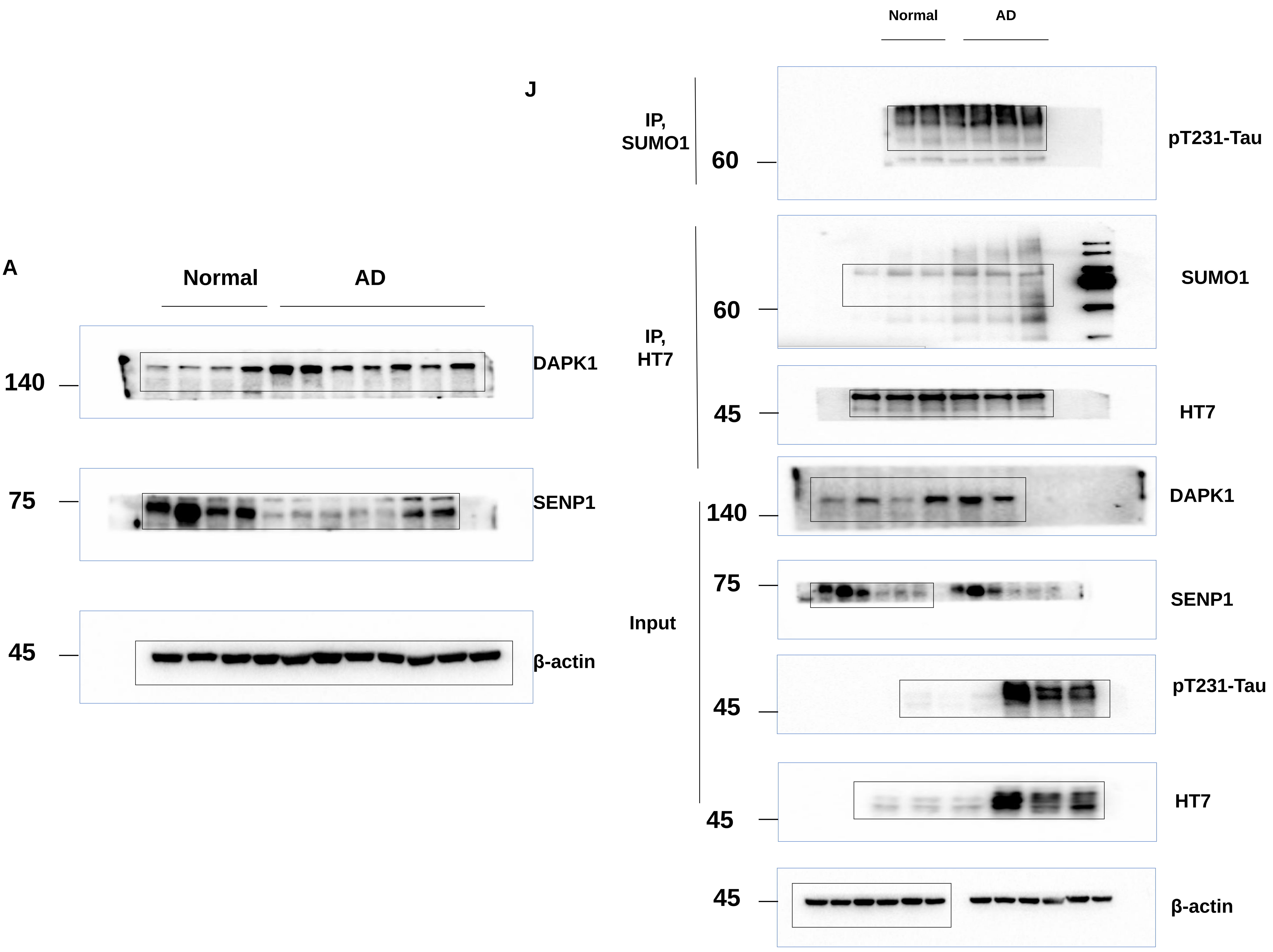

Figure 11 Shui et al.

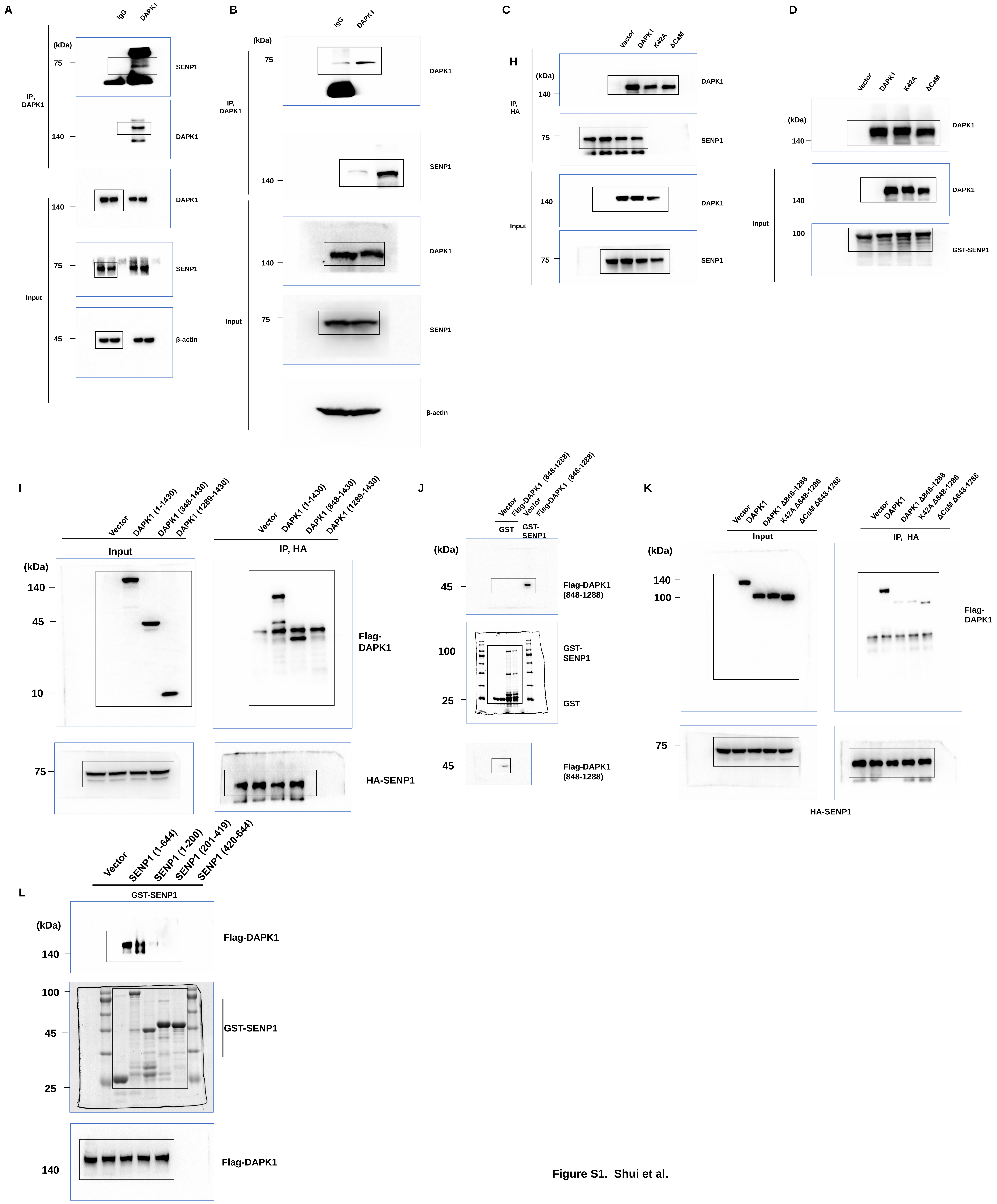

Figure S1. Shui et al.

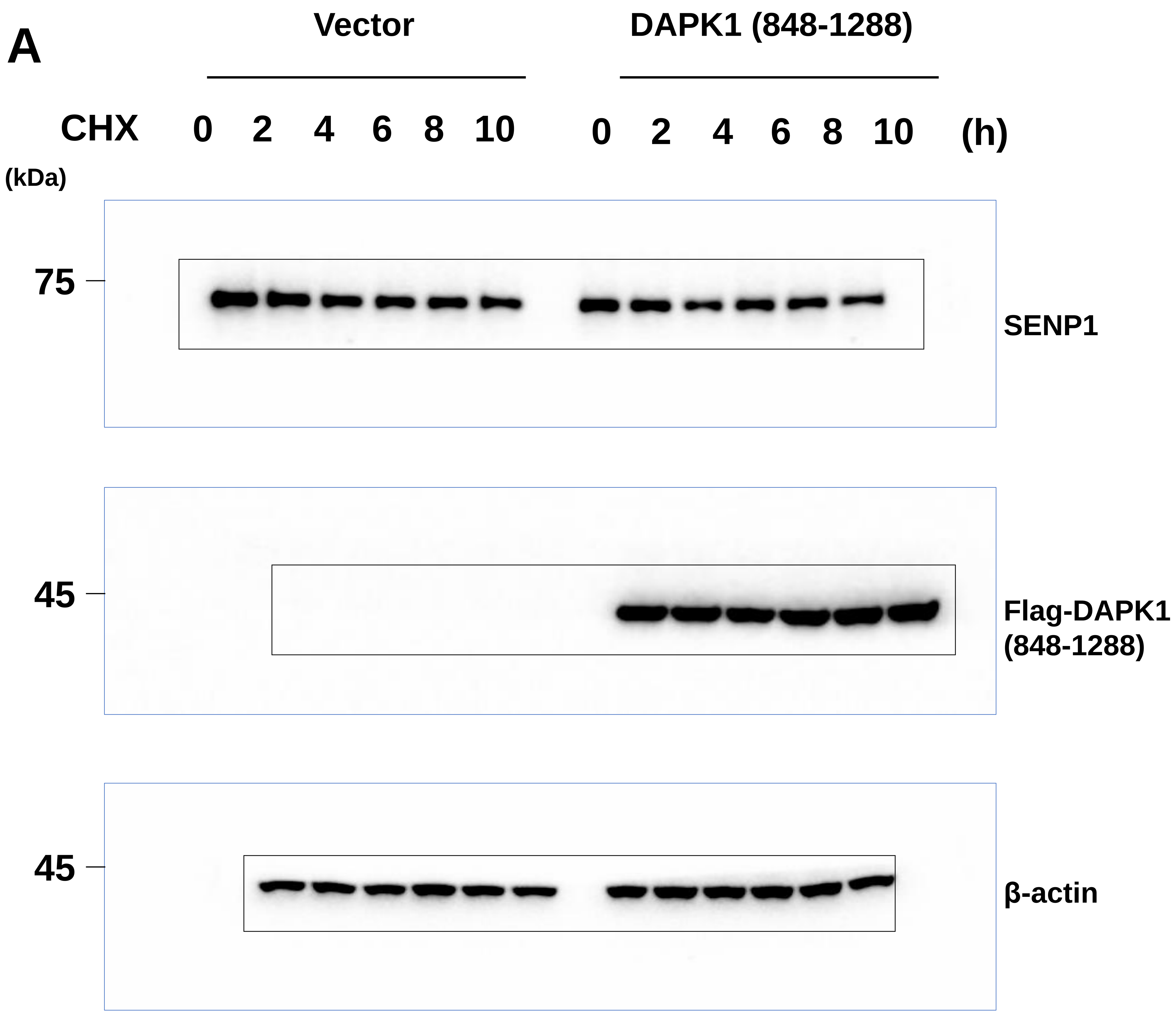

Figure S3 Shui et al.

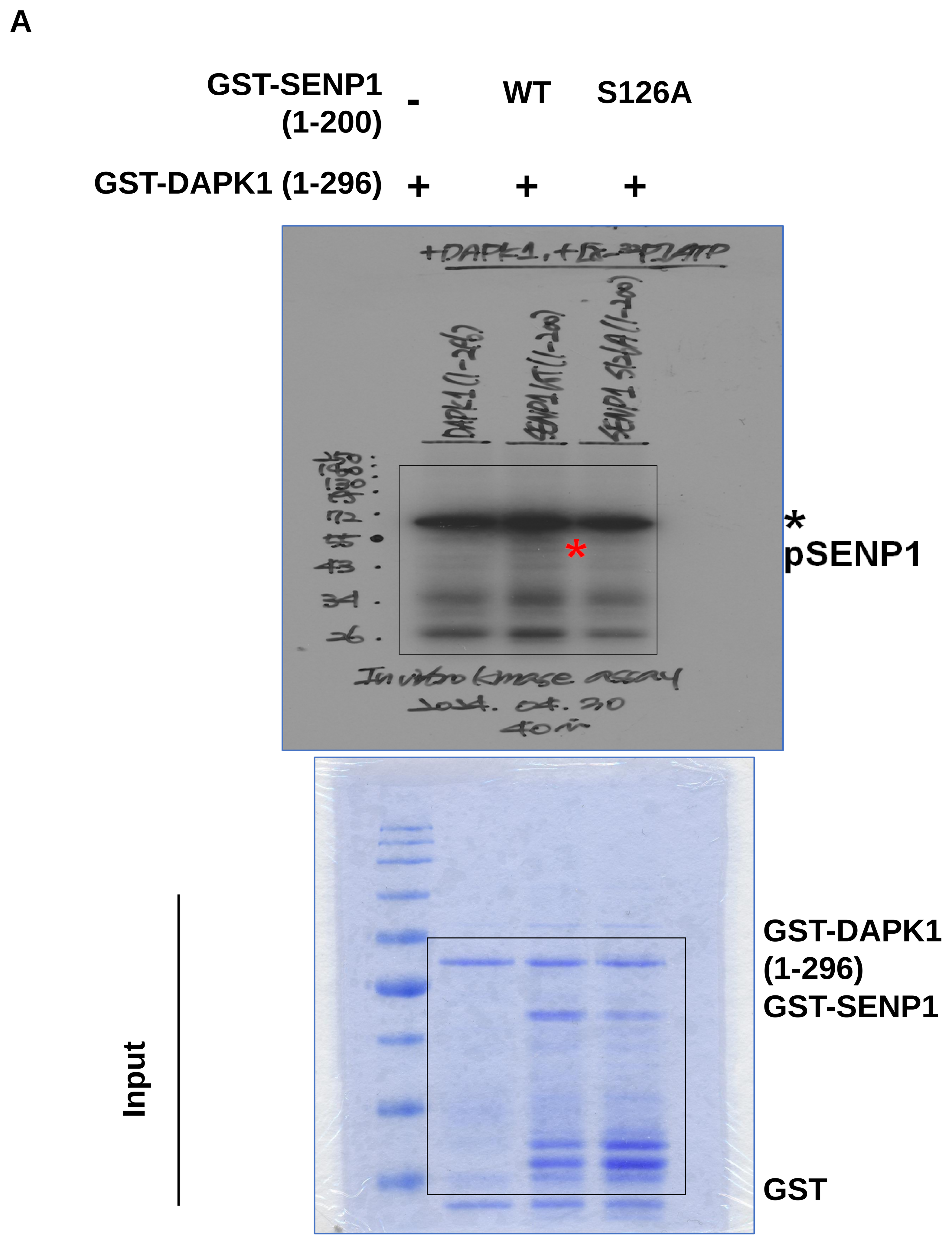

Figure S5 Shui et al.

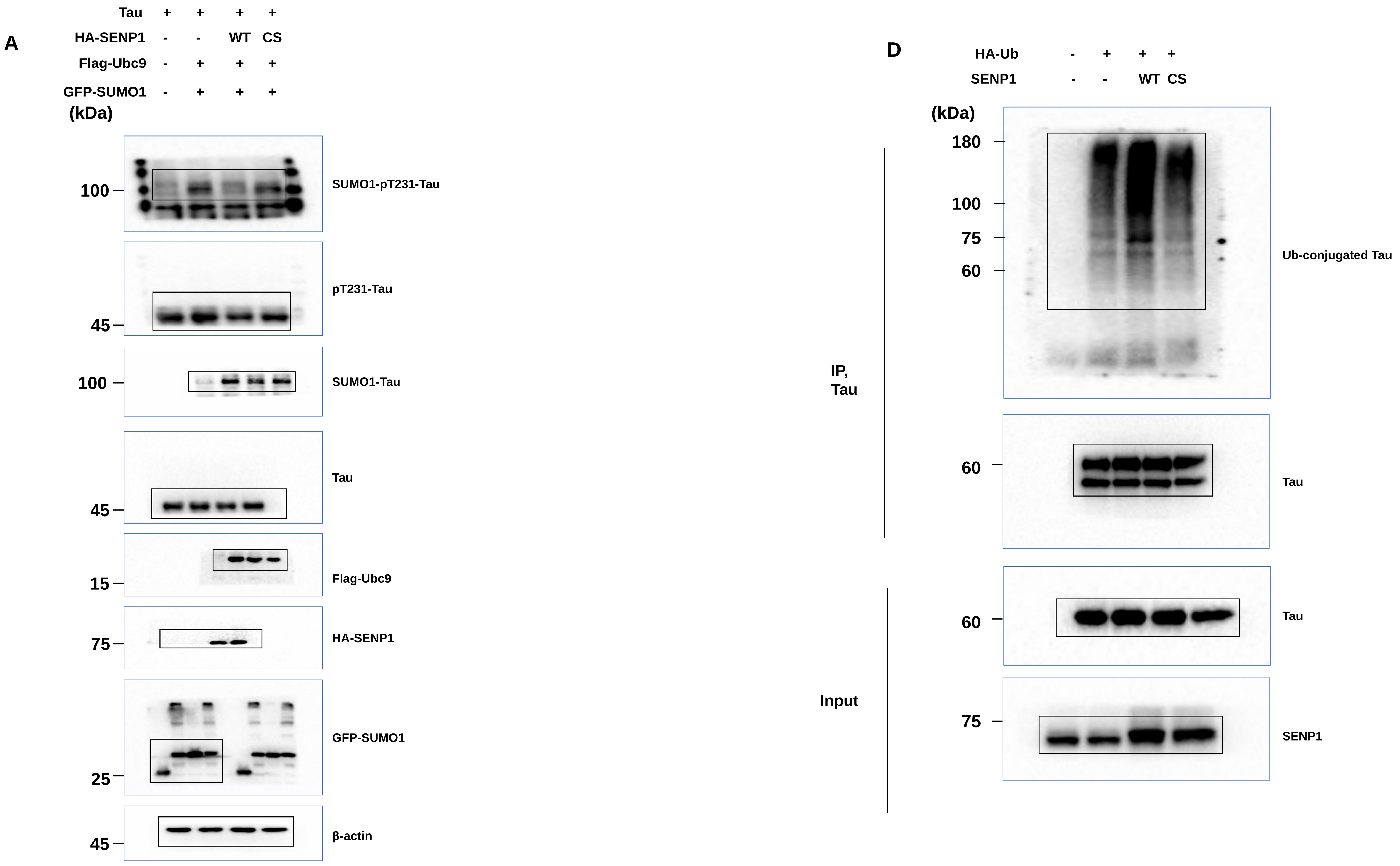

Figure S7 Shui et al.

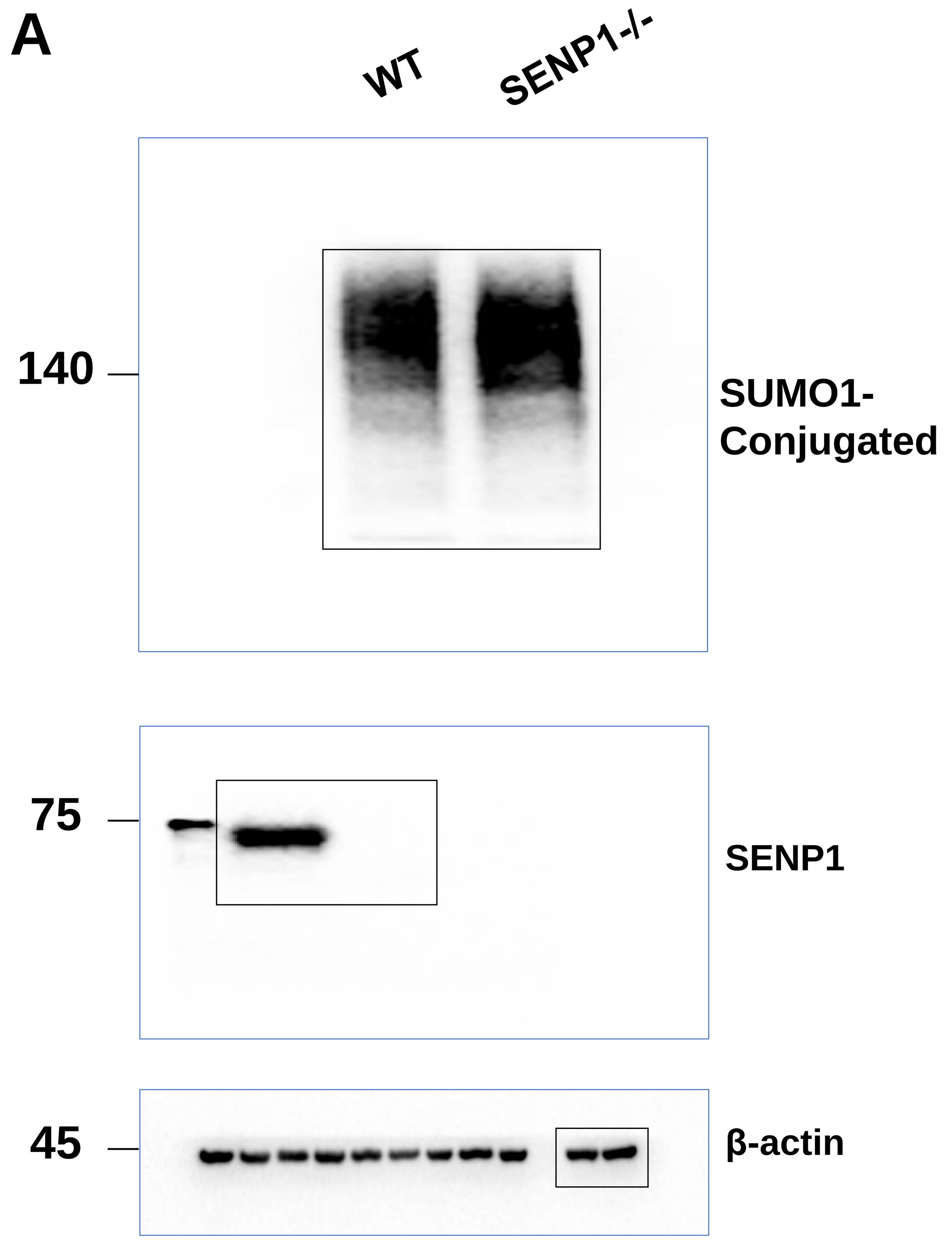

Figure S8 Shui et al.

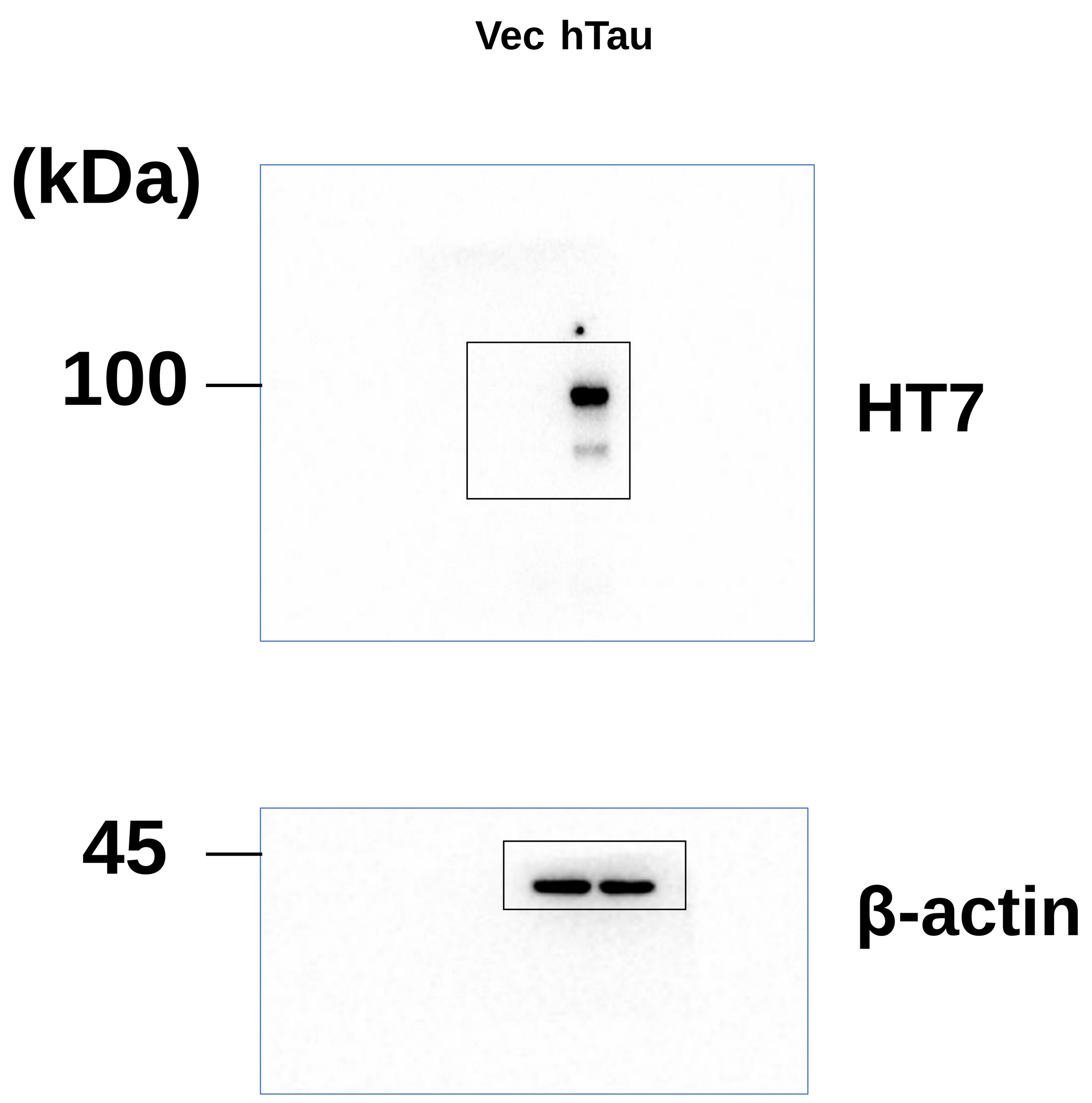

Figure S9 Shui et al.
