# Supplementary material for: Death-associated protein kinase 1-dependent SENP1 degradation increases tau SUMOylation and leads to cognitive dysfunction in a mouse model for tauopathy
Source: Mol Neurodegener. 2025 Nov 21;20:121. doi: 10.1186/s13024-025-00911-3 (PMC12639696; doi:10.1186/s13024-025-00911-3)
Supplement: Supplementary file 2 — Supplementary Material 2: Supplementary table S1: Information of antibodies used in the present study. Supplementary table S2: Sequence information of qRT-PCR. Supplementary table S3: Information for human brain samples used in immunoblotting analysis. Supplementary table S4: Information for human brain samples used in immunohistochemistry and immunofluorescence imaging analyses. Figure S1: DAPK1 interacts with SENP1 in vitro and in vivo. Figure S2: DAPK1 does not influence the transcription of SENP1. Figure S3: The 848-1288 aa domain of DAPK1 does not affect the degradation of SENP1. Figure S4: Information of identified phosphorylation sites of SENP1 by DAPK1 in the mass spectrometry. Figure S5: DAPK1 phosphorylates SENP1 at Ser126 in vitro. Figure S6: SENP1 is increased in hippocampus and cortex of DAPK1-KO mice. Figure S7: SENP1 deSUMOylates tau and affects its phosphorylation and ubiquitination. Figure S8: Construction and validation of SENP1 knockout cell line. Figure S9: Establishment of the mouse model for tauopathy and the characterization of SENP1 expression in different cell types in the brain. Figure S10: Characterization of body weight change and DAPK1 activity in mice treated with or without the DAPK1 inhibitor C6. [file 13024_2025_911_MOESM2_ESM.pdf]

**Supplementary information**

# Death-associated protein kinase 1-dependent SENP1 degradation increases tau SUMOylation and leads to cognitive dysfunction in a mouse model for tauopathy

Xindong Shui[1], Xiaoqing Zheng[1], Jinfeng Wu[2], Mi Zhang[1], Gamin Kim[3], Renxuan Chen[1], Lianlian Peng[1], Zonghai Wang[1], Yameng Zheng[4], Ling Zhang[1], Ruomeng Li[1], Long Wang[1], Ying Zhou[1], Jungho Kim[3], Dongmei Chen[1, *], Tao Zhang[1, **], Tae Ho Lee[1, ***]

[1]Fujian Key Laboratory of Cognitive Function and Diseases
Institute of Basic Medicine
School of Basic Medical Sciences
Fujian Medical University, Fuzhou, Fujian, China
[2]Key Laboratory of Gastrointestinal Cancer (Ministry of Education)
School of Basic Medical Sciences
Fujian Medical University, Fuzhou, Fujian, China
[3]Laboratory of Molecular and Cellular Biology
Department of Life Science
Sogang University, Seoul, Korea
[4]Fujian Key Laboratory of Molecular Neurology
Institute of Neuroscience
Fujian Medical University, Fuzhou, Fujian, China

**Running title:** DAPK1 promotes tau SUMOylation and tau pathology by inhibiting SENP1

*Corresponding author: Dongmei Chen, Fujian Medical University, 1 Xuefu North Road, Fuzhou, Fujian 350122, China; E-mail: dmchen88@fjmu.edu.cn; Tel.: +86-591-2286-2498; Fax: +86-591-2286-2320.

**Corresponding author: Tao Zhang, Fujian Medical University, 1 Xuefu North Road, Fuzhou, Fujian 350122, China; E-mail: taozh@fjmu.edu.cn; Tel.: +86-591-2286-2498; Fax: +86-591-2286-2320.

***Corresponding author: Tae Ho Lee, Fujian Medical University, 1 Xuefu North Road, Fuzhou, Fujian 350122, China; E-mail: tlee0813@fjmu.edu.cn; Tel.: +86-591-2286-2498; Fax: +86-591-2286-2320.

**Table S1. Information of antibodies used in the present study.**

| Antibody | Dilution | Source | Identifier |
|---|---|---|---|
| Rabbit anti-pT231-Tau | 1:7000 (IB)<br>1:1000 (IF) | Abcam | ab151559 |
| Rabbit anti-pS262-Tau | 1:2000 | Invitrogen | 44750G |
| Rabbit anti-pS396-Tau | 1:5000 | Abcam | ab109390 |
| Rabbit anti-AT8 | 1:2000 | Invitrogen | MN1020 |
| Mouse anti-Tau (HT7) | 1:10000 (IB)<br>1:500 (IF) | Invitrogen | MN1000 |
| Mouse anti-Tau5 | 1:5000 (IB) | Invitrogen | AHB0042 |
| Mouse anti-DAPK1 | 1:2000 (IB) | Sigma | D2178 |
| Rabbit anti-DAPK1 | 1:50 (IHC) | Sigma | SAB4500620 |
| Mouse anti-β-actin | 1:50000 (IB) | Sigma | A5441 |
| Rabbit anti-PSD95 | 1:1000 (IB) | Abcam | ab18258 |
| Rabbit anti-MAP2 | 1:50 (IF) | Cell Signaling Technology | 4542S |
| Mouse anti-NeuN | 1:400 (IF) | Millipre | MAB377 |
| Mouse anti-GFAP | 1:500 (IF) | Santa cruz | sc-33673 |
| Mouse anti-IBA1 | 1:1000 (IF) | Abcam | ab283319 |
| Rabbit anti-GFAP | 1:500 (IF) | Abcam | ab7260 |
| Rabbit anti-IBA1 | 1:500 (IF) | Abcam | ab178847 |
| Rabbit anti-SUMO1 | 1:500 (IB) | Abcam | ab32058 |
| Rabbit anti-SENP1 | 1:1000 (IB)<br>1:50 (IHC) | Abcam | ab108981 |
| Mouse anti-SENP1 | 1:200 (IP) | Santa cruz | sc-271360 |
| Mouse anti-HA-Tag | 1:500 (IP)<br>1:3000 (IB) | Cell Signaling Technology | 2367S |
| Rabbit anti-HA-Tag | 1:500 (IP)<br>1:3000 (IB) | Cell Signaling Technology | 3724S |
| Rabbit anti-Flag-Tag | 1:500 (IP)<br>1:3000 (IB) | Cell Signaling Technology | 14793S |
| Mouse anti-Flag-Tag | 1:500 (IP)<br>1:3000 (IB) | Cell Signaling Technology | 8146S |
| Rabbit anti-His-Tag | 1:3000 (IB) | Cell Signaling Technology | 12698S |
| Mouse anti-Myc-Tag | 1:3000 (IB) | Cell Signaling Technology | 2276s |
| Rabbit anti-Myc-Tag | 1:3000 (IB) | Cell Signaling Technology | 2278S |
| Mouse anti-GST-Tag | 1:50000 (IB) | Cell Signaling Technology | 2624S |
| Rabbit anti-Phospho-(Ser/Thr) | 1:2000 (IB) | Abcam | ab300625 |

| | | | |
|---|---|---|---|
| Rabbit anti-Phospho-Serine | 1:500 (IB) | ImmuneChem | ICP9806 |
| Mouse-anti-pMLC2 (Ser19) | 1:1000(IB) 1:150(IHC) | Cell Signaling Technology | 3675S |
| Mouse-anti-GFP | 1:10000(IB) | Abmart | M20004S |
| HRP-conjugated goat anti-rabbit secondary antibody | 1:10000 | Bio-rad | 1706515 |
| HRP-conjugated goat anti-mouse secondary antibody | 1:10000 | Bio-rad | 1706516 |
| Alexa Fluor 488 goat anti-rabbit secondary antibody | 1:200 | Invitrogen | A11034 |
| Alexa Fluor 488 goat anti-mouse secondary antibody | 1:200 | Invitrogen | A11029 |
| Alexa Fluor 546 goat anti-rabbit secondary antibody | 1:200 | Invitrogen | A11035 |
| Alexa Fluor 546 goat anti-mouse secondary antibody | 1:200 | Invitrogen | A11030 |

IB, immunoblotting analysis; IF, immunofluorescence imaging analysis;
IHC, immunohistochemistry analysis; IP, immunoprecipitation.

**Table S2. Sequence information of qRT-PCR.**

| **Primers-qRT-PCR** | **5’-3’** |
|---|---|
| Human *SENP1*-F | TTGGCCAGAGTGCAAATG |
| Human *SENP1*-R | TCGGCTGTTTCTTGATTTTTGTAA |
| Human *ACTB*-F | GTGACGTTGACATCCGTAAAGA |
| Human *ACTB*-R | ATAGCACAGCCTGGATAGC |
| Mouse *Senp1*-F | AGTAAAGAAGGTTCCGGTTCCCG |
| Mouse *Senp1*-R | GCCGCCACTCACCGAAC |
| Mouse *Actb*-F | GTGACGTTGACATCCGTAAAGA |
| Mouse *Actb*-R | GCCGGACTCA-TCGTACTCC |

F, Forward primer; R, Reverse primer.

**Table S3. Information for human brain samples used in immunoblotting analysis.**

| NPDx | Gender | Age | PMI (h) | Braak Stage | CERAD |
|---|---|---|---|---|---|
| Control | F | 66 | 25.00 | NA | A |
| Control | M | 84 | 16.00 | I | A |
| Control | F | 82 | 24.42 | NA | A |
| Control | F | 73 | 20.00 | I | A |
| AD | F | 55 | 48.00 | V-VI | C |
| AD | F | 61 | 24.00 | NA | C |
| AD | F | 94 | 12.00 | V | C |
| AD | F | 77 | 17.00 | V | C |
| AD | F | 80 | 10.00 | V | C |
| AD | F | 66 | 42.00 | VI | C |
| AD | F | 80 | 19.00 | VI | C |

**Table S4. Information for human brain samples used in immunohistochemistry and immunofluorescence imaging analyses.**

| NPDx | Gender | Age | PMI (h) | Braak Stage | CERAD |
|---|---|---|---|---|---|
| Control | F | 79 | 17.58 | II | A |
| Control | F | 91 | 30.1 | II | A |
| Control | F | 63 | 26.83 | I | A |
| Control | F | 82 | 24.42 | I | A |
| AD | F | 84 | 18.33 | V | C |
| AD | F | 61 | 24.00 | VI | C |
| AD | F | 83 | 22.92 | VI | C |
| AD | F | 82 | 16.83 | VI | C |
| AD | F | 87 | 29.58 | V | C |
| AD | M | 64 | 32.00 | V | C |

NPDx, neuropsychological diagnosis; PMI, postmortem interval;
CERAD, Consortium to Establish a Registry for Alzheimer's disease.

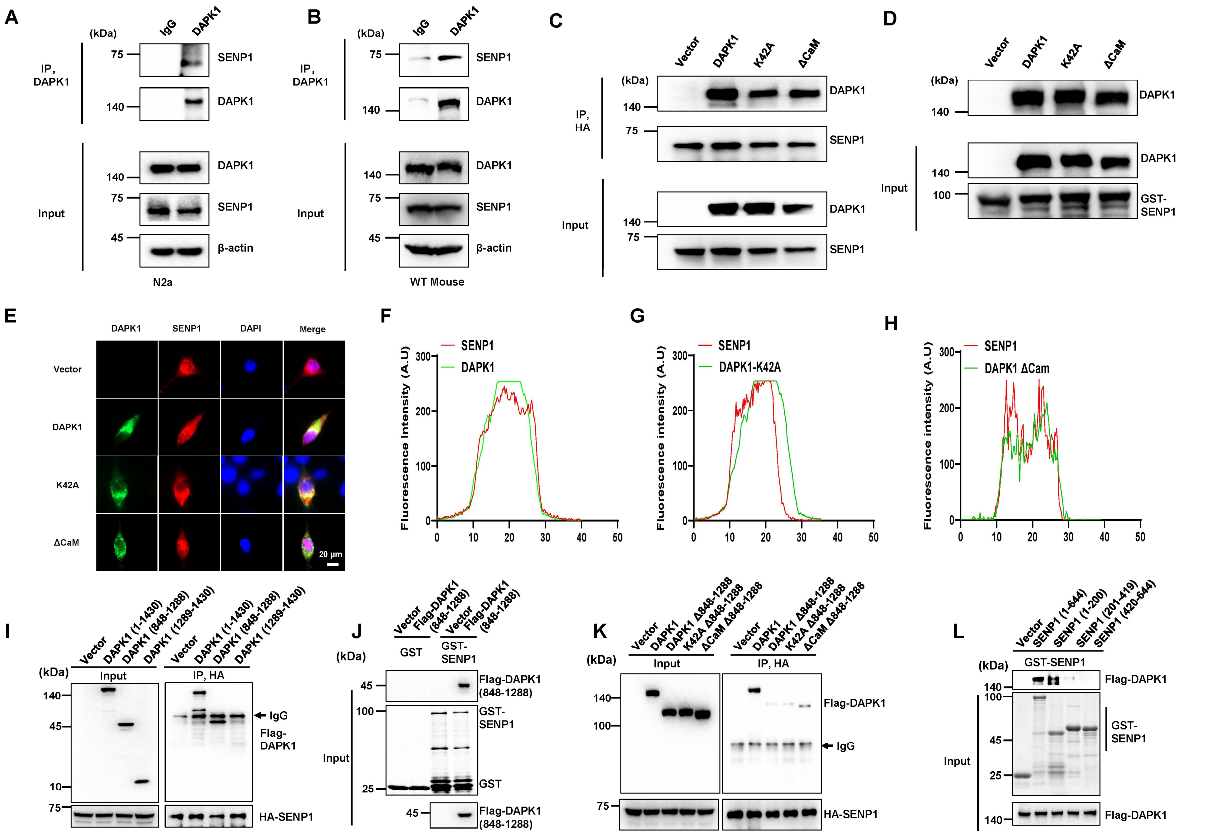


**Figure S1. DAPK1 interacts with SENP1 *in vitro* and *in vivo*.**

**(A-B)** Co-Immunoprecipitation analyses of the binding between endogenous DAPK1 and SENP1 in N2a cells and mouse brain using indicated antibodies.

**(C-D)** Cell lysates from HEK-293T cells expressing Myc-DAPK1, its kinase-impaired K42A or constitutively active ΔCaM mutants, were incubated with anti-SENP1 antibody for co-immunoprecipitation (C), or GST-SENP1 for pull-down assay (D). Samples were detected using indicated antibodies.

**(E-H)** N2a cells were co-transfected with HA-SENP1 and Myc-DAPK1, K42A or ΔCaM for 36 h. Co-localization analysis were performed using anti-HA and anti-Myc antibodies. Scale bar=20 μm.

**(I)** HA-SENP1 and full-length Flag-DAPK1 or various truncated mutants were co-expressed in HEK-293T cells. Cell lysates were then immunoprecipitated using an anti-HA antibody, followed by immunoblotting analysis using indicated antibodies.

(**J**) Cell lysates from HEK-293T cells expressing Flag-DAPK1 (848-1288 aa) were incubated with GST or GST-SENP1. Pull-down samples were detected with an anti-Flag antibody.

**(K)** Flag-DAPK1 or its mutants (K42A and ΔCaM) with the deletion of the 848-1288 aa domain was co-expressed with HA-SENP1 in HEK-293T cells. Cell lysates were then

subjected to co-immunoprecipitation using an anti-HA antibody, followed by immunoblotting analysis using indicated antibodies.

**(L)** Cell lysates from HEK-293T cells expressing Flag-DAPK1 were incubated with GST, GST-SENP1, GST-SENP1 (1-200 aa), GST-SENP1 (201-419 aa) or GST-SENP1 (420-644 aa) for pull-down assay. The pull-down samples were subjected to immunoblotting analysis with an anti-Flag antibody.

Representative images from triplicate repeats are shown.

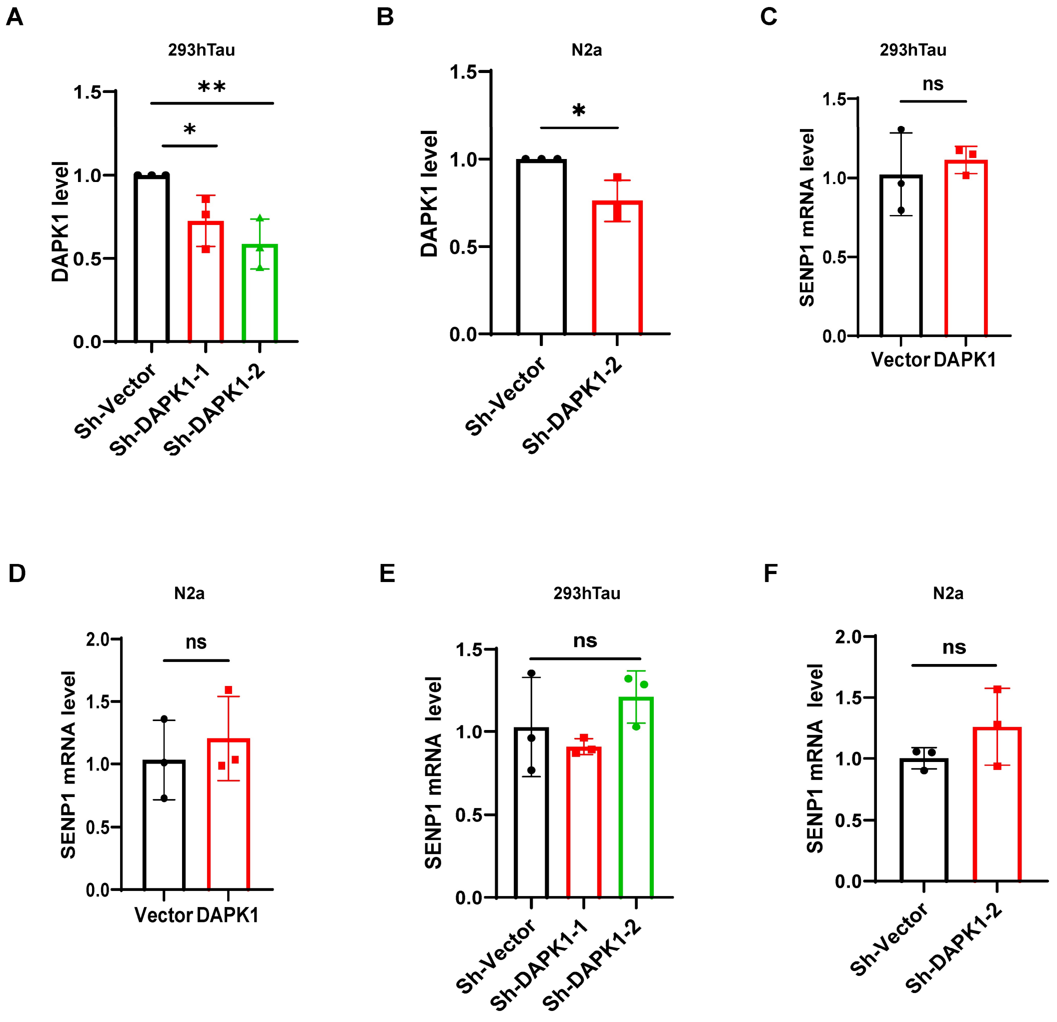


**Figure S2. DAPK1 does not influence the transcription of SENP1.**

**(A-B)** 293hTau or N2a cells were transfected with control or Flag-DAPK1, and the mRNA level of SENP1 was determined through qRT-PCR.

**(C-D)** 293hTau or N2a cells were transfected with control or DAPK1 shRNAs, and the protein expression of DAPK1 was quantified by immunoblotting analysis.

**(E-F)** 293hTau or N2a cells were transfected with control or DAPK1 shRNAs, and the mRNA level of SENP1 was determined through qRT-PCR.

Representative images from triplicate repeats are shown. *$p < 0.05$, **$p < 0.01$, ns, not significant. Two-tailed unpaired *t*-test was used in A, B, D and F, and one-way ANOVA followed by Tukey's *post-hoc* test was used in C and E.

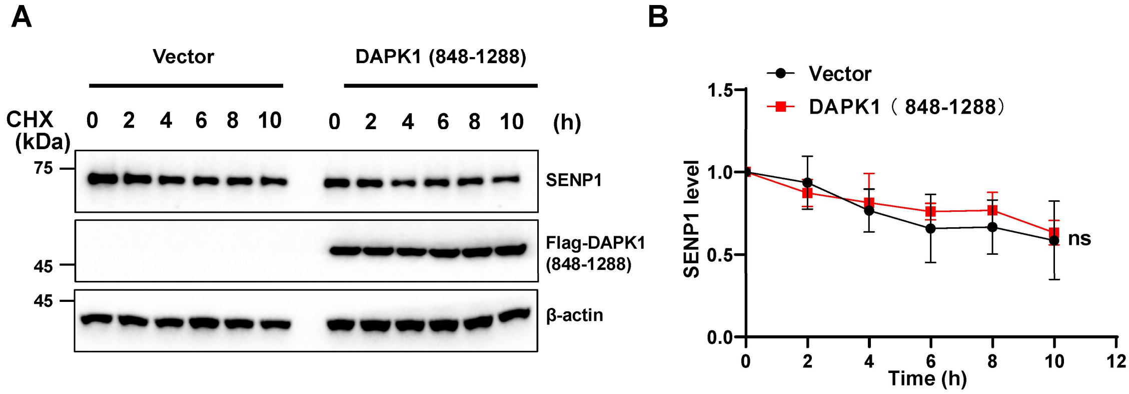


**Figure S3. The 848-1288 aa domain of DAPK1 does not affect the degradation of SENP1.** **(A-B)** HA-SENP1 was co-transfected with or without Flag-DAPK1 (848-1288 aa), and then treated with 50 μg/mL CHX for indicated time. Samples were subjected to immunoblotting analysis using indicated antibodies.

Representative images from triplicate repeats are shown. ns, not significant. Two-tailed unpaired *t*-test was used for analysis.

| Sample ID | Position | Peptide <ProteinMetricsConfidential > | Observed m/z | z | Mass error (ppm) | Score | Scan Time | Intensity |
|---|---|---|---|---|---|---|---|---|
| BTP20220729-03-01-T | 126 | R.KTS[+79.966]SGLSNSFAGK.S | 682.317 | 2 | 1.7 | 365.9 | 20.1719 | 403740000 |
| | 146 | K.SNHHC[+57.021]HVSAY[+79.966]EK.S | 774.815 | 2 | 11.8 | 302.0 | 8.8145 | 36235000 |
| | 59 | R.SFT[+79.966]C[+57.021]STR.S | 469.676 | 2 | 0.6 | 301.5 | 13.9622 | 67001000 |
| | 125 | R.KT[+79.966]SSGLSNSFAGK.S | 682.317 | 2 | 1.6 | 286.1 | 19.6616 | 403740000 |
| | 506 | K.TAGY[+79.966]QAVK.R | 459.214 | 2 | 9.6 | 229.4 | 11.6376 | 0 |
| | 548 | K.NITY[+79.966]YDSMGGINNEAC[+57.021]R.I | 686.615 | 3 | 10.5 | 144.4 | 27.1760 | 43437000 |
| | 115 | K.SRNS[+79.966]R.S | 699.290 | 1 | -4.8 | 136.3 | 17.7664 | 6628700 |

**Figure S4.** Putative phosphorylation sites of SENP1 by DAPK1 as determined by mass spectrometry.

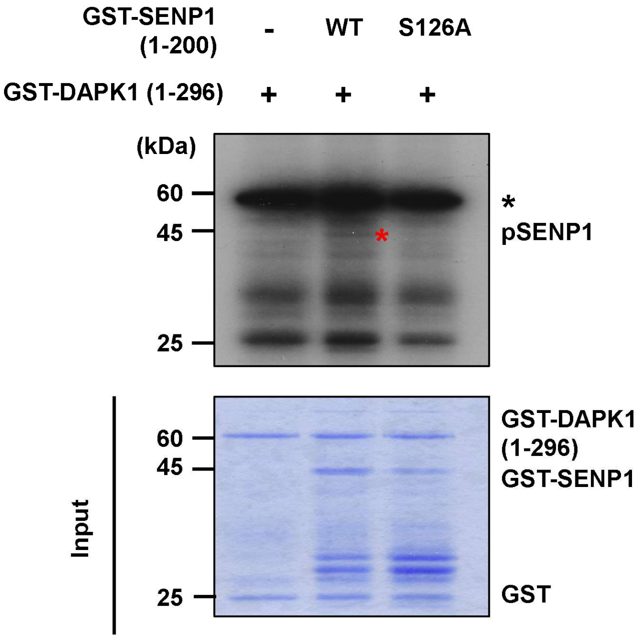


**Figure S5. DAPK1 phosphorylates SENP1 at Ser126 *in vitro*.**

An *in vitro* phosphorylation assay was performed by mixing GST-DAPK1 (1-296 aa) with GST-SENP1 (1-200 aa) or the Ser126 to Ala (S126A) mutant in the presence of $^{32}$P-ATP. Samples were subjected to autoradiography to visualize phosphorylation signals. Red asterisk indicates the SENP1 phosphorylation signal, while black asterisk marks the autophosphorylation of DAPK1. Input samples were detected by Coomassie Blue staining. Representative images from triplicate repeats are shown.

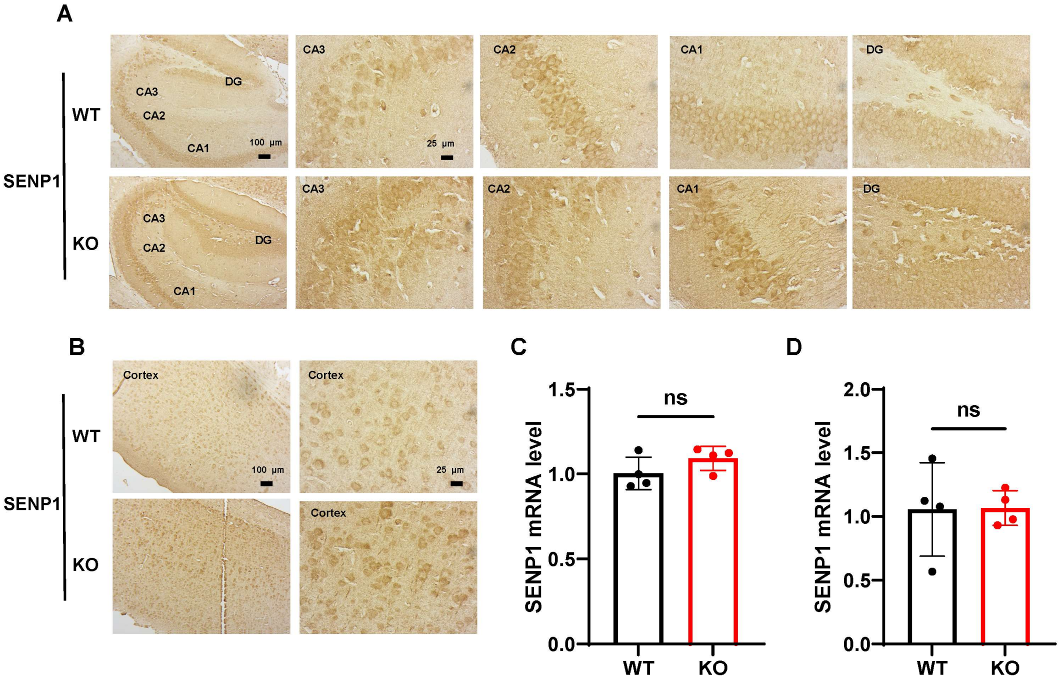


**Figure S6. SENP1 is increased in hippocampus and cortex of DAPK1-KO mice.**

**(A-B)** Brain samples from 6-mo old WT and DAPK1-KO mice were detected by immunohistochemistry using an anti-SENP1 antibody to show the SENP1 expression in hippocampus (upper panel) and cortex (lower panel). Scale bar=100 μm and 25 μm, respectively. n=4 mice/group.

**(C-D)** Brain samples from 6-mo old WT and DAPK1-KO mice were subjected to qRT-PCR analysis to determine the mRNA level of SENP1 in the hippocampus and cortex. n=4 mice/group.

Representative images from triplicate repeats are shown. ns, not significant. Two-tailed unpaired *t*-test was used for analysis.

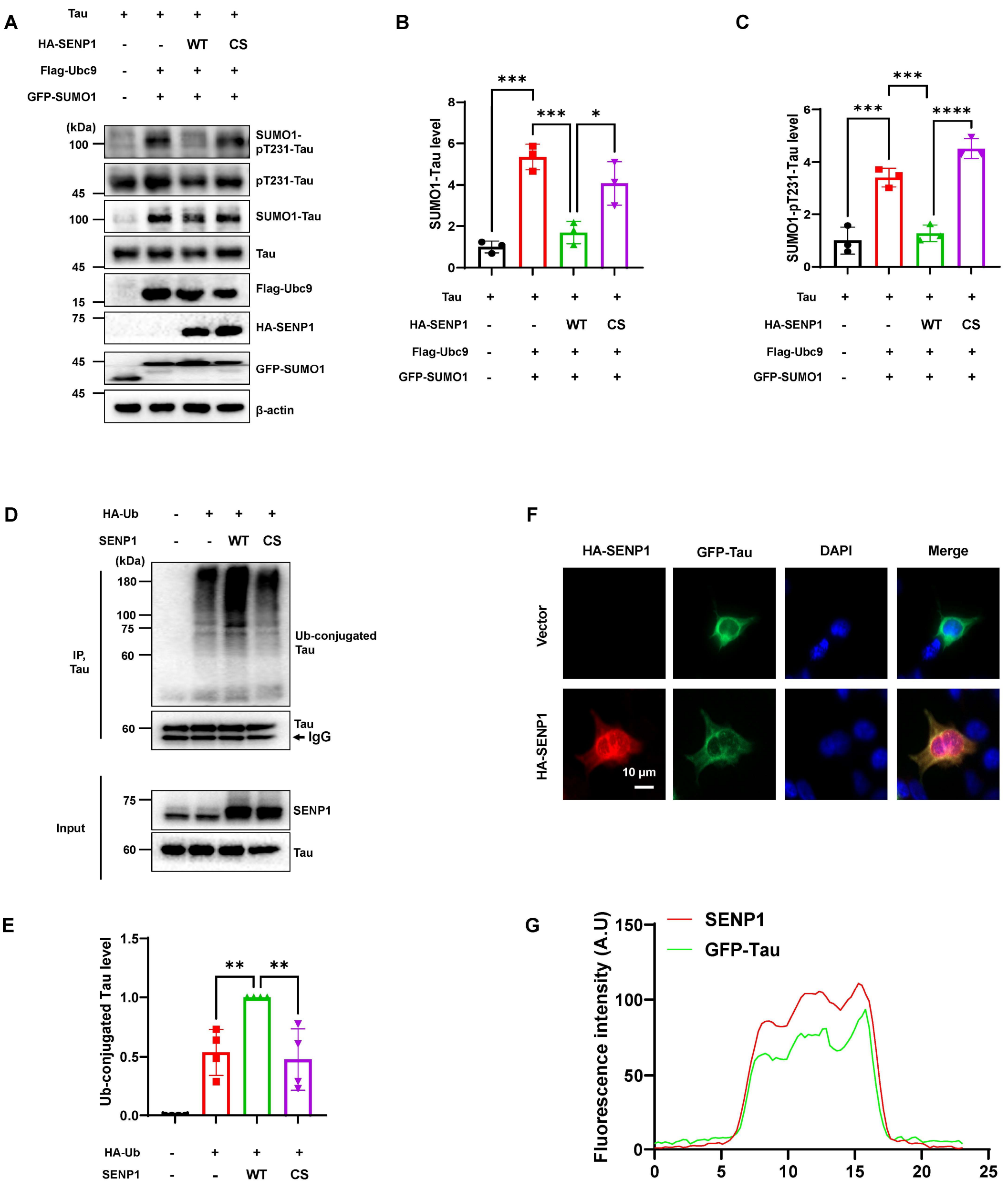


**Figure S7. SENP1 deSUMOylates tau and affects its phosphorylation and ubiquitination.**

**(A-C)** Tau was co-transfected without or with WT or CS SENP1, Flag-Ubc9 and GFP-SUMO1 in HEK-293T cells. Cell lysates were subjected to immunoblotting analysis. The SUMO1-conjuated total tau and pT231-Tau were quantified.

**(D-E)** 293hTau cells were transfected with HA-ubiquitin and WT or CS SENP1 for 48 h. Cell lysates were immunoprecipitated using an anti-Tau antibody, followed by immunoblotting analysis using an anti-HA antibody to determine tau ubiquitination. Input samples were analyzed using indicated antibodies.

**(F-G)** GFP-tau was transfected with or without HA-SENP1 in HEK-293T cells, followed by immunostaining analysis. The co-localization profile of tau and SENP1 is shown in G.

Representative images from triplicate repeats are shown.

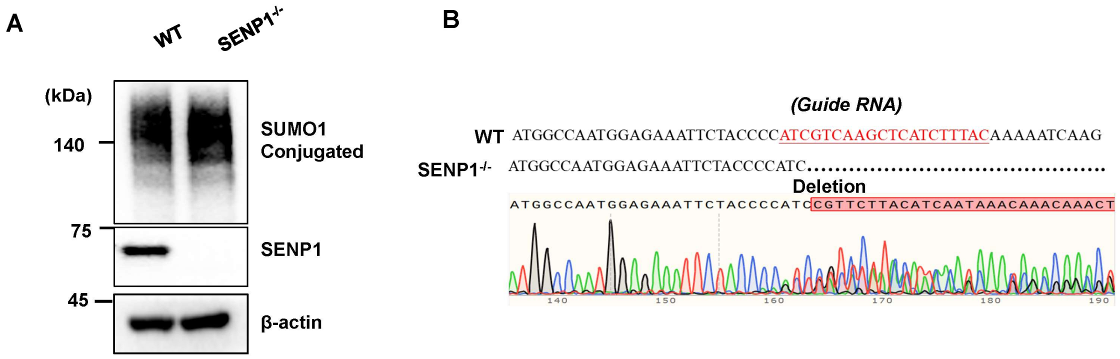


**Figure S8. Construction and validation of SENP1 knockout cell line.**

**(A)** Immunoblotting analysis of SENP1 expression and SUMO1-conjugated protein levels in control and SENP1 knockout (SENP1$^{-/-}$) cells. Representative images from triplicate repeats are shown.

**(B)** Validation of SENP1 knockout by DNA sequencing.

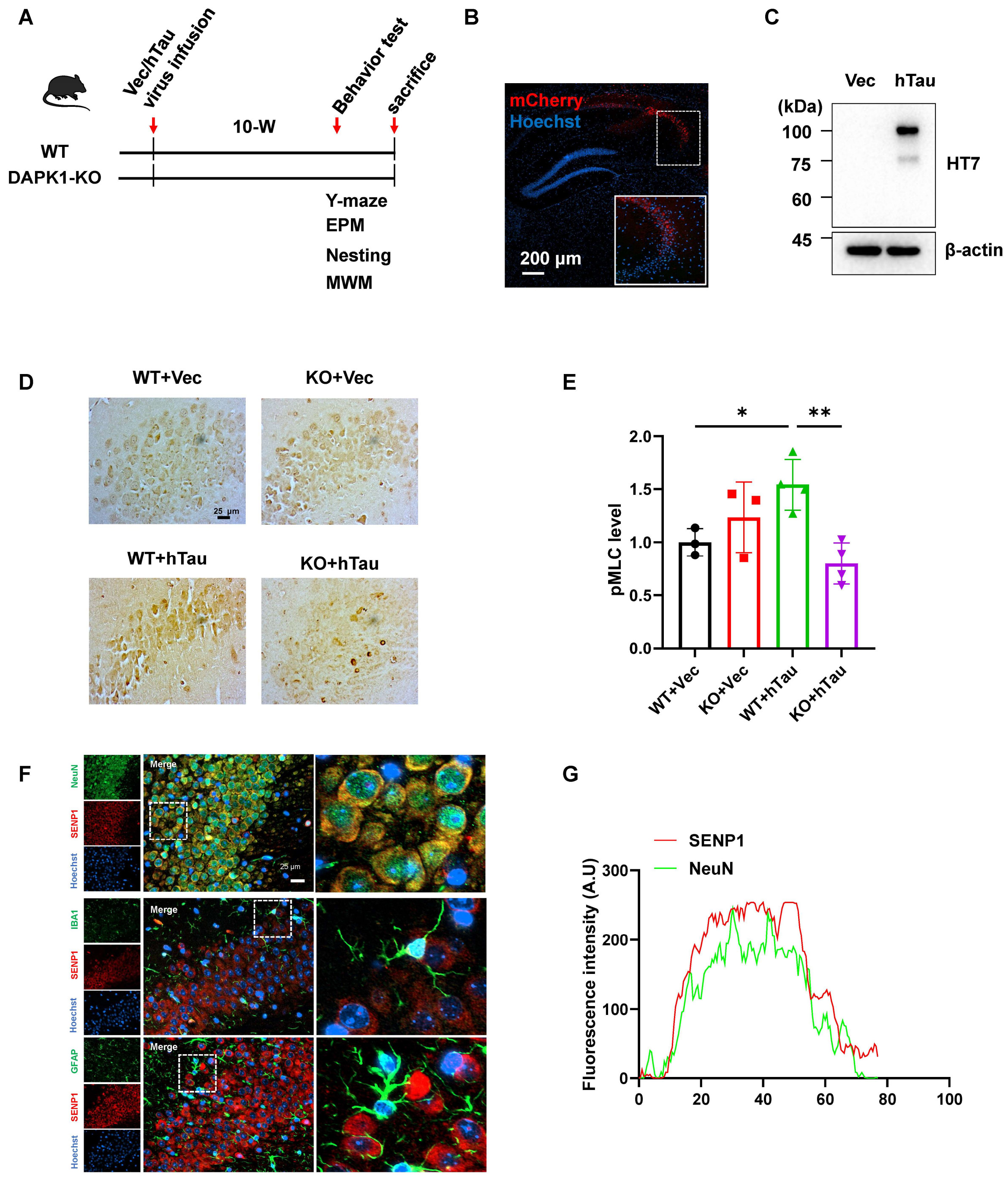


**Figure S9. Establishment of the mouse model of tauopathy and the characterization of SENP1 expression in different cell types in the brain.**

**(A)** Schematic showing the time line of the mouse experiments.

**(B-C)** Immunofluorescence imaging and immunoblotting analysis of hTau expression in the hippocampus in mouse models infused with the hTau virus. Insert shows an amplified area with extensive mCherry expression in CA3. Scale bar=200 μm.

**(D-E)** Immunohistochemistry of the phosphorylation of MLC at Ser19 (pMLC), a canonical DAPK1 substrate of DAPK1, in the hippocampal CA3 area in WT or DAPK1-KO mice injected with vector or hTau virus. Scale bar=25 μm.

**(F-G)** Co-localization of SENP1 with NeuN (neuronal marker), GFAP (astrocyte marker) or IBA1 (microglial marker) in hippocampal CA3 area by immunofluorescence imaging.

Representative images from triplicate repeats are shown. $^{*}p < 0.05$, $^{**}p < 0.01$. One-way ANOVA followed by Tukey's *post-hoc* test was used for statistics.

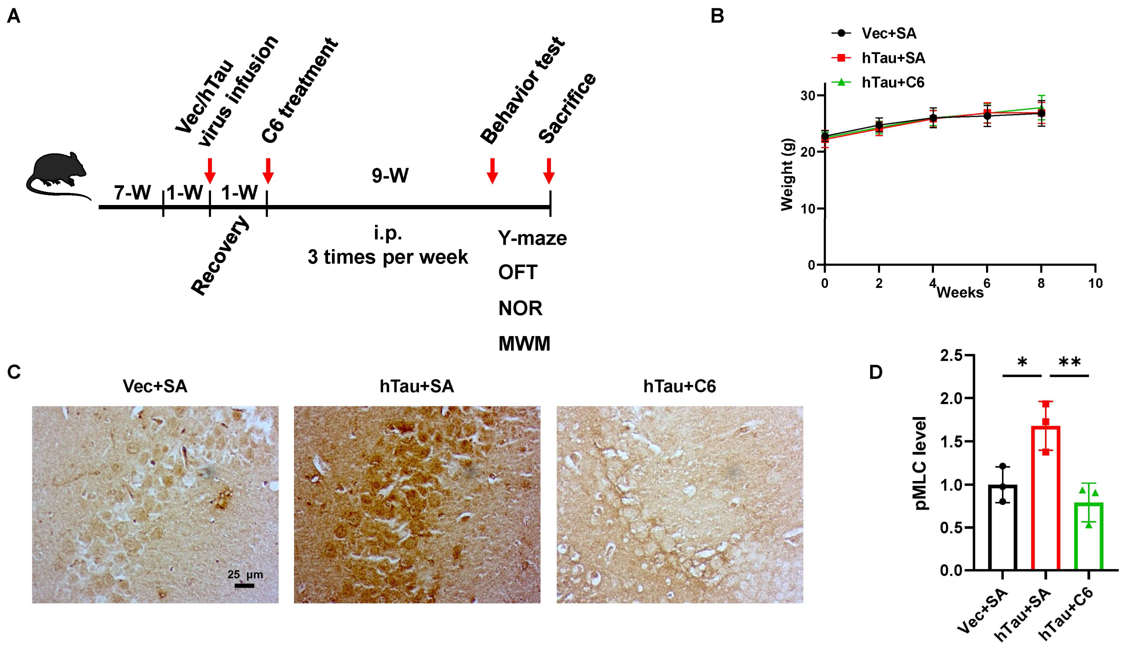


**Figure S10. Characterization of body weight change and DAPK1 activity in mice treated with or without the DAPK1 inhibitor C6.**

**(A)** Schematic showing the time line of the mouse experiments.

**(B)** Body weight change in mice treated with or without the DAPK1 inhibitor C6.

**(C-D)** Immunohistochemistry of the phosphorylation of MLC at Ser19 (pMLC) in the hippocampal CA3 area in mice treated with the DAPK1 inhibitor C6 or saline (SA). Scale bar=25 μm. Representative images from triplicate repeats are shown. $*p < 0.05$, $**p < 0.01$. One-way ANOVA followed by Tukey's *post-hoc* test was used for analysis.
